# Targeting SHP2 Cryptic Allosteric Sites for Effective Cancer Therapy

**DOI:** 10.3390/ijms25116201

**Published:** 2024-06-04

**Authors:** Ashfaq Ur Rehman, Cizhang Zhao, Yongxian Wu, Qiang Zhu, Ray Luo

**Affiliations:** Departments of Molecular Biology and Biochemistry, Chemical and Biomolecular Engineering, Materials Science and Engineering, and Biomedical Engineering, University of California, Irvine, CA 92697, USA; aurehman@uci.edu (A.U.R.);

**Keywords:** protein tyrosine phosphatase, structure dynamics, multi-scale docking, computational chemistry, molecular dynamics

## Abstract

SHP2, a pivotal component downstream of both receptor and non-receptor tyrosine kinases, has been underscored in the progression of various human cancers and neurodevelopmental disorders. Allosteric inhibitors have been proposed to regulate its autoinhibition. However, oncogenic mutations, such as E76K, convert SHP2 into its open state, wherein the catalytic cleft becomes fully exposed to its ligands. This study elucidates the dynamic properties of SHP2 structures across different states, with a focus on the effects of oncogenic mutation on two known binding sites of allosteric inhibitors. Through extensive modeling and simulations, we further identified an alternative allosteric binding pocket in solution structures. Additional analysis provides insights into the dynamics and stability of the potential site. In addition, multi-tier screening was deployed to identify potential binders targeting the potential site. Our efforts to identify a new allosteric site contribute to community-wide initiatives developing therapies using multiple allosteric inhibitors to target distinct pockets on SHP2, in the hope of potentially inhibiting or slowing tumor growth associated with SHP2.

## 1. Introduction

The SHP2, a non-receptor protein tyrosine phosphatase encoded by PTPN11, is integral to the developmental processes mediated by growth factors, cytokines, and adhesion receptors. SHP2 is ubiquitously expressed and is essential for the sustained activation of the Ras-MAP kinase pathway, which plays a crucial role in cell proliferation, differentiation, and survival. Additionally, SHP2 modulates several other critical signaling pathways, including NF-κB, JAK-STAT, PI3K-AKT, PD-1, and various immune checkpoints, which are involved in immune regulation and cell growth [1,2,3,4].

Activating mutations in SHP2 are implicated in developmental disorders such as Noonan Syndrome [5] and are frequently observed in juvenile myelomonocytic leukemia (35%), and, to a lesser extent, in acute myeloid leukemia (5%) [6]. These mutations are also present at lower frequencies in other hematologic and solid tumors [4] and have been shown to induce leukemia in murine models [7]. Conversely, inhibiting SHP2 demonstrates antitumor activity across various cancer models [8,9].

**Figure 1:**
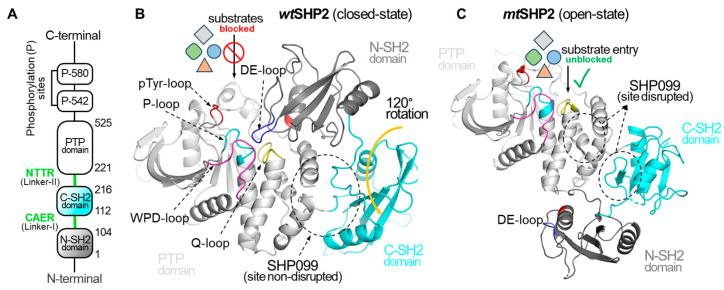
Overview of Structural Dynamics of the SHP2 Protein. (**A**) depicts the domain organization of the full-length SHP2 protein. (**B**) illustrates SHP2 in its closed or basal state, PDB ID: **2SHP** [10]. In the closed state, the DE-loop of the N-SH2 domain occludes the PTP domain catalytic cleft, effectively preventing the binding of diverse phosphorylated substrates and small molecules, and affecting the SHP2 function. (**C**) showcases the open state of SHP2, PDB ID: **6CRF** [11], induced by the oncogenic mutation (E76K). The mutation causes a 120-degree C-SH2 domain rotation, exposing the catalytic cleft and shifting the N-SH2 domain across the PTP domain. In the open state, the DE-loop of the N-SH2 domain no longer interacts with the catalytic cleft of the PTP domain, resulting in complete access to the catalytic cleft for pY-ligands. Additionally, this gain-of-function mutation compromises the binding cavity for SHP099.

Structurally, SHP2 comprises two tandem Src homology 2 domains (N-SH2 and C-SH2), a protein tyrosine phosphatase (PTP) domain, and a disordered C-terminal tail containing two phosphotyrosine-binding sites (Figure 1) [10]. The enzyme dynamically transitions between a closed (basal) state, where the N-SH2 domain occludes the PTP catalytic cleft, and an open (active) state, triggered by activating mutations or ligand binding [11,12]. This allosteric transition, characterized by a substantial 120-degree rotation of the C-SH2 domain, represents a conformational change of unusual magnitude that has garnered significant research attention [11,12,13,14]. Nuclear magnetic resonance (NMR) spectroscopy and molecular dynamics simulations have been instrumental in elucidating the key transition states and pathways underlying SHP2’s allosteric regulation [12,15,16,17].

Recent investigations, employing molecular dynamics simulations and small-angle X-ray scattering (SAXS), have revealed unexpected flexibility within the open state of the E76K mutant, a model system for studying SHP2 activation [16,18,19,20]. Notably, molecular dynamics simulations show that the E76K solution structure deviates significantly from the crystal structure [18]. Furthermore, Anselmi and Hub reported a heterogeneous atomistic ensemble of the E76K mutant in solution, in excellent agreement with SAXS data [20]. These observations raise questions about the factors contributing to this flexibility, including potential intrinsic domain instability or the influence of crystal packing effects.

The development of allosteric inhibitors that stabilize the closed state of SHP2 has shown promise in clinical trials for advanced or metastatic solid tumors [21,22,23,24,25,26,27,28,29,30]. These “molecular glue” inhibitors, like SHP099, target an allosteric site formed between the C-SH2 and PTP domains, demonstrating high-affinity binding and antiproliferative effects in certain cancer cell lines [8]. However, activating mutations can destabilize the closed state, leading to reduced inhibitor efficacy and drug resistance [31]. While a dual inhibition strategy targeting an additional allosteric site at the N-SH2/PTP interface has been proposed [32], the dynamic nature of SHP2 poses challenges for inhibitor binding, highlighting the need for more robust allosteric inhibitors.

In light of these challenges, this study aims to elucidate the origin of the observed flexibility within the open state of SHP2, particularly in the context of the E76K mutant. We will also investigate the impact of activating mutations on the efficacy of existing allosteric inhibitors. These insights will contribute to the development of more effective therapeutic strategies targeting SHP2, including the identification of novel allosteric sites less susceptible to the effects of activating mutations.

## 2. Results and Discussion

In this study, we employed molecular dynamics (MD) simulations to investigate the solution structures and dynamic stability of SHP2 in both its wild-type (closed state, *wt*SHP2) and E76K mutant (open state, *mt*SHP2) forms. Simulations were conducted using the FF19SB and GAFF2 force field in the TIP3P water and 298 K. Three production trajectories of 500 ns each were collected on both monomeric (chain A and chain B) and dimeric (chain A + chain B) configurations. To assess the impact of ligand binding, we also simulated systems with the allosteric inhibitor SHP099 (chain A), also in three replicas of 500 ns each. Initial structures for all simulations were derived from available SHP2 crystal structures. A total of approximately 28.6 µs of MD trajectories were collected in the explicit water model (see Supplementary Table S1 for details).

Our analysis first focused on the dynamic stability of individual domains (N-SH2, C-SH2, and PTP) to quantify the increased flexibility observed in the E76K open state. As detailed in the Supplementary Information (Figure S1), each domain within *wt*SHP2 exhibited low RMSD values, indicating remarkable stability. This is likely due to the compact, tightly packed arrangement of the protein in the closed state. In contrast, while the N-SH2 and PTP domains of *mt*SHP2 maintained RMSD values comparable to *wt*SHP2, the C-SH2 domain displayed significantly higher fluctuations, suggesting heightened flexibility. This observation aligns with the lower certainty of the C-SH2 domain, which reflects its relatively looser packing environment in the crystal lattice, both intermolecularly and intramolecularly.

We further explored the role of crystal packing environment, a notable factor influencing structural variations in MD simulations. While four monomers are organized linearly in a relatively straightforward packing pattern within the *wt*SHP2 unit cell, eight monomers arrayed in a more complex packing formation within the *mt*SHP2 unit cell (Supplementary Information, Figure S2). Obviously, the *wt*SHP2 unit cell closely mirrors our solvated monomer/dimer MD simulation environment. In contrast, the *mt*SHP2 asymmetric unit exhibits a more complex packing arrangement within the unit cell, leading to solvated monomer/dimer MD simulations that do not fully capture this intricate packing. Thus, the high variability in C-SH2 domain dynamics in *mt*SHP2 simulations may be partly due to the absence of crystal packing constraints. Conversely, the lower C-SH2 variability in *wt*SHP2 simulations could be attributed to solvation effects that better mimic the *wt*SHP2 crystal environment.

Given that the observed variations in individual domain dynamics are primarily attributed to differences in crystal packing, the success of allosteric modulators in shifting the open state structure towards the closed state is reinforced. The primary role of these modulators is to act as “molecular glues”, stabilizing inter-domain interactions.

### 2.1. Impact of the Oncogenic Mutation E76K on Allosteric Inhibition Sites

**Figure 2:**
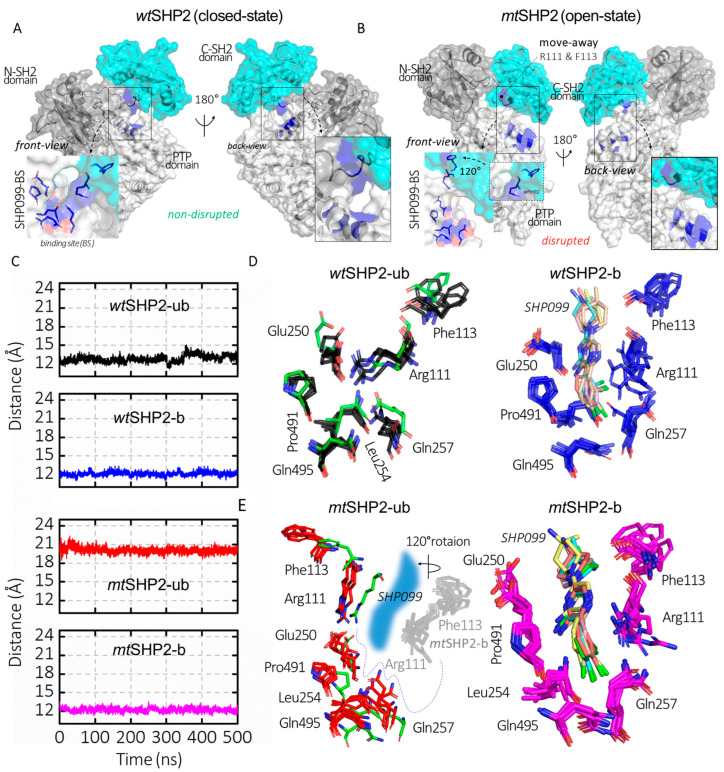
Analysis of SHP099 binding site dynamics across all SHP2 simulations. (**A**,**B**) depict SHP2 in its closed (*wt*SHP2) and open (*mt*SHP2) conformations. In the closed state, SHP099 binds from the back side, while the front-side access is hindered by a short disordered loop. However, mutation E76K alters the binding pocket through a rotation in the C-SH2 domain, resulting in the displacement of key residues Arg111 and Phe113, which play a role in the creation of the binding pocket for SHP2 inhibitors. (**C**) represents the CA distances between Arg111 and Phe113 that are involved in SHP099 binding across all SHP2 configurations. (**D**,**E**) illustrate the superimposed structures from the top MD solution clusters of each SHP2 system, while the binding site residues form a consistent cavity in the *wt*SHP2 and *mt*SHP2-b states, a discernible shift in two residues from the C-SH2 domain away from the binding pocket is evident for the pocket disruption in the *mt*SHP2-ub conformation. The green sticks denote the crystallographic structure from the *wt*SHP2-b state.

Our study extends to the functional disruption of the E76K mutation on SHP2’s allosteric inhibition. Traditionally, allosteric SHP2 inhibitors have focused on the *allo*-site-1 pocket [8], which is formed by residues from both the C-SH2 and PTP domains, such as Arg111, Phe113, Glu250, Leu254, Gln257, Pro491, and Gln495 (Figure 2D,E). These inhibitors function by stabilizing SHP2 in its closed state [21,22,23,24,25,26,27,28,29,30]. Through monitoring the CA distance between Arg111 (on C-SH2 domain) and Phe113 (on PTP domain) across various SHP2 states, we observed a uniform distance in all closed conformations, including *wt*SHP2-ub, *wt*SHP2-b, and *mt*SHP2-b (Figure 2C). However, the open state, influenced by the E76K mutation, exhibits a pronounced CA distance deviation, correlating with the C-SH2 domain’s 120-degree rotation (Figure 2A,B) [11,12]. This movement signifies the *allo*-site-1 pocket disruption upon SHP2 activation.

A novel dual inhibition strategy has been proposed for more effective SHP2 inhibition, targeting a second pocket, *allo*-site-2, at the interface of the N-SH2 and PTP domains (Supplementary Figure S3) [32]. Despite this, achieving potent inhibition remains challenging, indicating the potential for improved inhibitor design. We hypothesized that the reduced efficacy of inhibitors at pocket *allo*-site-2 could be due to its transient stability. The pocket is flanked by key residues Glu83 and Arg265, which may predispose the site to inhibitor dissociation.

**Figure 3:**
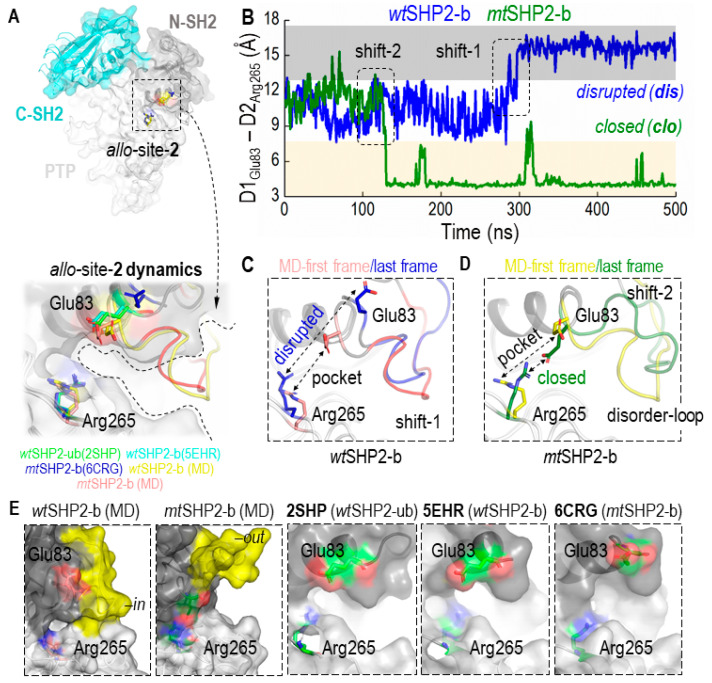
Conformational Dynamics of the Allosteric Site-2 (*allo*-site-2) Pocket in SHP2**.** (**A**) shows the superimposition of all closed-state MD solution structures, including *wt*SHP2-ub/b and *mt*SHP2-b, along with their corresponding crystal structures 2SHP, 5EHR, and 6CRG, respectively, revealing the pocket’s conformational consistency. (**B**) provides a distance analysis between two salt-bridging residues flanking pocket *allo*-site-2. In *wt*SHP2-b, the two residues start from their initial stable state but transition into a disrupted state, leading to a flat binding surface. In *mt*SHP2-b, the two residues transition into a closed state, forming a salt bridge and capping the binding pocket. (**C**,**D**) illustrate conformational variations observed in the simulations: Shift 1 in a *w*tSHP2-b simulation and Shift 2 in an *mt*SHP2-b simulation. (**E**) showcases the surface representation of *allo*-site-2. In MD solution structures, the disordered loop stays either in an ‘in’ conformation in wt-SHP2-b, capping *allo*-site-2, or in an ‘out’ conformation in mt-SHP2-b, allowing ligand-binding. The three crystal structures used in MD simulations are shown along with the two MD solution structures. Note that the disordered loop is missing in all three crystal structures.

To evaluate this hypothesis, we assessed the CA distance dynamics between Glu83 and Arg265, which are crucial to the structure of the *allo*-site-2 pocket. As shown in Figure 3A–D, these residues initially appear to be stable, maintaining the pocket’s integrity. However, the wild type complex (*wt*SHP2-b) gradually transitions into a disrupted state with a flat surface after ~300 ns, and the mutant complex (*mt*SHP2-b) transitions into a state with a salt bridge after ~100 ns, though occasional fluctuations back to the initial state are also visible. These conformational changes underscore the transient nature of *allo*-site-2’s pocket stability. When these residues are close together, the ligand cannot bind. When these residues move apart, the pocket becomes a flat surface, both compromising the pocket’s binding potential and resulting in the inhibitors showing less potency toward this site.

An area adjacent to *allo*-site-2, comprised of the disordered loop spanning residues His85–Asp90 and Gln92–Val95, also appears to be significant in ligand binding (Figure 3E, highlighted in yellow). We observed this loop oscillating between two distinct states: an ‘in’ conformation that acts as a cap, potentially sealing off the pocket (*wt*SHP2-b), and an ‘out’ conformation that opens *allo*-site-2, facilitating inhibitor binding (*mt*SHP2-b). This loop’s flexibility introduces a steric barrier that could deter the binding of a second inhibitor to *allo*-site-2, thereby influencing the overall inhibitor-enzyme interaction dynamics.

In summary, the structural dynamics of Glu83 and Arg265, along with the flexible loop adjacent to *allo*-site-2, play critical roles in the pocket’s stability and accessibility. These factors must be considered in the design of more effective SHP2 inhibitors.

### 2.2. Identification of a New Allosteric Pocket

Allosteric inhibitors traditionally bind to SHP2 through a conformation-selection mechanism, selectively targeting the protein’s closed state [21,22,23,24,25,26,27,28,29,30]. Regrettably, the E76K activating mutation significantly diminishes the binding affinity of inhibitors targeting *allo*-site-1 by approximately 100-fold [11,12]. Dual inhibition strategies could markedly improve efficacy against the mutant SHP2 state, as opposed to the singular action of SHP099, which immobilizes SHP2 in its closed conformation [32]. As previously mentioned, the *allo*-site-1 pocket, formed at the interface of the C-SH2 and PTP domains, is effectively bound by SHP099 [8]. However, the second pocket, *allo*-site-2, demonstrates dynamical instability, which may lead to only marginal stabilization when leveraged for dual inhibition.

We postulate the presence of additional allosteric sites that could be harnessed to stabilize the closed state of SHP2, particularly to counteract the hyperactive E76K mutant’s effects. Therefore, discovering a novel secondary binding pocket is a key objective of our research aimed at achieving potent and comprehensive inhibition of SHP2.

For reliable pocket prediction, we employed a three-prong approach: (1) AlloSite v2.0 web-based server (https://mdl.shsmu.edu.cn/AST/Allosite/index.jsp, accessed on 7 March 2023), (2) AutoSite v1.0 software package [33], and (3) visual inspection. The first two strategies identified not only established allosteric pockets but also revealed a previously unrecognized pocket—termed the *allo*-closite pocket—at the junction of the N-SH2, C-SH2, and PTP domains, distinct from the SHP099 binding site (Supplementary Figure S3). The third approach involved manual examination of potential pockets in all SHP2 closed conformations based on surface analysis. Upon comparing the *allo*-closite pocket in both open and closed crystal structures of SHP2, we observed its absence in the initial crystal structures, despite its stability in MD solution structures of closed SHP2 states. The *allo*-closite pocket encompasses residues His8, Asn10, Ile11, Thr12, Val14, Glu15, Asn18, Leu19, Thr22, Arg23, Asn103, Cys104, Ala105, Asp106 (N-SH2 domain); Pro144, Phe147, Cys174 to Tyr179, Asp188, Ser189 (C-SH2 domain); and Thr239 to Lys242 of the PTP domain.

**Figure 4:**
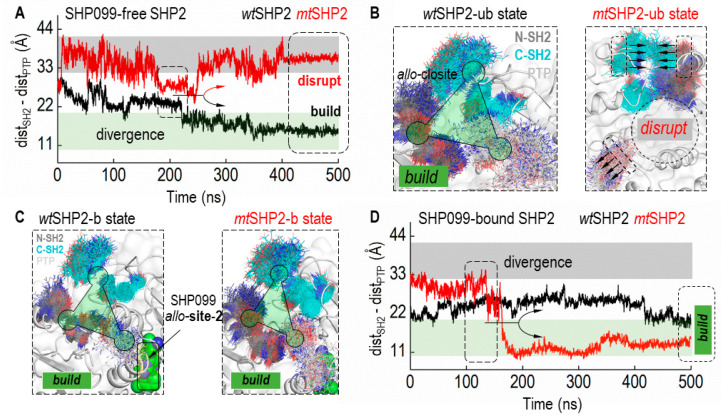
Dynamics of the *allo*-closite Pocket in SHP2 States. (**A**) illustrates the Cα distance (CA-dist) among key residues from the SH2 and PTP domains that constitute the *allo*-closite pocket in SHP2 simulations unbound to SHP099. Variations in CA-dist provide insights into the pocket’s integrity, where increased distances suggest disruption, and decreased distances indicate pocket formation. Converging CA-dist values are indicative of *allo*-closite pocket establishment. (**B**) displays superimposed structural snapshots demonstrating the *allo*-closite pocket’s conformational shifts in SHP099-free SHP2 simulations. (**C**) showcases superimposed snapshots in SHP99-bound SHP2 simulations, underscoring the enhanced stability and potential therapeutic significance of the *allo*-closite pocket. (**D**) presents the CA distance (CA-dist) plot for SHP2 simulations when bound to SHP099, which aids in assessing the pocket dynamics upon inhibitor binding.

Integrating these findings, we delved into the dynamics of the *allo*-closite pocket, both with and without the SHP099 inhibitor, to confirm its stability in all closed states of SHP2 (Figure 4). Based on CA distances between contributing SH2 and PTP domain residues, our structural analysis revealed that the *allo*-closite pocket forms robustly in all closed SHP2 states, with constituent residues coalescing to establish a well-defined pocket. Conversely, in the *mt*SHP2 state unbound to SHP099, we detected dynamic convergence of the SH2 domains and divergent movement of the PTP domain residues. After a 250 ns simulation (Figure 4A), a notable CA distance divergence among the pocket-forming residues in *mt*SHP2 unbound to SHP099 indicated pocket destabilization (Figure 4B). Intriguingly, when bound to SHP099 (Figure 4C,D), the *allo*-closite pocket exhibited greater stability—surpassing even the apo *wt*SHP2—highlighting the potential exclusivity of our identified pocket in the closed state and its selective affinity, akin to that of SHP099.

### 2.3. Extensive Multi-Tiered Screening for Hit Compound Discovery

#### 2.3.1. Hit Compound Identification

Our newly identified pocket underwent virtual screening against a cancer-specific small molecule library from the NCI Open Database (https://cactus.nci.nih.gov/download/nci/index.html, accessed on 28 March 2023). This rigorous process involved a three-tiered screening approach designed to pinpoint potential hit compounds. Following a consensus selection process after Tier I and Tier II screenings, the top 2000 compounds for both *wt*SHP2 and *mt*SHP2 receptors were determined (Supplementary Table S2), leading to the identification of 73 common top-hit compounds (Supplementary Table S3). Because of large amounts of polar and charged residues in the *allo*-closite and the pocket’s shape, these top hits underwent further scrutiny by visual inspection of their interactions with pocket residues and their drug likeness, resulting in a selection of 18 promising, relatively planar candidates for detailed analysis (Table 1), hereafter referred to as theoretical binding affinity (*t*-BA) hits, as their selection was primarily based on their predicted *t*-BA values.

In pursuit of a broader scope of candidate compounds, we expanded our visual inspection beyond the top 2000 compounds to include those that did not reach the highest ranks. This comprehensive evaluation considered the drug likeness, the binding orientations, and interactions with critical residues across the three domains, recognizing that the molecule’s alignment is pivotal for effectively stabilizing the domain interfaces. This exhaustive analysis yielded 21 additional potential compounds (Table 2). When contrasting these compounds, referred to as human-visualized interaction (*h*-VI) hits, with the initial set of *t*-BA hits (Table 1), no overlap was observed. Yet, a comparison with the 73 *t*-BA hits from Supplementary Table S3 unveiled five shared hit compounds, demonstrating some convergence between the selection methodologies.

#### 2.3.2. Protein–Ligand Interaction Profile Analysis

Next, we conducted an in-depth analysis of the protein–ligand interaction (PLI) profiles for the 39 compounds selected through both *t*-BA and *h*-VI criteria. This examination began with the top-hit compounds identified based on theoretical binding affinity (*t*-BA). The PLI profiles for *wt*SHP2-b (Supplementary Figure S4) revealed predominant interactions between the compounds and key pocket residues, including Asn10 and Glu15 from the N-SH2 domain, along with Asp106, Glu139, Pro144, Phe147, Cys174, Gln175, Glu176, and Leu177 from the C-SH2 domain. For *mt*SHP2-b (Supplementary Figure S5), the PLI profiles pinpointed critical interactions within the pocket, notably with His8, Glu15, Asn18, Arg23 from the N-SH2 domain; Asp106, Glu176 from the C-SH2 domain; and Asp241, Lys242 from the PTP domain.

For the compounds chosen based on human visualization interaction (*h*-VI) criteria, the PLI profiles against *wt*SHP2-b (Supplementary Figure S6) indicated that the compounds predominantly interact with Asn10, Lys35 from the N-SH2 domain; Asp106, His116, Glu139, Pro144, Phe147, Gln175, Glu176, and Leu177 from the C-SH2 domain; and Glu238, Thr240, Lys244, Glu249 from the PTP domain. The PLI profiles for *mt*SHP2-b (Supplementary Figure S7) highlighted key compound interactions with residues His8, Glu15, Leu19, Thr22, Arg23 from the N-SH2 domain; Asp106, Glu176, Tyr179 from the C-SH2 domain; and Asp241 from the PTP domain.

In summary, nearly all 39 compounds meeting either *t*-BA or *h*-VI criteria form hydrogen bonds with multiple residues across different protein domains. Furthermore, many of these compounds contain aromatic rings, facilitating additional hydrophobic or cation-π interactions.

#### 2.3.3. Potential Binding to Allo-Site-1

For comparison, we also conducted docking analysis of the 39 selected compounds against the *allo*-site-1 pocket (on the receptor structure without SHP099, as in Tier I). The analysis details and docking results are provided in Tables S4 and S5, respectively. Notably, none of the 39 compounds exhibited high affinity for *allo*-site-1, when compared with the binding affinity of −13 kcal/mol for SHP099 in the crystallographic pose [8]. Upon visualization of the binding poses, only NSC-163300, NSC-637201, NSC-39918, and NSC-63675 were successfully inserted into the tunnel-like pocket. These compounds also displayed the lowest predicted binding affinities among the 39 compounds tested. This comparative analysis suggests that these compounds are less likely to compete effectively for binding to *allo*-site-1, supporting our goal of using the compounds in a dual allosteric inhibition scheme along with SHP099.

#### 2.3.4. Stability Analysis via MD Simulations in Tier III Screening

Our Tier III screening involved MD simulations of complexes with selected hit compounds. We conducted a total of 78 MD simulations for *wt*SHP2- and *mt*SHP2-ligand systems to assess the stability of these complexes. Each hit compound complex underwent a 100 ns production MD simulation, which allowed us to weed out weak binders within the *allo*-closite pocket.

**Figure 5:**
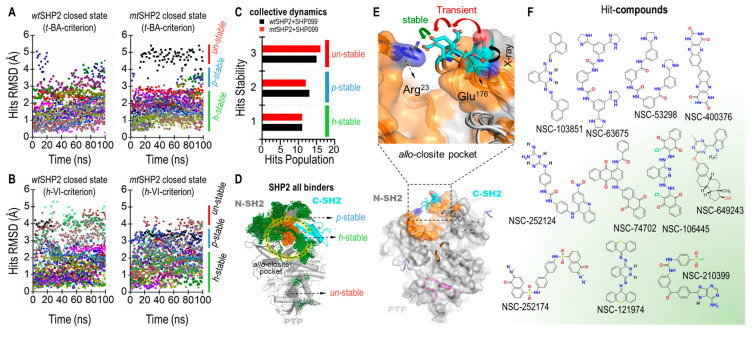
Tier III MD screening of common compounds for SHP2 closed states. (**A**) Ligand RMSD for *wt*SHP2 and *mt*SHP2 based on *t*-BA criteria. (**B**) Ligand RMSD based on *h*-VI criteria. (**C**) Stability distributions in collective MDs. (**D**) Superposition of all ligands to show the ligand distributions in the *allo-*closite pocket. (**E**) Conformational change of the *allo-*closite gate-keeper residues, forming salt bridge upon binding of hit compounds. (**F**) 2D structures of 11 stable common compounds.

To gauge their dynamic stability, we monitored the root-mean-square deviation (RMSD) of each hit compound. We established a threshold RMSD of 3 Å as the stability criterion; compounds exhibiting an RMSD less than this value were deemed stable and were selected for further scrutiny. Our visual analysis indicated that compounds with RMSD under 3 Å maintained significantly greater stability and consistent orientation within the pocket compared to those with RMSD values above 3 Å (Figure 5A,B).

Our findings identified 11 highly stable, 13 partially stable, and 15 unstable hit compounds for *wt*SHP2. Correspondingly, for *mt*SHP2, we discovered 11 highly stable, 12 partially stable, and 16 unstable compounds. Notably, the highly stable compounds demonstrated similar stability profiles in both *wt*SHP2 and *mt*SHP2, suggesting a favorable selectivity for the closed state of SHP2 (Figure 5C,D). The partially stable and unstable compounds, however, exhibited variability between the two receptor states.

Additionally, we extended the production molecular dynamic (MD) simulations for the 11 highly stable compounds up to 500 ns as the crystal structures and analyzed their RMSD fluctuations within *allo*-closite (Figure S8). Most compounds remain well-positioned in the pocket, with RMSD under 3 Å. These extended simulations further underscore the high stability of the identified compounds.

Finally, our simulations revealed evidence of induced fit within the pocket upon ligand binding. The two critical gatekeeper residues, Arg23N-SH2 and Glu176C-SH2 (Figure 5E), initially positioned approximately 17 Å apart, were observed to form a robust salt bridge with a sub-3 Å distance upon full relaxation of the compounds at the end of the 100 ns MD, enhancing pocket stability. Additionally, the polar segments of certain compounds were occasionally seen approaching SHP099 in the *allo*-site-1 pocket, inducing a significant rotation of the 1,4-dimethylpiperidin-4-amine moiety in SHP099. This observation suggests the potential for crosstalk between the *allo*-closite and *allo*-site-1 pockets, possibly resulting in increased SHP2 stability through a dual cooperativity mechanism. Figure 5F depicts the 2D structures of select hit compounds that were consistently found to target the closed state of the SHP2 protein.

## 3. Computational Details

### 3.1. Multi-Tier Molecular Docking

Given several closed states for SHP2 sharing similar structures with mutual RMSD less than 1 Å, we went ahead to choose different receptors at different stages in our docking screening, including *wt*SHP2-ub (closed-conformation, PDB ID: 2SHP [10]) initially, and *wt*SHP2-b (closed-conformation, PDB ID: 5EHR [8]) and *mt*SHP2b (closed-conformation, PDB ID: 6CRG [11]) simultaneously at the second stage.

Prior to initiating our screening approach, we first utilized RDKit (released 2023.03.1), an open-source cheminformatics software [34,35], to refine the 0.26 million compounds from the NCI Open Database. Utilizing a customized Python script, we pre-screened compounds by stripping metal-containing compounds, acknowledging their potential off-target interactions and toxicity risks. We also set a molecular weight threshold of 700 Daltons, emphasizing the importance of solubility and permeability in drug design. Additionally, compounds with an absolute net charge of more than 1 a.u. are discarded to discourage artificially strong electrostatic interactions in docking and to ensure targeted binding efficacy and seamless membrane passage.

We begin with Tier I screening which includes docking of the pre-screened NCI compounds targeting the SHP2 basal state without the SHP099 inhibitor, i.e., *wt*SHP2-ub. The most dominant *wt*SHP2-ub MD solution conformation extracted from the k-mean clustering analysis was utilized as a receptor structure for docking purposes [36,37]. ADFR suite [38] and Meeko python package were used to prepare the protein and ligand structures. AutoDock Vina v1.2.0 [39,40,41] was used in the docking runs. Details of docking setups can be found in Supplementary Table S4. Subsequently, after Tier I screening, we ranked compounds based on theoretical binding affinity (*t-*BA) scores and chose the top 2000 hit compounds for Tier II screening.

In Tier II screening, the top 2000 hits were further docked targeting SHP2 closed states with the SHP099 inhibitor, i.e., *wt*SHP2-b and *mt*SHP2-b. Again, the most dominant MD solution conformation extracted from the k-mean clustering analysis for each complex was utilized as the receptor structure for docking purposes. Subsequently, compounds were ranked based on *t-*BA-scores (Supplementary Table S2A,B).

For consensus docking results, we implemented two distinct selection criteria. The first criterion (*t-*BA) relies on *t-*BA-scores to prioritize compounds. Here we sorted compounds based on *t-*BA-scores for both receptors and then selected common compounds in the top 20 lists. This leads to six common top-hit compounds. We continued by selecting common compounds in the top 40 lists that are not already selected in the top 20 lists. This leads to 11 additional common top-hit compounds. We continued this way for the top 60, top 80, and top 100 lists, leading to a total of 73 consensus top hits (Supplementary Table S3). We further visualized these top hits and selected 18 potential compounds for further analysis (Table 1). In the *h-*VI criterion, we examined the spatial orientation of compounds within the *allo-*closite pocket and their interactions with residues from all three domains of SHP2. We not only visualized the top 2000 compounds, but also lower-ranked compounds. Finally, we found a total of 21 potential compounds (Table 2).

### 3.2. MD Simulations

Four crystal structures were adopted for MD simulations, including ligand-free wild type (PDB ID: 2SHP [10]) and mutant proteins (PDB ID: 6CRF [11]), and ligand-bound wild type (PDBID: 5EHR [8]) and mutant proteins (PDB ID: 6CRG [11]). The missing loops of all the crystal structures were modeled using Modeller in Chimera [42,43,44,45]. The ff19SB force field was used to model proteins [46] and the TIP3P model [47] was used for water. The Amber-compatible GAFF2 force field parameters [48] for all ligands were generated with the Antechamber program of the AmberTools software suite version 2023 [37]. All systems were solvated in a truncated octahedral box with a buffer of 8.0 Å and a NaCl concentration of 150 mM in the LEaP program of AmberTools 2023 [37]. A 9.0 Å cutoff was applied for the short-range electrostatic and nonbonded interactions. The long-range electrostatics was handled with the PME approach with default settings [49]. All bonds to the hydrogen atoms are constrained with the SHAKE algorithm [50,51] so that the leap-frog method [52] with a 2 fs time step could be used for time integration. All simulations were conducted with the PMEMD program for either the CPU or the GPU platform from the Amber software suite version 2023 [36,53].

For each simulation, the initial minimization involved 2000 steps via the steepest descent method, followed by an equal number of steps using the conjugate gradient technique. During minimization, all protein and ligand heavy atoms were restrained with a force constant of 10 kcal/mol-Å while hydrogen atoms and the solvents, including both water molecules and ions, are allowed to relax. Next, a heating step over 200 ps was conducted, starting from 0 K to 298 K with the Langevin thermostat [54] with a collision frequency of 1.0 per ps. Here the constant volume ensemble was used. Following the heating phase, an equilibration phase was undertaken for 5 ns under the NPT ensemble with the Berendsen barostat with default parameters [55], with the restraining force constant reduced to 5 kcal/mol-Å. The equilibration process was then repeated without any restraint. This was followed by the final production run up to 500 ns in the NVT ensemble in the Berendsen thermostat [55]. MD simulations were repeated up to three times to collect sufficient amount of data for conformational analysis. All MD trajectory analysis was conducted with the CPPTRAJ program [56]. Specifically, the k-means clustering method was used to extract dominant conformations from all trajectories and to detect conformational changes. Clustering number was set to 10. The heavy atom (up to side-chain CB atoms) RMSD was used as the distance metric.

## 4. Conclusions and Future Directions

In this study, molecular dynamics (MD) simulations were utilized to elucidate the structural and dynamic properties of SHP2 in both the wild-type (*wt*SHP2, closed state) and the E76K mutant (*mt*SHP2, open state) conformations, examining monomeric and dimeric forms as depicted in crystal structures. A comprehensive dataset was amassed from 28.6-µs MD simulations in an explicit solvent model across various systems.

Our findings reveal that each domain of *wt*SHP2 retains substantial stability, whether in monomer or dimer configuration. This stability is postulated to derive from the dense organization of *wt*SHP2, where domains are tightly packed against each other. Transitioning to *mt*SHP2, the N-SH2 and PTP domains maintain stability comparable to *wt*SHP2. However, the C-SH2 domain demonstrates increased flexibility, consistent with its less defined electron density in the crystal structure, suggesting looser packing both intramolecularly and intermolecularly. Notably, the crystal packing within the unit cell is significantly different between *wt*SHP2 and *mt*SHP2. In *wt*SHP2, our MD simulations of solvated monomers/dimers correspond well with the crystal structure. In contrast, the *mt*SHP2 presents a more complex packing, which may account for the disparities in domain stability observed in our simulations.

Building on these structural insights, our simulations highlight the impacts of the E76K mutation on the second allosteric binding site, where we observed a reduced binding efficacy due to the site’s flat topology, which likely leads to increased inhibitor dissociation. A pivotal aspect of our research was the identification of a new secondary binding pocket, with the hope for robust and comprehensive inhibition of SHP2, especially in its mutant form. This *allo*-closite pocket, situated at the interfaces of the N-SH2, C-SH2, and PTP domains and remote from the SHP099 binding site, exhibited enhanced stability upon SHP099 binding in comparison to the apo form of *wt*SHP2, signifying the pocket’s potential as a selective target that operates via a conformation-selection mechanism.

To further substantiate our findings, we conducted a virtual screening of the predicted pocket against the NCI cancer-specific small molecule library, implementing a three-stage screening protocol. The screening process yielded 39 promising compounds, identified through a consensus selection method complemented by human visualization after the initial screening phases. We then performed 100 ns MD simulations to weed out weak binders among the selected compounds within the *allo*-closite pocket. From these simulations, 11 compounds emerged as highly stable, signifying their potential as hit compounds.

Looking forward, the intricate structural understanding of SHP2 and its mutants obtained from this study opens new avenues for further research into novel therapeutic interventions. Our findings underscore the potential of allosteric inhibitors, particularly highlighting the importance of prolonged target binding for effective SHP2 suppression. While our work is purely computational and requires experimental validation, the identification and virtual screening validation of a novel allosteric pocket provides a compelling hypothesis for future investigation. We encourage experimentalists within the scientific community to explore this avenue further, following established strategies [11,12]. Continued research in this direction promises to refine drug design strategies, potentially leading to more effective treatments for SHP2-associated cancers.

## Figures and Tables

**Table 1:** The top 18 compounds selected from 73 common top-hit compounds at the end of Tier II screening (affinity in kcal/mol).

Label	*wt*SHP2 + SHP099		*mt*SHP2 + SHP099	
	Molecule-ID	Affinity	Molecule-ID	Affinity
C1	NSC-103858	−7.4	NSC-103858	−7.5
C2	NSC-106445	−7.7	NSC-106445	−7.9
C3	NSC-118695	−7.5	NSC-118695	−7.5
C4	NSC-121342	−7.3	NSC-121342	−7.2
C5	NSC-121974	−8.4	NSC-121974	−7.6
C6	NSC-210399	−7.5	NSC-210399	−7.2
C7	NSC-252124	−7.4	NSC-252124	−7.6
C8	NSC-261054	−7.4	NSC-261054	−7.3
C9	NSC-30502	−8.1	NSC-30502	−7.6
C10	NSC-39355	−7.3	NSC-39355	−7.4
C11	NSC-39913	−7.5	NSC-39913	−7.4
C12	NSC-39917	−7.5	NSC-39917	−7.7
C13	NSC-60678	−8.4	NSC-60678	−8.3
C14	NSC-67586	−7.4	NSC-67586	−7.4
C15	NSC-163300	−8.6	NSC-163300	−7.9
C16	NSC-23127	−6.2	NSC-23127	−7.5
C17	NSC-250352	−8.2	NSC-250352	−7.4
C18	NSC-74702	−7.8	NSC-74702	−7.9

**Table 2:** Manually selected top 21 compounds from the Tier I and Tier II screening (affinity in kcal/mol).

Label	*wt*SHP2 + SHP099		*mt*SHP2 + SHP099	
	Molecule-ID	Affinity	Molecule-ID	Affinity
C1	NSC-14757	−7.9	NSC-14757	−8.1
C2	NSC-153191	−8.1	NSC-153191	−7.6
C3	NSC-211584	−7.3	NSC-211584	−7.1
C4	NSC-299137	−7.8	NSC-299137	−7.7
C5	NSC-371876	−7.4	NSC-371876	−7.3
C6	NSC-380323	−7.6	NSC-380323	−7.5
C7	NSC-39909	−7.5	NSC-39909	−7.4
C8	NSC-39918	−9.7	NSC-39918	−7.5
C9	NSC-618161	−7.6	NSC-618161	−7.5
C10	NSC-637201	−7.8	NSC-637201	−8.1
C11	NSC-649243	−7.2	NSC-649243	−7.2
C12	NSC-649245	−7.7	NSC-649245	−7.5
C13	NSC-665127	−7.9	NSC-665127	−7.6
C14	NSC-747599	−7.3	NSC-747599	−7.2
C15	NSC-85195	−7.3	NSC-85195	−7.2
C16	NSC-103851	−7.4	NSC-103851	−7.4
C17	NSC-252174	−8.9	NSC-252174	−7.8
C18	NSC-400376	−7.7	NSC-400376	−7.5
C19	NSC-53298	−7.1	NSC-53298	−7.7
C20	NSC-63675	−7.1	NSC-63675	−7.2
C21	NSC-74671	−7.1	NSC-74671	−7.3

## Data Availability

Simulation data available from authors upon request.

## Supplementary Materials

The supporting information can be downloaded at: https://www.mdpi.com/article/10.3390/ijms25116201/s1.

## References

[1] Chan G., Kalaitzidis D., Neel B.G. (2008). The tyrosine phosphatase Shp2 (PTPN11) in cancer. Cancer Metastasis Rev..

[2] Li J., Jie H.B., Lei Y., Gildener-Leapman N., Trivedi S., Green T., Kane L.P., Ferris R.L. (2015). PD-1/SHP-2 inhibits Tc1/Th1 phenotypic responses and the activation of T cells in the tumor microenvironment. Cancer Res..

[3] Noguchi T., Matozaki T., Horita K., Fujioka Y., Kasuga M. (1994). Role of SH-PTP2, a protein-tyrosine phosphatase with Src homology 2 domains, in insulin-stimulated Ras activation. Mol. Cell. Biol..

[4] Zhang J., Zhang F., Niu R. (2015). Functions of Shp2 in cancer. J. Cell. Mol. Med..

[5] Tartaglia M., Mehler E.L., Goldberg R., Zampino G., Brunner H.G., Kremer H., van der Burgt I., Crosby A.H., Ion A., Jeffery S. (2001). Mutations in PTPN11, encoding the protein tyrosine phosphatase SHP-2, cause Noonan syndrome. Nat. Genet..

[6] Tartaglia M., Niemeyer C.M., Fragale A., Song X., Buechner J., Jung A., Hählen K., Hasle H., Licht J.D., Gelb B.D. (2003). Somatic mutations in PTPN11 in juvenile myelomonocytic leukemia, myelodysplastic syndromes and acute myeloid leukemia. Nat. Genet..

[7] Mohi M.G., Williams I.R., Dearolf C.R., Chan G., Kutok J.L., Cohen S., Morgan K., Boulton C., Shigematsu H., Keilhack H. (2005). Prognostic, therapeutic, and mechanistic implications of a mouse model of leukemia evoked by Shp2 (PTPN11) mutations. Cancer Cell.

[8] Chen Y.N., LaMarche M.J., Chan H.M., Fekkes P., Garcia-Fortanet J., Acker M.G., Antonakos B., Chen C.H., Chen Z., Cooke V.G. (2016). Allosteric inhibition of SHP2 phosphatase inhibits cancers driven by receptor tyrosine kinases. Nature.

[9] Prahallad A., Heynen G.J., Germano G., Willems S.M., Evers B., Vecchione L., Gambino V., Lieftink C., Beijersbergen R.L., Di Nicolantonio F. (2015). PTPN11 Is a Central Node in Intrinsic and Acquired Resistance to Targeted Cancer Drugs. Cell Rep..

[10] Hof P., Pluskey S., Dhe-Paganon S., Eck M.J., Shoelson S.E. (1998). Crystal structure of the tyrosine phosphatase SHP-2. Cell.

[11] LaRochelle J.R., Fodor M., Vemulapalli V., Mohseni M., Wang P., Stams T., LaMarche M.J., Chopra R., Acker M.G., Blacklow S.C. (2018). Structural reorganization of SHP2 by oncogenic mutations and implications for oncoprotein resistance to allosteric inhibition. Nat. Commun..

[12] Pádua R.A.P., Sun Y., Marko I., Pitsawong W., Stiller J.B., Otten R., Kern D. (2018). Mechanism of activating mutations and allosteric drug inhibition of the phosphatase SHP2. Nat. Commun..

[13] Motlagh H.N., Wrabl J.O., Li J., Hilser V.J. (2014). The ensemble nature of allostery. Nature.

[14] Strotz D., Orts J., Kadavath H., Friedmann M., Ghosh D., Olsson S., Chi C.N., Pokharna A., Güntert P., Vögeli B. (2020). Protein Allostery at Atomic Resolution. Angew. Chem. Int. Ed..

[15] Anselmi M., Hub J.S. (2020). An allosteric interaction controls the activation mechanism of SHP2 tyrosine phosphatase. Sci. Rep..

[16] Hou Y., Lu X., Xu Z., Qu J., Huang J. (2023). How a single mutation alters the protein structure: A simulation investigation on protein tyrosine phosphatase SHP2. RSC Adv..

[17] Gampp O., Kadavath H., Riek R. (2024). NMR tools to detect protein allostery. Curr. Opin. Struct. Biol..

[18] Calligari P., Santucci V., Stella L., Bocchinfuso G. (2021). Discriminating between competing models for the allosteric regulation of oncogenic phosphatase SHP2 by characterizing its active state. Comput. Struct. Biotechnol. J..

[19] Tao Y., Xie J., Zhong Q., Wang Y., Zhang S., Luo F., Wen F., Xie J., Zhao J., Sun X. (2021). A novel partially open state of SHP2 points to a “multiple gear” regulation mechanism. J. Biol. Chem..

[20] Anselmi M., Hub J.S. (2023). Atomistic ensemble of active SHP2 phosphatase. Commun. Biol..

[21] Garcia Fortanet J., Chen C.H., Chen Y.N., Chen Z., Deng Z., Firestone B., Fekkes P., Fodor M., Fortin P.D., Fridrich C. (2016). Allosteric Inhibition of SHP2: Identification of a Potent, Selective, and Orally Efficacious Phosphatase Inhibitor. J. Med. Chem..

[22] LaRochelle J.R., Fodor M., Ellegast J.M., Liu X., Vemulapalli V., Mohseni M., Stams T., Buhrlage S.J., Stegmaier K., LaMarche M.J. (2017). Identification of an allosteric benzothiazolopyrimidone inhibitor of the oncogenic protein tyrosine phosphatase SHP2. Bioorg. Med. Chem..

[23] LaMarche M.J., Acker M., Argintaru A., Bauer D., Boisclair J., Chan H., Chen C.H., Chen Y.N., Chen Z., Deng Z. (2020). Identification of TNO155, an Allosteric SHP2 Inhibitor for the Treatment of Cancer. J. Med. Chem..

[24] Ou S., Koczywas M., Ulahannan S., Janne P., Pacheco J., Burris H., McCoach C., Wang J., Gordon M., Haura E. (2020). A12 the SHP2 inhibitor RMC-4630 in patients with KRAS-mutant non-small cell lung cancer: Preliminary evaluation of a first-in-man phase 1 clinical trial. J. Thorac. Oncol..

[25] Vemulapalli V., Donovan K.A., Seegar T.C.M., Rogers J.M., Bae M., Lumpkin R.J., Cao R., Henke M.T., Ray S.S., Fischer E.S. (2021). Targeted Degradation of the Oncogenic Phosphatase SHP2. Biochemistry.

[26] Han H., Panliang G., Ma C., Di K. (2022). Novel Heterocyclic Derivatives Useful as SHP2 Inhibitors.

[27] Wang M., Li T., Ouyang Z., Tang K., Zhu Y., Song C., Sun H., Yu B., Ji X., Sun Y. (2022). SHP2 allosteric inhibitor TK-453 alleviates psoriasis-like skin inflammation in mice via inhibition of IL-23/Th17 axis. iScience.

[28] Guo M.C., Li Z.K., Gu M.X., Gu J.R., You Q.D., Wang L. (2024). Targeting phosphatases: From molecule design to clinical trials. Eur. J. Med. Chem..

[29] Luo Y.M., Li J., Zong Y.L., Sun M.X., Zheng W., Zhu J.P., Liu L., Liu B. (2023). Discovery of the SHP2 allosteric inhibitor 2-((3R,4R)-4-amino-3-methyl-2-oxa-8-azaspiro[4.5]decan-8-yl)-5-(2,3-dichlorophenyl)-3-methylpyrrolo[2,1-f][1,2,4] triazin-4(3H)-one. J. Enzym. Inhib. Med. Chem..

[30] Luo R.X., Fu W.T., Shao J.J., Ma L., Shuai S.J., Xu Y., Jiang Z., Ye Z.H., Zheng L.L., Zheng L. (2023). Discovery of a potent and selective allosteric inhibitor targeting the SHP2 tunnel site for RTK-driven cancer treatment. Eur. J. Med. Chem..

[31] Ruess D.A., Heynen G.J., Ciecielski K.J., Ai J., Berninger A., Kabacaoglu D., Gorgulu K., Dantes Z., Wormann S.M., Diakopoulos K.N. (2018). Mutant KRAS-driven cancers depend on PTPN11/SHP2 phosphatase. Nat. Med..

[32] Fodor M., Price E., Wang P., Lu H., Argintaru A., Chen Z., Glick M., Hao H.X., Kato M., Koenig R. (2018). Dual Allosteric Inhibition of SHP2 Phosphatase. ACS Chem. Biol..

[33] Ravindranath P.A., Sanner M.F. (2016). AutoSite: An automated approach for pseudo-ligands prediction-from ligand-binding sites identification to predicting key ligand atoms. Bioinformatics.

[34] Petitjean M., Camproux A.-C. (2016). In Silico Medicinal Chemistry: Computational Methods to Support Drug Design. Edited by Nathan Brown. ChemMedChem.

[35] Landrum G. (2016). Rdkit: Open-Source Cheminformatics Software. https://github.com/rdkit/rdkit.

[36] Case D.A., Aktulga H.M., Belfon K., Ben-Shalom I.Y., Berryman J.T., Brozell S.R., Cerutti D.S., Cheatham T.E., Cisneros G.A., Cruzeiro V.W.D. Amber 2023.

[37] Case D.A., Aktulga H.M., Belfon K., Cerutti D.S., Cisneros G.A., Cruzeiro V.W.D., Forouzesh N., Giese T.J., Götz A.W., Gohlke H. (2023). The AmberTools. J. Chem. Inf. Model..

[38] Ravindranath P.A., Forli S., Goodsell D.S., Olson A.J., Sanner M.F. (2015). Advances in Protein-Ligand Docking with Explicitly Specified Binding Site Flexibility. PLoS Comput. Biol..

[39] Forli S., Huey R., Pique M.E., Sanner M.F., Goodsell D.S., Olson A.J. (2016). Computational protein-ligand docking and virtual drug screening with the AutoDock suite. Nat. Protoc..

[40] Eberhardt J., Santos-Martins D., Tillack A.F., Forli S. (2021). AutoDock Vina 1.2.0: New Docking Methods, Expanded Force Field, and Python Bindings. J. Chem. Inf. Model..

[41] Trott O., Olson A.J. (2010). Software News and Update AutoDock Vina: Improving the Speed and Accuracy of Docking with a New Scoring Function, Efficient Optimization, and Multithreading. J. Comput. Chem..

[42] Webb B., Sali A. (2016). Comparative Protein Structure Modeling Using Modeller. Curr. Protoc. Bioinform..

[43] Marti-Renom M.A., Stuart A.C., Fiser A., Sanchez R., Melo F., Sali A. (2000). Comparative protein structure modeling of genes and genomes. Annu. Rev. Biophys. Biomol. Struct..

[44] Sali A., Blundell T.L. (1993). Comparative protein modelling by satisfaction of spatial restraints. J. Mol. Biol..

[45] Fiser A., Do R.K., Sali A. (2000). Modeling of loops in protein structures. Protein Sci..

[46] Tian C., Kasavajhala K., Belfon K.A.A., Raguette L., Huang H., Migues A.N., Bickel J., Wang Y.Z., Pincay J., Wu Q. (2020). ff19SB: Amino-Acid-Specific Protein Backbone Parameters Trained against Quantum Mechanics Energy Surfaces in Solution. J. Chem. Theory Comput..

[47] Jorgensen W.L., Chandrasekhar J., Madura J.D., Impey R.W., Klein M.L. (1983). Comparison of Simple Potential Functions for Simulating Liquid Water. J. Chem. Phys..

[48] Wang J.M., Wolf R.M., Caldwell J.W., Kollman P.A., Case D.A. (2004). Development and testing of a general amber force field. J. Comput. Chem..

[49] Darden T., York D., Pedersen L. (1993). Particle Mesh Ewald—An N.Log(N) Method for Ewald Sums in Large Systems. J. Chem. Phys..

[50] Ryckaert J.P., Ciccotti G., Berendsen H.J.C. (1977). Numerical-Integration of Cartesian Equations of Motion of a System with Constraints—Molecular-Dynamics of N-Alkanes. J. Comput. Phys..

[51] Miyamoto S., Kollman P.A. (1992). Settle—An Analytical Version of the Shake and Rattle Algorithm for Rigid Water Models. J. Comput. Chem..

[52] Michael P., Allen D.J.T. (2017). Computer Simulation of Liquids.

[53] Case D.A., Cheatham T.E., Darden T., Gohlke H., Luo R., Merz K.M., Onufriev A., Simmerling C., Wang B., Woods R.J. (2005). The Amber biomolecular simulation programs. J. Comput. Chem..

[54] Pastor R.W., Brooks B.R., Szabo A. (1988). An Analysis of the Accuracy of Langevin and Molecular-Dynamics Algorithms. Mol. Phys..

[55] Berendsen H.J.C., Postma J.P.M., Vangunsteren W.F., Dinola A., Haak J.R. (1984). Molecular-Dynamics with Coupling to an External Bath. J. Chem. Phys..

[56] Roe D.R., Cheatham T.E. (2013). PTRAJ and CPPTRAJ: Software for Processing and Analysis of Molecular Dynamics Trajectory Data. J. Chem. Theory Comput..

