# Supplementary material for: Targeting SHP2 Cryptic Allosteric Sites for Effective Cancer Therapy
Source: Int J Mol Sci. 2024 Jun 4;25(11):6201. doi: 10.3390/ijms25116201 (PMC11172685; doi:10.3390/ijms25116201)
Supplement: Supplementary file 1 [file ijms-25-06201-s001.zip › ijms-2994082-supplementary.pdf]

Stability of Individual SHP2 Domains in Closed and Open States

N-SH2 Domain. Our analysis, utilizing CA-RMSD of core residues as a reference to initial structures, reveals the N-SH2 domain's consistently high stability across all simulations. This encompasses both monomeric forms—chain A (chA) and chain B (chB)—and the dimeric form (chA+chB) for both wtSHP2 and mtSHP2, as illustrated in **Figure S1A**.

Interestingly, prior NMR studies by Kern et al. emphasized the inherent flexibility of the N-SH2 domain in mtSHP2, a factor that has hindered successful crystallization of the open state of SHP2.[12] Moreover, their comparative [1H-15N]-TROSY-HSQC NMR spectra revealed significant conformational differences between wild-type (wtSHP2) and mutant (mtSHP2) SHP2. However, our findings on intra-domain stability are not inconsistent with their results. While Kern et al. highlighted the global structural discrepancies between wtSHP2 and the E76K mutant, focusing on the relative positions of domains and their impact on crystallization, our analysis complements their work by examining the fluctuations within individual domains to further elucidate the E76K mutation's effect on stability. We also report substantial inter-domain dynamics later in our study. Additionally, our crystallographic comparison of wtSHP2 and mtSHP2 supports the computational observation of a 'stable' N-SH2 domain, demonstrating a minimal core-RMSD of 0.43 Å for this domain.

Figure S1A portrays the N-SH2 domain's superior stability in wtSHP2, closely mirroring the initial crystal structure throughout simulations. This is evidenced by a core-RMSD generally below 1.00 Å. In mtSHP2, the N-SH2 domain shows more significant deviation from the original crystal structures, with core-RMSDs often approaching 2.00 Å. A k-means clustering analysis (**Figure S1D**) supports the N-SH2 domain's robust secondary structure stability in wtSHP2, whereas mtSHP2 shows a propensity for disordered loops, likely due to weakened interactions with the PTP domain. The crystal structure of mtSHP2, with its additional unresolved residues, further substantiates the stability discrepancies observed.

C-SH2 Domain. The dynamic behavior of the C-SH2 domain markedly varies between wtSHP2 and mtSHP2 (**Figure S1B**). In wtSHP2, MD simulations consistently suggest high stability for the C-SH2 domain, supporting its proposed role in bolstering the N-SH2 domain's stability, which is crucial for SHP2's full functional range. On the contrary, mtSHP2 simulations indicate a dynamically active C-SH2 domain, with increased core-RMSD values signifying notable deviations from the initial structure (**Figure S1E**). This correlates with the unresolved interface residues with N-SH2 in the mtSHP2 crystal structure.[11] The considerable movement in chB simulations corresponds to the impaired structural integrity of chB in mtSHP2. Notably, the E76K mutation's indirect effects on C-SH2 underscore the importance of domain interfaces in influencing stability.

PTP Domain. The PTP domain, in both wtSHP2 and mtSHP2, exhibits consistent stability in our simulations (**Figure S1C/F**). Interestingly, the core CA-RMSD of mtSHP2's PTP domain is lower than that of wtSHP2, suggesting enhanced stability possibly due to the oncogenic E76K mutation's induced conformational shifts, which weaken a crucial PTP-N-SH2 interaction and cause SH2 domain rotation.[11]

In summary, each domain within wtSHP2, whether in monomeric or dimeric form, demonstrates significant stability, potentially due to the compact organization of the protein, where domains are tightly packed together. Conversely, in mtSHP2, while the N-SH2 and PTP domains maintain stability comparable to wtSHP2, the C-SH2 domain exhibits increased flexibility. This is consistent with its lower certainty in the crystal structure, due to its relatively looser packing environment both intermolecularly and intramolecular (i.e. from crystal packing) as discussed below.

SHP2 Open and Closed States Exhibit Differing Crystal Packing

Our MD simulations suggest that the dynamics of individual SHP2 domains are highly responsive to their environmental context. As introduced earlier (refer to **Figure 1B/C**), the domain configurations of wtSHP2 and mtSHP2 are markedly dissimilar. Beyond mere domain arrangement, the impact of crystal packing is particularly noteworthy. Although both wtSHP2 and mtSHP2 crystal structures harbor two monomers within their asymmetric units, their overall crystal packing within the unit cell is distinct.

For wtSHP2's asymmetric unit, the PTP domains of two chains engage in a "head-to-head" symmetrical association (as shown in Supplementary **Figure S2A1/B1**). Within the unit cell, four monomers are organized linearly in a relatively straightforward packing pattern (Supplementary **Figure S2C1**). This configuration is mirrored in our MD simulations of solvated monomers/dimers. Conversely, mtSHP2's asymmetric unit is characterized by a unique "head-to-tail" arrangement of the two chains (Supplementary **Figure S2A2/B2**). This setup leads to eight monomers arrayed within the unit cell in a more complex packing formation (Supplementary **Figure S2C2**), resulting in solvated monomer/dimer MD simulations that are less representative of this intricate crystal packing.

The observed variability in the dynamics of the C-SH2 domain in mtSHP2 may be due, in part, to the absence of crystal packing influences in MD simulations. In contrast, the enhanced stability of the C-SH2 domain in wtSHP2 simulations could arise from solvation effects that better emulate the crystal environment of wtSHP2. It is noteworthy that the symmetric crystal packing observed in wtSHP2 is more commonly encountered across available SHP2 structures than that of mtSHP2, as demonstrated by structures with PDB codes such as 5EHP, 6CRG, 6MDA, 6MDB, and others (refer to Supplementary **Figure S2D**). This frequent occurrence could shed light on the difficulties associated with crystallizing the E76K mutant of SHP2. Understanding these dynamics in the solution phase becomes particularly significant when considering the limited number of open-state structures available.

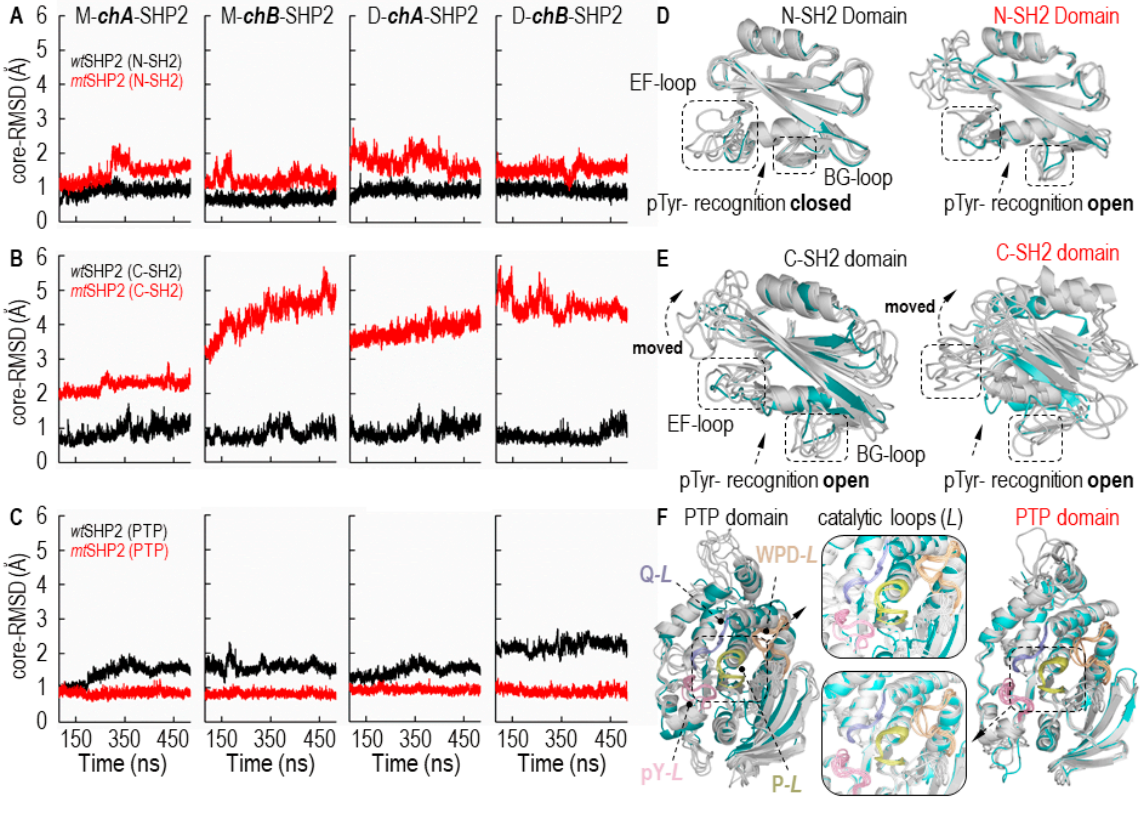

Figure S1. Domain Stability Comparison of SHP2 Across Different States. Panels (**A-C**) present RMSD plots for the N-SH2, C-SH2, and PTP domains, contrasting the closed (black) and open (red) states. The left two columns show monomeric forms (M-chA or M-chB), and the right two columns show dimeric forms (D-chA or D-chB). Panels (**D-F**) depict the top-5 superimposed clusters (grey) against the reference crystal structure (cyan). Notably, the BG-loop and EF-loop (phosphotyrosine-recognition loops) maintain stability solely in the wtSHP2 N-SH2 closed state. Conversely, the C-SH2 domain, characterized by its flexibility, exhibits significant fluctuations and an upward shift in these loops, indicative of the recognition loops' opening. The domain structure cartoons to the left represent the wild-type (black), and those to the right depict the mutant (red).

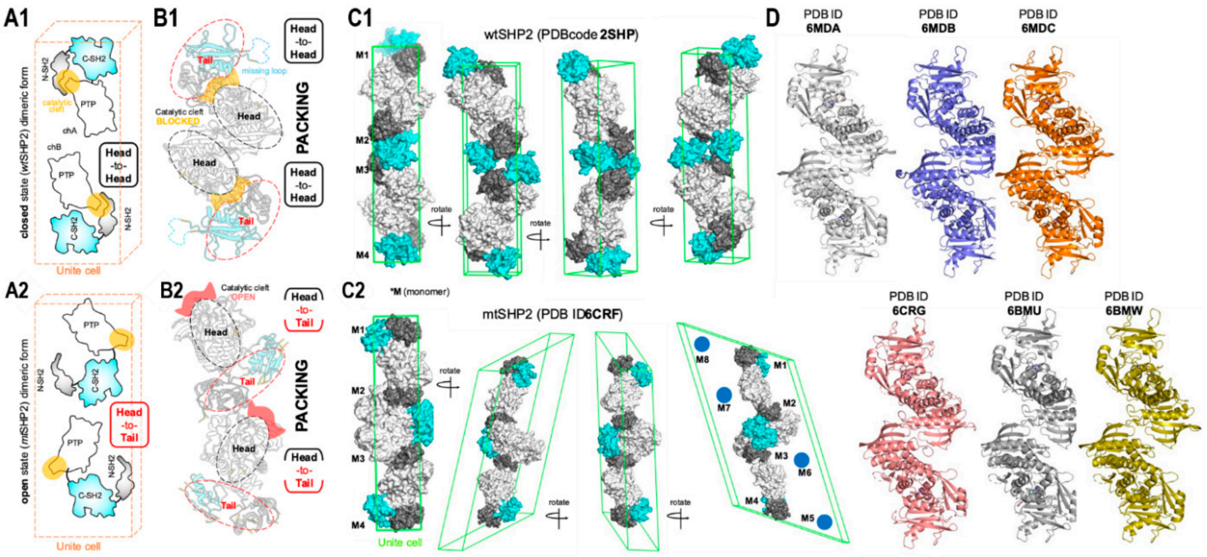

Figure S2. Crystal Packing Patterns of SHP2 in Closed and Open States. (**A1/A2**) Display the unit cell's cartoon representation for the closed state of SHP2 (wtSHP2), demonstrating the "head-to-head" conformation and symmetrical arrangement characteristic of the closed state, contrasted with the open state of SHP2 (mtSHP2), showcasing the "head-to-tail" conformation and asymmetrical arrangement typical of the open state. (**B1/B2**) Depict the three-dimensional structure of the wtSHP2/mtSHP2 dimer, with the 'head' (PTP domain) and 'tail' (SH2 domain) regions labeled to highlight the interaction interfaces, including the substrate entrance regions. (**C1/C2**) Present rotated views of the wtSHP2/mtSHP2 unit cells (rotated by 45 degrees along the x-axis). The N-SH2 and C-SH2 domains are colored in grey and cyan, respectively, with the PTP domain in snow color. The wtSHP2 unit cell houses four monomers (M1-M4), while the mtSHP2 unit cell is structured to accommodate eight monomers. For visual clarity, only monomers M1-M4 are displayed, with the locations of monomers M5-M8 indicated but not rendered. (**D**) Illustrates the protein structures from various SHP2 crystals, all exhibiting the "head-to-head" symmetry in their crystal packing, with corresponding PDB IDs provided for reference.

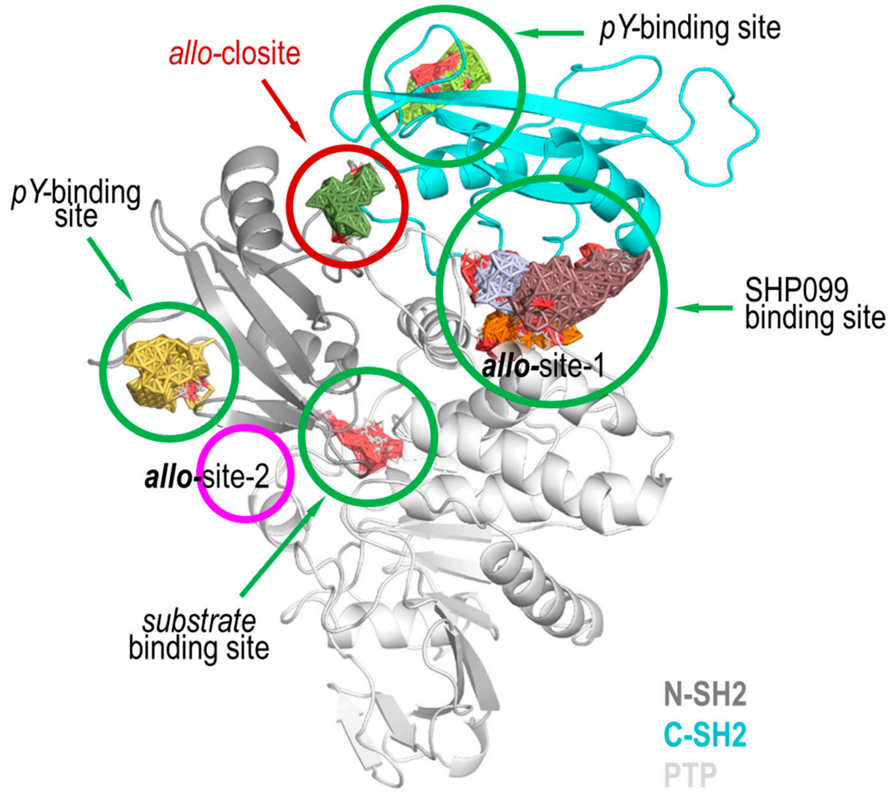

Figure S3. Identified binding sites include multiple known pockets (green circles: *allo*-site-1, pY-binding sites, and substrate binding site) and one unknown pocket (red circle). The unknown pocket is termed *allo*-closite pocket in this study. The analysis does not recognize previously predicted *allo*-site-2 (pink circle) for second inhibitor binding.

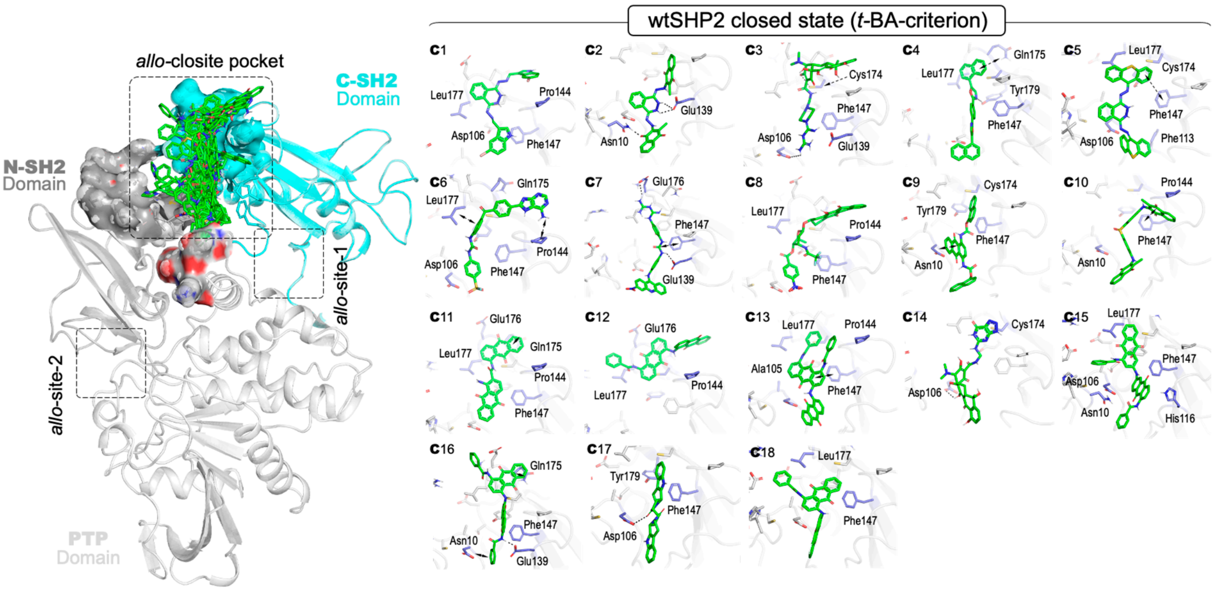

Figure S4. Docked poses of 18 selected common hits compounds based on tBA-score criteria. The receptor used here is the extracted centroid structure of the most dominant cluster of wtSHP2 in the presence of SHP099. Our top selected compounds are colored green and *allo*-closite residues are colored white (stick).

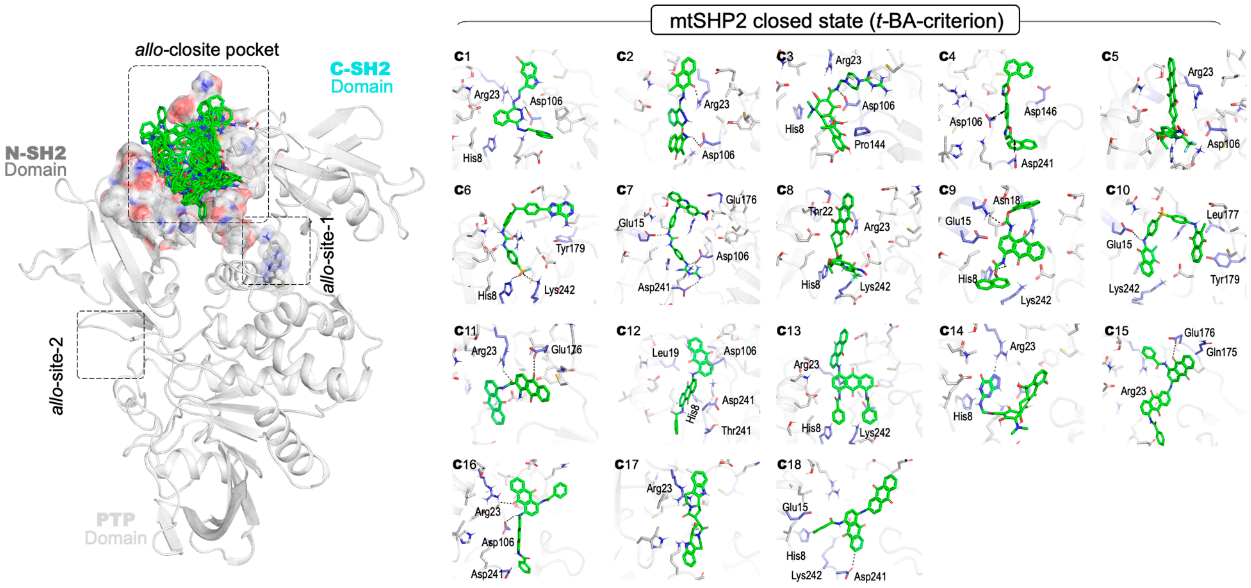

Figure S5. Docked poses of top 18 selected common hits compounds based on tBA-score criteria. The receptor used here is the centroid structure of the most dominant cluster of mtSHP2 in the presence of SHP099. Our top selected compounds are colored green and *allo*-closite residues are colored white (stick).

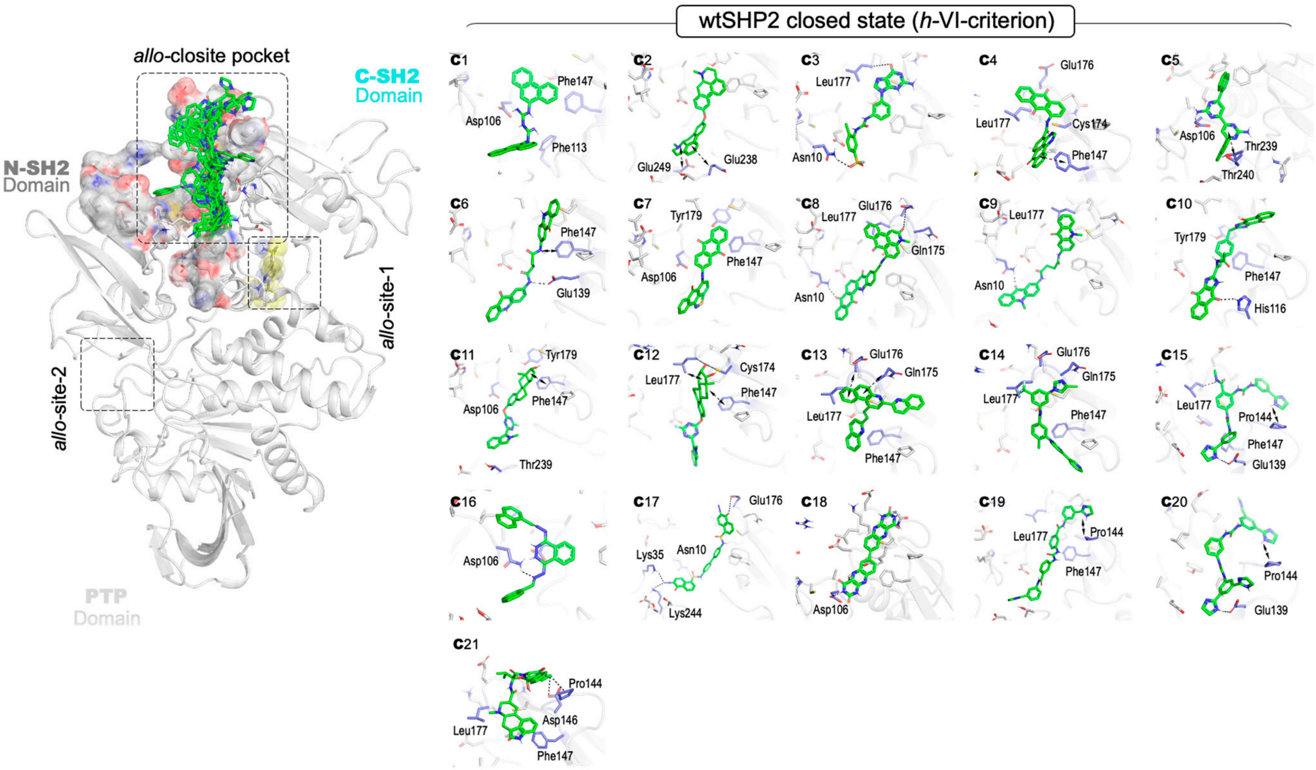

Figure S6. Docked poses for 21 common hits compounds selected based on h-VI based criteria. The receptor used here is the centroid structure of the most dominant cluster of wtSHP2 in the presence of SHP099. Our top selected compounds are colored green and *allo*-closite residues are colored white (stick).

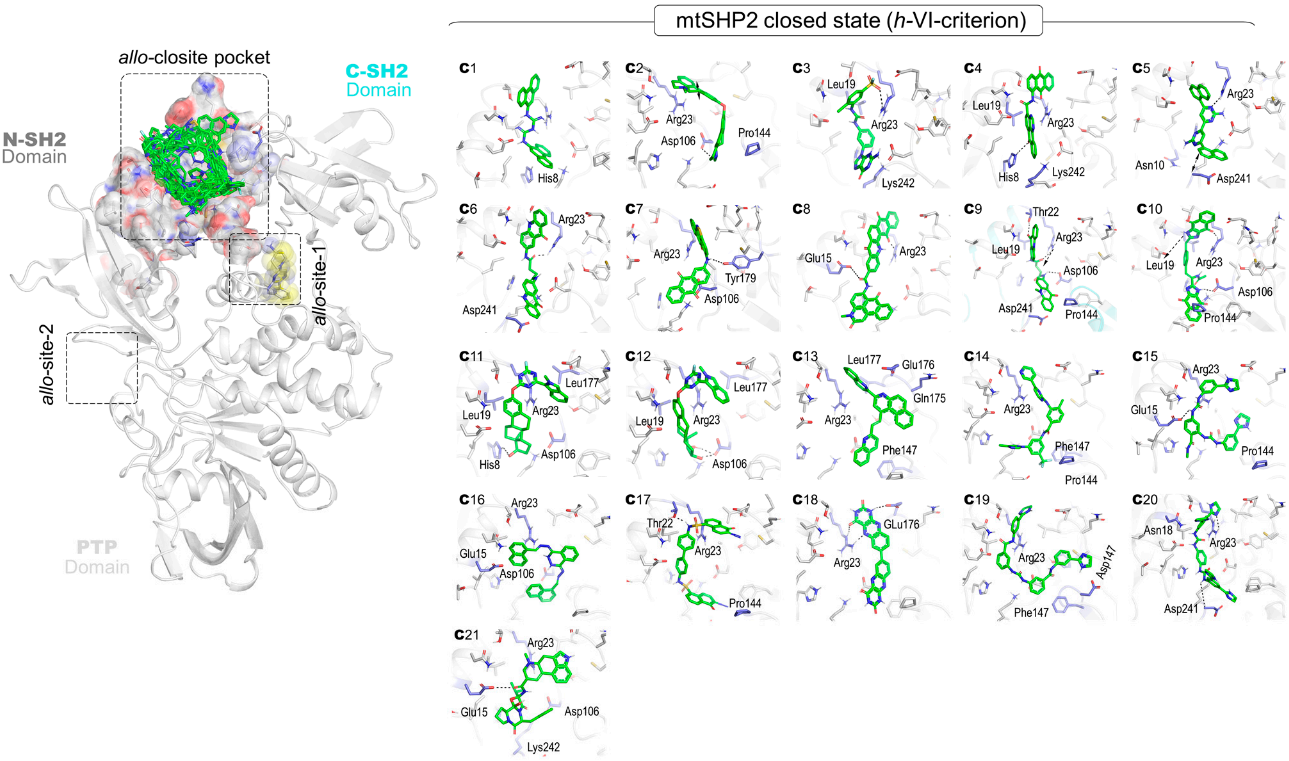

Figure S7. Docked poses for top 21 common hits compounds selected based on h-VI based criteria. The receptor used here is the centroid structure of the most dominant cluster of mtSHP2 in the presence of SHP099. Our top selected compounds are colored green and *allo*-closite residues are colored white (stick).

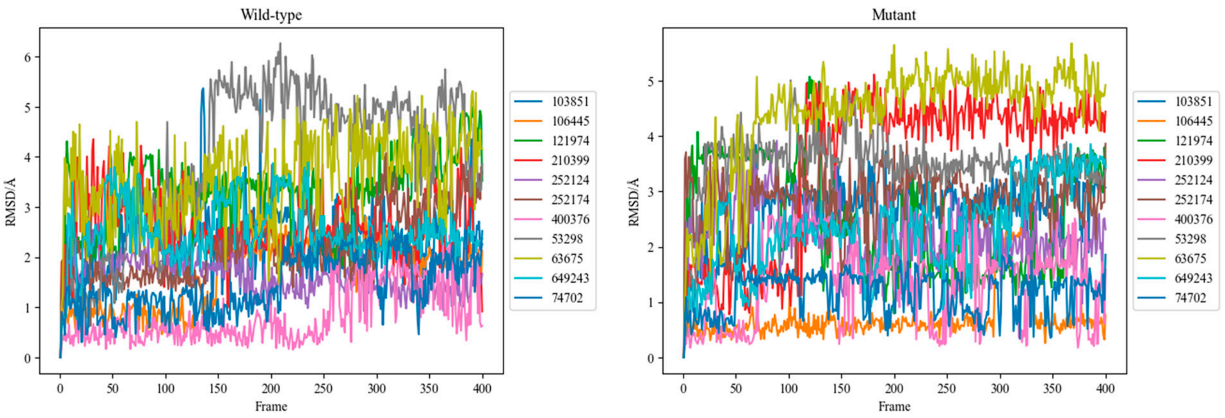

Figure S8. RMSD analysis of extended 400ns MD simulations of 11 selected ligands.

Table S1. Details of MD simulations.

**No of replicas**	**Individual System Labels**		**chain** (ch)	**Time per replica** (ns)
	wtSHP2–*ub*	SHP099	chA/chB	500/500
4x3	wtSHP2–*b*	SHP099	chA	500
	wtSHP2–*ub*	SHP099	chA+chB	500
	mtSHP2–*ub*	SHP099	chA/chB	500/500
4x3	mtSHP2–*b*	SHP099	chA	500
	mtSHP2–*ub*	SHP099	chA+chB	500
	**MD-** *t*-BA-criterion			
	wtSHP2–b	SHP099 + NSC-103858		
	wtSHP2–b	SHP099 + NSC-118695		
	wtSHP2–b	SHP099 + NSC-121342		
	wtSHP2–b	SHP099 + NSC-261054		
	wtSHP2–b	SHP099 + NSC-30502		
	wtSHP2–b	SHP099 + NSC-39355		
13x1	wtSHP2–b	SHP099 + NSC-39913	chA	100
	wtSHP2–b	SHP099 + NSC-39917		
	wtSHP2–b	SHP099 + NSC-60678		
	wtSHP2–b	SHP099 + NSC-67586		
	wtSHP2–b	SHP099 + NSC-163300		
	wtSHP2–b	SHP099 + NSC-23127		
	wtSHP2–b	SHP099 + NSC-250352		
	wtSHP2–b	SHP099 + NSC-252124		
	wtSHP2–b	SHP099 + NSC-74702		
5x1	wtSHP2–b	SHP099 + NSC-106445	chA	500
	wtSHP2–b	SHP099 + NSC-121974		
	wtSHP2–b	SHP099 + NSC-210399		
	mtSHP2–b	SHP099 + NSC-103858		
	mtSHP2–b	SHP099 + NSC-118695		
	mtSHP2–b	SHP099 + NSC-121342		
	mtSHP2–b	SHP099 + NSC-261054		
	mtSHP2–b	SHP099 + NSC-30502		
	mtSHP2–b	SHP099 + NSC-39355		
13x1	mtSHP2–b	SHP099 + NSC-39913	chA	100
	mtSHP2–b	SHP099 + NSC-39917		
	mtSHP2–b	SHP099 + NSC-60678		
	mtSHP2–b	SHP099 + NSC-67586		
	mtSHP2–b	SHP099 + NSC-163300		
	mtSHP2–b	SHP099 + NSC-23127		
	mtSHP2–b	SHP099 + NSC-250352		

	mtSHP2–b	SHP099 + NSC-252124		
	mtSHP2–b	SHP099 + NSC-74702		
5x1	mtSHP2–b	SHP099 + NSC-106445	chA	500
	mtSHP2–b	SHP099 + NSC-121974		
	mtSHP2–b	SHP099 + NSC-210399		
	**MD** *h*-VI-criterion		chA	100
	wtSHP2–b	SHP099 + NSC-14757		
	wtSHP2–b	SHP099 + NSC-153191		
	wtSHP2–b	SHP099 + NSC-211584		
	wtSHP2–b	SHP099 + NSC-299137		
	wtSHP2–b	SHP099 + NSC-371876		
	wtSHP2–b	SHP099 + NSC-380323		
	wtSHP2–b	SHP099 + NSC-39909		
	wtSHP2–b	SHP099 + NSC-39918	chA	100
	wtSHP2–b	SHP099 + NSC-618161		
	wtSHP2–b	SHP099 + NSC-637201		
	wtSHP2–b	SHP099 + NSC-649245		
	wtSHP2–b	SHP099 + NSC-665127		
15x1	wtSHP2–b	SHP099 + NSC-747599		
	wtSHP2–b	SHP099 + NSC-85195		
	wtSHP2–b	SHP099 + NSC-74671		
	wtSHP2–b	SHP099 + NSC-103851		
	wtSHP2–b	SHP099 + NSC-63675		
6x1	wtSHP2–b	SHP099 + NSC-53298	chA	500
	wtSHP2–b	SHP099 + NSC-400376		
	wtSHP2–b	SHP099 + NSC-649243		
	wtSHP2–b	SHP099 + NSC-252174		
	mtSHP2–b	SHP099 + NSC-14757		
	mtSHP2–b	SHP099 + NSC-153191		
	mtSHP2–b	SHP099 + NSC-211584		
	mtSHP2–b	SHP099 + NSC-299137		
	mtSHP2–b	SHP099 + NSC-371876		
	mtSHP2–b	SHP099 + NSC-380323		
	mtSHP2–b	SHP099 + NSC-39909		
	mtSHP2–b	SHP099 + NSC-39918	chA	100
	mtSHP2–b	SHP099 + NSC-618161		
	mtSHP2–b	SHP099 + NSC-637201		
	mtSHP2–b	SHP099 + NSC-649245		
	mtSHP2–b	SHP099 + NSC-665127		
15x1	mtSHP2–b	SHP099 + NSC-747599		
	mtSHP2–b	SHP099 + NSC-85195		
	mtSHP2–b	SHP099 + NSC-74671		
	mtSHP2–b	SHP099 + NSC-103851		
	mtSHP2–b	SHP099 + NSC-63675		
6x1	mtSHP2–b	SHP099 + NSC-53298	chA	500
	mtSHP2–b	SHP099 + NSC-400376		
	mtSHP2–b	SHP099 + NSC-649243		
	mtSHP2–b	SHP099 + NSC-252174		

Table S2-A. Results of Tier-II docking: Top 2000 compounds against the SHP099-bound SHP2 wild type MD structure. These compounds were selected based on binding affinity scores from Tier-I docking (unit: kcal/mol).

Molecule-ID	Affinity	Molecule-ID	Affinity	Molecule-ID	Affinity	Molecule-ID	Affinity
NSC-117812	-12.5	NSC-67693	-8.9	NSC-152144	-8.6	NSC-142016	-8.4
NSC-139812	-12	NSC-101551	-8.9	NSC-103370	-8.6	NSC-5908	-8.4
NSC-143618	-10.9	NSC-147909	-8.9	NSC-99602	-8.6	NSC-52520	-8.4
NSC-1758	-10.8	NSC-26645	-8.9	NSC-132230	-8.6	NSC-64673	-8.4
NSC-23285	-10.7	NSC-128592	-8.9	NSC-112261	-8.6	NSC-213852	-8.4

NSC-15245	-10.6	NSC-213867	-8.9	NSC-209925	-8.6	NSC-92130	-8.4
NSC-164157	-10.6	NSC-5267	-8.9	NSC-124225	-8.6	NSC-238194	-8.4
NSC-75563	-10.5	NSC-82800	-8.9	NSC-262635	-8.6	NSC-209919	-8.4
NSC-150357	-10.5	NSC-84122	-8.9	NSC-96569	-8.6	NSC-56709	-8.4
NSC-86170	-10.5	NSC-90483	-8.9	NSC-204712	-8.6	NSC-210306	-8.4
NSC-98429	-10.5	NSC-119893	-8.9	NSC-43999	-8.6	NSC-150252	-8.4
NSC-128591	-10.5	NSC-132233	-8.9	NSC-103369	-8.6	NSC-116488	-8.4
NSC-81460	-10.5	NSC-260568	-8.9	NSC-141573	-8.6	NSC-77507	-8.4
NSC-103851	-10.4	NSC-230369	-8.9	NSC-21572	-8.6	NSC-99397	-8.4
NSC-202441	-10.4	NSC-175648	-8.9	NSC-72252	-8.6	NSC-245005	-8.4
NSC-51535	-10.4	NSC-202101	-8.9	NSC-112262	-8.6	NSC-57651	-8.4
NSC-84171	-10.4	NSC-44674	-8.9	NSC-210313	-8.6	NSC-115763	-8.4
NSC-47739	-10.3	NSC-140364	-8.9	NSC-110182	-8.6	NSC-216780	-8.4
NSC-5268	-10.3	NSC-210319	-8.9	NSC-129494	-8.6	NSC-213868	-8.4
NSC-17267	-10.3	NSC-210353	-8.9	NSC-144695	-8.6	NSC-211017	-8.4
NSC-15908	-10.3	NSC-102622	-8.9	NSC-96021	-8.6	NSC-142427	-8.4
NSC-118679	-10.3	NSC-179203	-8.9	NSC-103673	-8.6	NSC-210305	-8.4
NSC-115921	-10.2	NSC-201984	-8.9	NSC-740	-8.6	NSC-35730	-8.4
NSC-13989	-10.2	NSC-128604	-8.9	NSC-117356	-8.6	NSC-135066	-8.4
NSC-153190	-10.2	NSC-186212	-8.9	NSC-210311	-8.6	NSC-5493	-8.4
NSC-153191	-10.2	NSC-247449	-8.9	NSC-165676	-8.6	NSC-99583	-8.4
NSC-128609	-10.1	NSC-110183	-8.9	NSC-197176	-8.6	NSC-20640	-8.4
NSC-13987	-10.1	NSC-196383	-8.9	NSC-54700	-8.6	NSC-217376	-8.4
NSC-82340	-10.1	NSC-225288	-8.9	NSC-193447	-8.6	NSC-109441	-8.4
NSC-125304	-10.1	NSC-179840	-8.9	NSC-226900	-8.6	NSC-144205	-8.4
NSC-244976	-10	NSC-67786	-8.9	NSC-251695	-8.6	NSC-180950	-8.4
NSC-163425	-10	NSC-127132	-8.9	NSC-134033	-8.6	NSC-76477	-8.4
NSC-126238	-10	NSC-194827	-8.9	NSC-180962	-8.6	NSC-115764	-8.4
NSC-39918	-10	NSC-226095	-8.9	NSC-211243	-8.6	NSC-164480	-8.4
NSC-90484	-9.9	NSC-12181	-8.9	NSC-13726	-8.6	NSC-150296	-8.4
NSC-7230	-9.9	NSC-174122	-8.9	NSC-35949	-8.6	NSC-153575	-8.4
NSC-212038	-9.9	NSC-87042	-8.9	NSC-36508	-8.6	NSC-11250	-8.4
NSC-212035	-9.9	NSC-32197	-8.9	NSC-9751	-8.6	NSC-250416	-8.4
NSC-74700	-9.9	NSC-177953	-8.9	NSC-117271	-8.6	NSC-21030	-8.4
NSC-12344	-9.9	NSC-95611	-8.9	NSC-120207	-8.6	NSC-84096	-8.4
NSC-114453	-9.9	NSC-210374	-8.9	NSC-117079	-8.6	NSC-179406	-8.4
NSC-116291	-9.9	NSC-204230	-8.9	NSC-211246	-8.6	NSC-9608	-8.4
NSC-70911	-9.9	NSC-167695	-8.9	NSC-51192	-8.6	NSC-135842	-8.4
NSC-123475	-9.9	NSC-156528	-8.9	NSC-98512	-8.6	NSC-248861	-8.4
NSC-120944	-9.9	NSC-262627	-8.9	NSC-92203	-8.6	NSC-215728	-8.4
NSC-123033	-9.9	NSC-64973	-8.9	NSC-163848	-8.6	NSC-251993	-8.4
NSC-163300	-9.9	NSC-37641	-8.9	NSC-130831	-8.6	NSC-231264	-8.4
NSC-97920	-9.8	NSC-231315	-8.9	NSC-112534	-8.6	NSC-79604	-8.4
NSC-87529	-9.8	NSC-126728	-8.9	NSC-148976	-8.6	NSC-30846	-8.4
NSC-120695	-9.8	NSC-156819	-8.9	NSC-90588	-8.6	NSC-211741	-8.4
NSC-25554	-9.8	NSC-84256	-8.9	NSC-97038	-8.6	NSC-8805	-8.4
NSC-60676	-9.8	NSC-187737	-8.9	NSC-17532	-8.6	NSC-158116	-8.4
NSC-14757	-9.8	NSC-226899	-8.9	NSC-125563	-8.6	NSC-231798	-8.4
NSC-2212	-9.8	NSC-117666	-8.9	NSC-156821	-8.6	NSC-179367	-8.4
NSC-39909	-9.8	NSC-159697	-8.9	NSC-173765	-8.6	NSC-144436	-8.4
NSC-179884	-9.8	NSC-115927	-8.9	NSC-101236	-8.6	NSC-37627	-8.4
NSC-23126	-9.8	NSC-246134	-8.9	NSC-101237	-8.6	NSC-235175	-8.4
NSC-164522	-9.8	NSC-132252	-8.9	NSC-111102	-8.6	NSC-12872	-8.4
NSC-210399	-9.8	NSC-128185	-8.9	NSC-111337	-8.6	NSC-257446	-8.4
NSC-30502	-9.8	NSC-210321	-8.9	NSC-117276	-8.6	NSC-238972	-8.4
NSC-53298	-9.8	NSC-72569	-8.9	NSC-157499	-8.6	NSC-85021	-8.4
NSC-37225	-9.8	NSC-159933	-8.9	NSC-235813	-8.6	NSC-167715	-8.4
NSC-123473	-9.8	NSC-125292	-8.9	NSC-106427	-8.6	NSC-211642	-8.4

NSC-121528	-9.8	NSC-72571	-8.9	NSC-86747	-8.6	NSC-90847	-8.4
NSC-121533	-9.8	NSC-135135	-8.9	NSC-97866	-8.6	NSC-14967	-8.4
NSC-122293	-9.8	NSC-251249	-8.9	NSC-93330	-8.6	NSC-227281	-8.4
NSC-163299	-9.8	NSC-244432	-8.9	NSC-262670	-8.6	NSC-103653	-8.4
NSC-87527	-9.7	NSC-38290	-8.9	NSC-154659	-8.6	NSC-216761	-8.4
NSC-179417	-9.7	NSC-249991	-8.9	NSC-23881	-8.6	NSC-113292	-8.4
NSC-79895	-9.7	NSC-90574	-8.9	NSC-251764	-8.6	NSC-76213	-8.4
NSC-122913	-9.7	NSC-64452	-8.9	NSC-237032	-8.6	NSC-12930	-8.4
NSC-12912	-9.7	NSC-121529	-8.9	NSC-205589	-8.6	NSC-105364	-8.4
NSC-59148	-9.7	NSC-123990	-8.9	NSC-118652	-8.6	NSC-58446	-8.4
NSC-39962	-9.7	NSC-87848	-8.9	NSC-152186	-8.6	NSC-99794	-8.4
NSC-97868	-9.7	NSC-211221	-8.9	NSC-140917	-8.6	NSC-125359	-8.4
NSC-152696	-9.7	NSC-235759	-8.9	NSC-201419	-8.6	NSC-201493	-8.4
NSC-13683	-9.7	NSC-263465	-8.9	NSC-86657	-8.6	NSC-45233	-8.4
NSC-90959	-9.7	NSC-57726	-8.9	NSC-164156	-8.6	NSC-122922	-8.4
NSC-91579	-9.7	NSC-75911	-8.9	NSC-176983	-8.6	NSC-264255	-8.4
NSC-212458	-9.7	NSC-212082	-8.9	NSC-78878	-8.6	NSC-68153	-8.4
NSC-117667	-9.7	NSC-143673	-8.9	NSC-145883	-8.6	NSC-164523	-8.4
NSC-219973	-9.7	NSC-81459	-8.9	NSC-51924	-8.6	NSC-1615	-8.4
NSC-134425	-9.7	NSC-168764	-8.9	NSC-125176	-8.6	NSC-93747	-8.4
NSC-123984	-9.7	NSC-261053	-8.9	NSC-82151	-8.6	NSC-114358	-8.4
NSC-126234	-9.7	NSC-261054	-8.9	NSC-83142	-8.6	NSC-160043	-8.4
NSC-126241	-9.7	NSC-135684	-8.9	NSC-53997	-8.6	NSC-234439	-8.4
NSC-126242	-9.7	NSC-72123	-8.9	NSC-152150	-8.6	NSC-67692	-8.4
NSC-121532	-9.7	NSC-221265	-8.9	NSC-260614	-8.6	NSC-211373	-8.4
NSC-125353	-9.7	NSC-12334	-8.8	NSC-53396	-8.6	NSC-255118	-8.4
NSC-67313	-9.7	NSC-30839	-8.8	NSC-231769	-8.6	NSC-143575	-8.4
NSC-107183	-9.7	NSC-230388	-8.8	NSC-203772	-8.6	NSC-82801	-8.4
NSC-23127	-9.7	NSC-90480	-8.8	NSC-118286	-8.6	NSC-128598	-8.4
NSC-128593	-9.6	NSC-128895	-8.8	NSC-40856	-8.6	NSC-10371	-8.4
NSC-171610	-9.6	NSC-30877	-8.8	NSC-76918	-8.6	NSC-118236	-8.4
NSC-247464	-9.6	NSC-35607	-8.8	NSC-70909	-8.6	NSC-148171	-8.4
NSC-247465	-9.6	NSC-59270	-8.8	NSC-258308	-8.6	NSC-155489	-8.4
NSC-98129	-9.6	NSC-30880	-8.8	NSC-99268	-8.6	NSC-147745	-8.4
NSC-250352	-9.6	NSC-76316	-8.8	NSC-35392	-8.6	NSC-37359	-8.4
NSC-39964	-9.6	NSC-37221	-8.8	NSC-152148	-8.6	NSC-159716	-8.4
NSC-23158	-9.6	NSC-210294	-8.8	NSC-66753	-8.6	NSC-47720	-8.4
NSC-91879	-9.6	NSC-121480	-8.8	NSC-12337	-8.6	NSC-108076	-8.4
NSC-254661	-9.6	NSC-88517	-8.8	NSC-256448	-8.6	NSC-103868	-8.4
NSC-116580	-9.6	NSC-84031	-8.8	NSC-116236	-8.6	NSC-204009	-8.4
NSC-159692	-9.6	NSC-87049	-8.8	NSC-159695	-8.6	NSC-32462	-8.4
NSC-39917	-9.6	NSC-133359	-8.8	NSC-121930	-8.6	NSC-113453	-8.4
NSC-122294	-9.6	NSC-164155	-8.8	NSC-38276	-8.6	NSC-80913	-8.4
NSC-121974	-9.6	NSC-164478	-8.8	NSC-114114	-8.6	NSC-260642	-8.4
NSC-120940	-9.6	NSC-201969	-8.8	NSC-168757	-8.6	NSC-117598	-8.4
NSC-211053	-9.6	NSC-135176	-8.8	NSC-19685	-8.6	NSC-170089	-8.4
NSC-120937	-9.6	NSC-211653	-8.8	NSC-94672	-8.6	NSC-157841	-8.4
NSC-116699	-9.6	NSC-255967	-8.8	NSC-34695	-8.6	NSC-63989	-8.4
NSC-251213	-9.6	NSC-135841	-8.8	NSC-254998	-8.6	NSC-109586	-8.4
NSC-126231	-9.6	NSC-115986	-8.8	NSC-120942	-8.6	NSC-49896	-8.4
NSC-126235	-9.6	NSC-193360	-8.8	NSC-129193	-8.6	NSC-45382	-8.4
NSC-7233	-9.6	NSC-260397	-8.8	NSC-127224	-8.6	NSC-173522	-8.4
NSC-121522	-9.6	NSC-39907	-8.8	NSC-5548	-8.6	NSC-121367	-8.4
NSC-149582	-9.6	NSC-102836	-8.8	NSC-95090	-8.6	NSC-233923	-8.4
NSC-28087	-9.5	NSC-120698	-8.8	NSC-177740	-8.6	NSC-210397	-8.4
NSC-156188	-9.5	NSC-120699	-8.8	NSC-127487	-8.6	NSC-210339	-8.4
NSC-27941	-9.5	NSC-180951	-8.8	NSC-13165	-8.6	NSC-50902	-8.4
NSC-90327	-9.5	NSC-72917	-8.8	NSC-22535	-8.6	NSC-146498	-8.4

NSC-152100	-9.5	NSC-145960	-8.8	NSC-123353	-8.6	NSC-245428	-8.4
NSC-128597	-9.5	NSC-16091	-8.8	NSC-165977	-8.6	NSC-121518	-8.4
NSC-174778	-9.5	NSC-39910	-8.8	NSC-91692	-8.6	NSC-210344	-8.4
NSC-134398	-9.5	NSC-125361	-8.8	NSC-239074	-8.6	NSC-239227	-8.4
NSC-106409	-9.5	NSC-227196	-8.8	NSC-157716	-8.6	NSC-134618	-8.4
NSC-196320	-9.5	NSC-250620	-8.8	NSC-123994	-8.6	NSC-39963	-8.4
NSC-122915	-9.5	NSC-88839	-8.8	NSC-202516	-8.6	NSC-162501	-8.4
NSC-37245	-9.5	NSC-50882	-8.8	NSC-144692	-8.6	NSC-122920	-8.4
NSC-132251	-9.5	NSC-229617	-8.8	NSC-74671	-8.6	NSC-167827	-8.4
NSC-174119	-9.5	NSC-142515	-8.8	NSC-129891	-8.6	NSC-21568	-8.4
NSC-211584	-9.5	NSC-211229	-8.8	NSC-103720	-8.6	NSC-164152	-8.4
NSC-156817	-9.5	NSC-57699	-8.8	NSC-239400	-8.6	NSC-117273	-8.4
NSC-81429	-9.5	NSC-112130	-8.8	NSC-80918	-8.6	NSC-184691	-8.4
NSC-43755	-9.5	NSC-75044	-8.8	NSC-231310	-8.6	NSC-139484	-8.4
NSC-45879	-9.5	NSC-81122	-8.8	NSC-173749	-8.6	NSC-244964	-8.4
NSC-35932	-9.5	NSC-106331	-8.8	NSC-90590	-8.6	NSC-245009	-8.4
NSC-115911	-9.5	NSC-1753	-8.8	NSC-129920	-8.6	NSC-156793	-8.4
NSC-90386	-9.5	NSC-196484	-8.8	NSC-123034	-8.6	NSC-231797	-8.4
NSC-118146	-9.5	NSC-167691	-8.8	NSC-211222	-8.6	NSC-180847	-8.4
NSC-251696	-9.5	NSC-164153	-8.8	NSC-244434	-8.6	NSC-224258	-8.4
NSC-122295	-9.5	NSC-31229	-8.8	NSC-51921	-8.6	NSC-256405	-8.4
NSC-120941	-9.5	NSC-15381	-8.8	NSC-244433	-8.6	NSC-107135	-8.4
NSC-123035	-9.5	NSC-132250	-8.8	NSC-247462	-8.6	NSC-156530	-8.4
NSC-71750	-9.5	NSC-211227	-8.8	NSC-75963	-8.6	NSC-15490	-8.4
NSC-212281	-9.5	NSC-70643	-8.8	NSC-126706	-8.6	NSC-154318	-8.4
NSC-12340	-9.5	NSC-230291	-8.8	NSC-95000	-8.6	NSC-4299	-8.4
NSC-35855	-9.5	NSC-125296	-8.8	NSC-222838	-8.6	NSC-45583	-8.4
NSC-123038	-9.5	NSC-114792	-8.8	NSC-152428	-8.6	NSC-40898	-8.4
NSC-123472	-9.5	NSC-112530	-8.8	NSC-221264	-8.6	NSC-57582	-8.4
NSC-87849	-9.5	NSC-167729	-8.8	NSC-247037	-8.6	NSC-134389	-8.4
NSC-125351	-9.5	NSC-86875	-8.8	NSC-131645	-8.6	NSC-162504	-8.4
NSC-5159	-9.5	NSC-39913	-8.8	NSC-42076	-8.6	NSC-1698	-8.4
NSC-123476	-9.5	NSC-211207	-8.8	NSC-45619	-8.6	NSC-44673	-8.4
NSC-149585	-9.5	NSC-75148	-8.8	NSC-230287	-8.6	NSC-170984	-8.4
NSC-149583	-9.5	NSC-167717	-8.8	NSC-166642	-8.6	NSC-62352	-8.4
NSC-39919	-9.5	NSC-134846	-8.8	NSC-245000	-8.6	NSC-114417	-8.4
NSC-257909	-9.4	NSC-2053	-8.8	NSC-129520	-8.6	NSC-92192	-8.4
NSC-99545	-9.4	NSC-177718	-8.8	NSC-66764	-8.6	NSC-115731	-8.4
NSC-208736	-9.4	NSC-97318	-8.8	NSC-119446	-8.5	NSC-204666	-8.4
NSC-90722	-9.4	NSC-168040	-8.8	NSC-144274	-8.5	NSC-35609	-8.4
NSC-128595	-9.4	NSC-144472	-8.8	NSC-144275	-8.5	NSC-144469	-8.4
NSC-97763	-9.4	NSC-162512	-8.8	NSC-138675	-8.5	NSC-23919	-8.4
NSC-244997	-9.4	NSC-124752	-8.8	NSC-70820	-8.5	NSC-68979	-8.4
NSC-125850	-9.4	NSC-154825	-8.8	NSC-97461	-8.5	NSC-210372	-8.4
NSC-128603	-9.4	NSC-117272	-8.8	NSC-70227	-8.5	NSC-156792	-8.4
NSC-125286	-9.4	NSC-134624	-8.8	NSC-172534	-8.5	NSC-230353	-8.4
NSC-125876	-9.4	NSC-162505	-8.8	NSC-229805	-8.5	NSC-192961	-8.4
NSC-241656	-9.4	NSC-2962	-8.8	NSC-230381	-8.5	NSC-122264	-8.4
NSC-141658	-9.4	NSC-230324	-8.8	NSC-68081	-8.5	NSC-208760	-8.4
NSC-173206	-9.4	NSC-219974	-8.8	NSC-143762	-8.5	NSC-47647	-8.4
NSC-203952	-9.4	NSC-103838	-8.8	NSC-125277	-8.5	NSC-135500	-8.4
NSC-80623	-9.4	NSC-87053	-8.8	NSC-30836	-8.5	NSC-138459	-8.4
NSC-179836	-9.4	NSC-212210	-8.8	NSC-179886	-8.5	NSC-131547	-8.4
NSC-31082	-9.4	NSC-146211	-8.8	NSC-186014	-8.5	NSC-218442	-8.4
NSC-101316	-9.4	NSC-76353	-8.8	NSC-68272	-8.5	NSC-238188	-8.4
NSC-156796	-9.4	NSC-148359	-8.8	NSC-80313	-8.5	NSC-56108	-8.4
NSC-111637	-9.4	NSC-97321	-8.8	NSC-87524	-8.5	NSC-134397	-8.4
NSC-170567	-9.4	NSC-231272	-8.8	NSC-205655	-8.5	NSC-61638	-8.4

NSC-168458	-9.4	NSC-201872	-8.8	NSC-136290	-8.5	NSC-120613	-8.4
NSC-159532	-9.4	NSC-101765	-8.8	NSC-30876	-8.5	NSC-168740	-8.4
NSC-125350	-9.4	NSC-75988	-8.8	NSC-19148	-8.5	NSC-90596	-8.4
NSC-74702	-9.4	NSC-235176	-8.8	NSC-99594	-8.5	NSC-162502	-8.4
NSC-252124	-9.4	NSC-22096	-8.8	NSC-259662	-8.5	NSC-50467	-8.4
NSC-43936	-9.4	NSC-125851	-8.8	NSC-230389	-8.5	NSC-82034	-8.4
NSC-137769	-9.4	NSC-142552	-8.8	NSC-120683	-8.5	NSC-63875	-8.4
NSC-170051	-9.4	NSC-247050	-8.8	NSC-179938	-8.5	NSC-49085	-8.4
NSC-120935	-9.4	NSC-5014	-8.8	NSC-123416	-8.5	NSC-163459	-8.4
NSC-120938	-9.4	NSC-255002	-8.8	NSC-216258	-8.5	NSC-234697	-8.4
NSC-45739	-9.4	NSC-262646	-8.8	NSC-136326	-8.5	NSC-211230	-8.4
NSC-120939	-9.4	NSC-181486	-8.8	NSC-155422	-8.5	NSC-51922	-8.4
NSC-120947	-9.4	NSC-135832	-8.8	NSC-171634	-8.5	NSC-131548	-8.4
NSC-126236	-9.4	NSC-251250	-8.8	NSC-230390	-8.5	NSC-129195	-8.4
NSC-36413	-9.4	NSC-125614	-8.8	NSC-125265	-8.5	NSC-52128	-8.4
NSC-123474	-9.4	NSC-179183	-8.8	NSC-36844	-8.5	NSC-76755	-8.4
NSC-123036	-9.4	NSC-38289	-8.8	NSC-126444	-8.5	NSC-67000	-8.4
NSC-121523	-9.4	NSC-99017	-8.8	NSC-264253	-8.5	NSC-106446	-8.4
NSC-192987	-9.4	NSC-99018	-8.8	NSC-238193	-8.5	NSC-162503	-8.4
NSC-121524	-9.4	NSC-69409	-8.8	NSC-109591	-8.5	NSC-58483	-8.4
NSC-211656	-9.4	NSC-120946	-8.8	NSC-129766	-8.5	NSC-151981	-8.4
NSC-127225	-9.4	NSC-212021	-8.8	NSC-196533	-8.5	NSC-125305	-8.4
NSC-240722	-9.3	NSC-50883	-8.8	NSC-21928	-8.5	NSC-66145	-8.4
NSC-38800	-9.3	NSC-93673	-8.8	NSC-11440	-8.5	NSC-28325	-8.4
NSC-136130	-9.3	NSC-103860	-8.8	NSC-44670	-8.5	NSC-217920	-8.4
NSC-113056	-9.3	NSC-131651	-8.8	NSC-184732	-8.5	NSC-206168	-8.4
NSC-62431	-9.3	NSC-91872	-8.8	NSC-68425	-8.5	NSC-35953	-8.4
NSC-84100	-9.3	NSC-93767	-8.8	NSC-55971	-8.5	NSC-230306	-8.4
NSC-55149	-9.3	NSC-229513	-8.8	NSC-216954	-8.5	NSC-91051	-8.4
NSC-231408	-9.3	NSC-76769	-8.8	NSC-240985	-8.5	NSC-205811	-8.4
NSC-79427	-9.3	NSC-227221	-8.8	NSC-64837	-8.5	NSC-226976	-8.4
NSC-211491	-9.3	NSC-11421	-8.8	NSC-157853	-8.5	NSC-80947	-8.4
NSC-210368	-9.3	NSC-121526	-8.8	NSC-43099	-8.5	NSC-117279	-8.4
NSC-77038	-9.3	NSC-123987	-8.8	NSC-216267	-8.5	NSC-129932	-8.4
NSC-13648	-9.3	NSC-93145	-8.8	NSC-16478	-8.5	NSC-101240	-8.4
NSC-179835	-9.3	NSC-169529	-8.8	NSC-160529	-8.5	NSC-168761	-8.4
NSC-262626	-9.3	NSC-211319	-8.8	NSC-3473	-8.5	NSC-70908	-8.4
NSC-52370	-9.3	NSC-120943	-8.8	NSC-211546	-8.5	NSC-16014	-8.4
NSC-80997	-9.3	NSC-39355	-8.8	NSC-92840	-8.5	NSC-111135	-8.4
NSC-239220	-9.3	NSC-238941	-8.8	NSC-92440	-8.5	NSC-94525	-8.4
NSC-118065	-9.3	NSC-43890	-8.8	NSC-108018	-8.5	NSC-30883	-8.4
NSC-156519	-9.3	NSC-43891	-8.8	NSC-206177	-8.5	NSC-127036	-8.4
NSC-39965	-9.3	NSC-128590	-8.8	NSC-250421	-8.5	NSC-168745	-8.4
NSC-139680	-9.3	NSC-146736	-8.8	NSC-229616	-8.5	NSC-152164	-8.4
NSC-80562	-9.3	NSC-129904	-8.8	NSC-179418	-8.5	NSC-63684	-8.4
NSC-162893	-9.3	NSC-75964	-8.8	NSC-240898	-8.5	NSC-48994	-8.4
NSC-143623	-9.3	NSC-144457	-8.8	NSC-135817	-8.5	NSC-23972	-8.4
NSC-136310	-9.3	NSC-35933	-8.8	NSC-217362	-8.5	NSC-159964	-8.4
NSC-46787	-9.3	NSC-7524	-8.8	NSC-162239	-8.5	NSC-86637	-8.4
NSC-90387	-9.3	NSC-21032	-8.8	NSC-119910	-8.5	NSC-23448	-8.4
NSC-179186	-9.3	NSC-11984	-8.8	NSC-124454	-8.5	NSC-1151	-8.4
NSC-119011	-9.3	NSC-145113	-8.8	NSC-126397	-8.5	NSC-144496	-8.4
NSC-219956	-9.3	NSC-89401	-8.7	NSC-126399	-8.5	NSC-128438	-8.4
NSC-211652	-9.3	NSC-116529	-8.7	NSC-47469	-8.5	NSC-264066	-8.4
NSC-45108	-9.3	NSC-17467	-8.7	NSC-204229	-8.5	NSC-212044	-8.4
NSC-211321	-9.3	NSC-12425	-8.7	NSC-12362	-8.5	NSC-211148	-8.4
NSC-103858	-9.3	NSC-168901	-8.7	NSC-75425	-8.5	NSC-104978	-8.4
NSC-124227	-9.3	NSC-30987	-8.7	NSC-203922	-8.5	NSC-144489	-8.4

NSC-211658	-9.3	NSC-77867	-8.7	NSC-121944	-8.5	NSC-134623	-8.4
NSC-121531	-9.3	NSC-240923	-8.7	NSC-37340	-8.5	NSC-175399	-8.4
NSC-187740	-9.3	NSC-81533	-8.7	NSC-135843	-8.5	NSC-154380	-8.4
NSC-126618	-9.3	NSC-128747	-8.7	NSC-7224	-8.5	NSC-13979	-8.4
NSC-168723	-9.3	NSC-134467	-8.7	NSC-96554	-8.5	NSC-73854	-8.4
NSC-21393	-9.3	NSC-81528	-8.7	NSC-264256	-8.5	NSC-187734	-8.4
NSC-244993	-9.3	NSC-129760	-8.7	NSC-20637	-8.5	NSC-144208	-8.4
NSC-263464	-9.3	NSC-131354	-8.7	NSC-119175	-8.5	NSC-88915	-8.4
NSC-60678	-9.3	NSC-15906	-8.7	NSC-102943	-8.5	NSC-187742	-8.4
NSC-97115	-9.3	NSC-116702	-8.7	NSC-99549	-8.5	NSC-261545	-8.4
NSC-82515	-9.3	NSC-149073	-8.7	NSC-180948	-8.5	NSC-161369	-8.4
NSC-146787	-9.2	NSC-84030	-8.7	NSC-180949	-8.5	NSC-159164	-8.4
NSC-121915	-9.2	NSC-109451	-8.7	NSC-22469	-8.5	NSC-212066	-8.4
NSC-163042	-9.2	NSC-135385	-8.7	NSC-99517	-8.5	NSC-143671	-8.4
NSC-31181	-9.2	NSC-210853	-8.7	NSC-117931	-8.5	NSC-122210	-8.4
NSC-149062	-9.2	NSC-12322	-8.7	NSC-41626	-8.5	NSC-126240	-8.4
NSC-122921	-9.2	NSC-260822	-8.7	NSC-30879	-8.5	NSC-220059	-8.4
NSC-91578	-9.2	NSC-159267	-8.7	NSC-2567	-8.5	NSC-103659	-8.4
NSC-134399	-9.2	NSC-216755	-8.7	NSC-72237	-8.5	NSC-170078	-8.4
NSC-23217	-9.2	NSC-129761	-8.7	NSC-21531	-8.5	NSC-254065	-8.4
NSC-39915	-9.2	NSC-42161	-8.7	NSC-142335	-8.5	NSC-51693	-8.4
NSC-20635	-9.2	NSC-135934	-8.7	NSC-55728	-8.5	NSC-168748	-8.4
NSC-128605	-9.2	NSC-90982	-8.7	NSC-154651	-8.5	NSC-168749	-8.4
NSC-128607	-9.2	NSC-3388	-8.7	NSC-115102	-8.5	NSC-117364	-8.4
NSC-174123	-9.2	NSC-3390	-8.7	NSC-120689	-8.5	NSC-102859	-8.4
NSC-12339	-9.2	NSC-137051	-8.7	NSC-128599	-8.5	NSC-126701	-8.4
NSC-24654	-9.2	NSC-55148	-8.7	NSC-119637	-8.5	NSC-236609	-8.4
NSC-12412	-9.2	NSC-174770	-8.7	NSC-135847	-8.5	NSC-255114	-8.4
NSC-99548	-9.2	NSC-204331	-8.7	NSC-118105	-8.5	NSC-249960	-8.4
NSC-213870	-9.2	NSC-217023	-8.7	NSC-10716	-8.5	NSC-254670	-8.4
NSC-179885	-9.2	NSC-125362	-8.7	NSC-106562	-8.5	NSC-247048	-8.4
NSC-103665	-9.2	NSC-158589	-8.7	NSC-144247	-8.5	NSC-149568	-8.4
NSC-49891	-9.2	NSC-125848	-8.7	NSC-211234	-8.5	NSC-75909	-8.4
NSC-210422	-9.2	NSC-125852	-8.7	NSC-159560	-8.5	NSC-212071	-8.4
NSC-128608	-9.2	NSC-109840	-8.7	NSC-174647	-8.5	NSC-52123	-8.4
NSC-179841	-9.2	NSC-111325	-8.7	NSC-39863	-8.5	NSC-146435	-8.4
NSC-179882	-9.2	NSC-136307	-8.7	NSC-90885	-8.5	NSC-104944	-8.4
NSC-37223	-9.2	NSC-185031	-8.7	NSC-49086	-8.5	NSC-51926	-8.4
NSC-231763	-9.2	NSC-71669	-8.7	NSC-175912	-8.5	NSC-92423	-8.4
NSC-42484	-9.2	NSC-45595	-8.7	NSC-143104	-8.5	NSC-138458	-8.4
NSC-156795	-9.2	NSC-164474	-8.7	NSC-127134	-8.5	NSC-73446	-8.4
NSC-135833	-9.2	NSC-35543	-8.7	NSC-55162	-8.5	NSC-114990	-8.4
NSC-69915	-9.2	NSC-75278	-8.7	NSC-201698	-8.5	NSC-151083	-8.4
NSC-132241	-9.2	NSC-60790	-8.7	NSC-125879	-8.5	NSC-38288	-8.4
NSC-72236	-9.2	NSC-96020	-8.7	NSC-156516	-8.5	NSC-235177	-8.4
NSC-254165	-9.2	NSC-68245	-8.7	NSC-136026	-8.5	NSC-34242	-8.4
NSC-200736	-9.2	NSC-116642	-8.7	NSC-135688	-8.5	NSC-210884	-8.4
NSC-247047	-9.2	NSC-164949	-8.7	NSC-146440	-8.5	NSC-123989	-8.4
NSC-135926	-9.2	NSC-131504	-8.7	NSC-90485	-8.5	NSC-91554	-8.4
NSC-12338	-9.2	NSC-156191	-8.7	NSC-216795	-8.5	NSC-170988	-8.4
NSC-260569	-9.2	NSC-7520	-8.7	NSC-147752	-8.5	NSC-23432	-8.4
NSC-85195	-9.2	NSC-23898	-8.7	NSC-143128	-8.5	NSC-69861	-8.4
NSC-160843	-9.2	NSC-36571	-8.7	NSC-118675	-8.5	NSC-128410	-8.4
NSC-119142	-9.2	NSC-118401	-8.7	NSC-82776	-8.5	NSC-164011	-8.4
NSC-249990	-9.2	NSC-216772	-8.7	NSC-106164	-8.5	NSC-160053	-8.4
NSC-75537	-9.2	NSC-127133	-8.7	NSC-200681	-8.5	NSC-94507	-8.4
NSC-180974	-9.2	NSC-72254	-8.7	NSC-81124	-8.5	NSC-170055	-8.4
NSC-152211	-9.2	NSC-180840	-8.7	NSC-174546	-8.5	NSC-239072	-8.4

NSC-152169	-9.2	NSC-128606	-8.7	NSC-67719	-8.5	NSC-106757	-8.4
NSC-47735	-9.2	NSC-75045	-8.7	NSC-96555	-8.5	NSC-216071	-8.4
NSC-69550	-9.2	NSC-37358	-8.7	NSC-210378	-8.5	NSC-81745	-8.4
NSC-38282	-9.2	NSC-119118	-8.7	NSC-59407	-8.5	NSC-72581	-8.4
NSC-120621	-9.2	NSC-118235	-8.7	NSC-139102	-8.5	NSC-254240	-8.4
NSC-106445	-9.2	NSC-107328	-8.7	NSC-129514	-8.5	NSC-7830	-8.4
NSC-111326	-9.2	NSC-213876	-8.7	NSC-132859	-8.5	NSC-65982	-8.4
NSC-142449	-9.2	NSC-230360	-8.7	NSC-7228	-8.5	NSC-92316	-8.4
NSC-115768	-9.2	NSC-122212	-8.7	NSC-156525	-8.5	NSC-132579	-8.3
NSC-123037	-9.2	NSC-105360	-8.7	NSC-162511	-8.5	NSC-105464	-8.3
NSC-30688	-9.2	NSC-211226	-8.7	NSC-149802	-8.5	NSC-105552	-8.3
NSC-168756	-9.2	NSC-211519	-8.7	NSC-125908	-8.5	NSC-113312	-8.3
NSC-107088	-9.2	NSC-146439	-8.7	NSC-158591	-8.5	NSC-113309	-8.3
NSC-125349	-9.2	NSC-243842	-8.7	NSC-115729	-8.5	NSC-186015	-8.3
NSC-115000	-9.2	NSC-17360	-8.7	NSC-3348	-8.5	NSC-79586	-8.3
NSC-32997	-9.2	NSC-150421	-8.7	NSC-156794	-8.5	NSC-12315	-8.3
NSC-51543	-9.2	NSC-227205	-8.7	NSC-109437	-8.5	NSC-156247	-8.3
NSC-261415	-9.2	NSC-230292	-8.7	NSC-211488	-8.5	NSC-90378	-8.3
NSC-12342	-9.2	NSC-80915	-8.7	NSC-29079	-8.5	NSC-82158	-8.3
NSC-114999	-9.2	NSC-70931	-8.7	NSC-11239	-8.5	NSC-31297	-8.3
NSC-105466	-9.2	NSC-111128	-8.7	NSC-195204	-8.5	NSC-116530	-8.3
NSC-144425	-9.1	NSC-225290	-8.7	NSC-107144	-8.5	NSC-219498	-8.3
NSC-104545	-9.1	NSC-129991	-8.7	NSC-130108	-8.5	NSC-67716	-8.3
NSC-196534	-9.1	NSC-129993	-8.7	NSC-260605	-8.5	NSC-87522	-8.3
NSC-72667	-9.1	NSC-263162	-8.7	NSC-7534	-8.5	NSC-205727	-8.3
NSC-152542	-9.1	NSC-135136	-8.7	NSC-117269	-8.5	NSC-47919	-8.3
NSC-12363	-9.1	NSC-211489	-8.7	NSC-134620	-8.5	NSC-31000	-8.3
NSC-230387	-9.1	NSC-174552	-8.7	NSC-249234	-8.5	NSC-97911	-8.3
NSC-28088	-9.1	NSC-132040	-8.7	NSC-76216	-8.5	NSC-112497	-8.3
NSC-122914	-9.1	NSC-57735	-8.7	NSC-190526	-8.5	NSC-120667	-8.3
NSC-36599	-9.1	NSC-224428	-8.7	NSC-148154	-8.5	NSC-135168	-8.3
NSC-7226	-9.1	NSC-7227	-8.7	NSC-75910	-8.5	NSC-106683	-8.3
NSC-113010	-9.1	NSC-141680	-8.7	NSC-117274	-8.5	NSC-75006	-8.3
NSC-241844	-9.1	NSC-129962	-8.7	NSC-125298	-8.5	NSC-235777	-8.3
NSC-11241	-9.1	NSC-127044	-8.7	NSC-121342	-8.5	NSC-14081	-8.3
NSC-201976	-9.1	NSC-211836	-8.7	NSC-142265	-8.5	NSC-99519	-8.3
NSC-156816	-9.1	NSC-99281	-8.7	NSC-209840	-8.5	NSC-156322	-8.3
NSC-135917	-9.1	NSC-39914	-8.7	NSC-107133	-8.5	NSC-174924	-8.3
NSC-260829	-9.1	NSC-74588	-8.7	NSC-107134	-8.5	NSC-76320	-8.3
NSC-143615	-9.1	NSC-43996	-8.7	NSC-69293	-8.5	NSC-12410	-8.3
NSC-164525	-9.1	NSC-75113	-8.7	NSC-97867	-8.5	NSC-120668	-8.3
NSC-210367	-9.1	NSC-243779	-8.7	NSC-109439	-8.5	NSC-171474	-8.3
NSC-117811	-9.1	NSC-66999	-8.7	NSC-132483	-8.5	NSC-78793	-8.3
NSC-212061	-9.1	NSC-115018	-8.7	NSC-132249	-8.5	NSC-39953	-8.3
NSC-210373	-9.1	NSC-106425	-8.7	NSC-101235	-8.5	NSC-150103	-8.3
NSC-101233	-9.1	NSC-245023	-8.7	NSC-6448	-8.5	NSC-109482	-8.3
NSC-98513	-9.1	NSC-91881	-8.7	NSC-105780	-8.5	NSC-46539	-8.3
NSC-119285	-9.1	NSC-135659	-8.7	NSC-134621	-8.5	NSC-186884	-8.3
NSC-247468	-9.1	NSC-211807	-8.7	NSC-143921	-8.5	NSC-167427	-8.3
NSC-91772	-9.1	NSC-127622	-8.7	NSC-160129	-8.5	NSC-132507	-8.3
NSC-119143	-9.1	NSC-44756	-8.7	NSC-129881	-8.5	NSC-138664	-8.3
NSC-91880	-9.1	NSC-125297	-8.7	NSC-34243	-8.5	NSC-115122	-8.3
NSC-174610	-9.1	NSC-134625	-8.7	NSC-142843	-8.5	NSC-163041	-8.3
NSC-115534	-9.1	NSC-78868	-8.7	NSC-162510	-8.5	NSC-146519	-8.3
NSC-260611	-9.1	NSC-164521	-8.7	NSC-170090	-8.5	NSC-76321	-8.3
NSC-156235	-9.1	NSC-117277	-8.7	NSC-169531	-8.5	NSC-213914	-8.3
NSC-27959	-9.1	NSC-108391	-8.7	NSC-152167	-8.5	NSC-140063	-8.3
NSC-99199	-9.1	NSC-173753	-8.7	NSC-239511	-8.5	NSC-219247	-8.3

NSC-117665	-9.1	NSC-107454	-8.7	NSC-97758	-8.5	NSC-70602	-8.3
NSC-140918	-9.1	NSC-117275	-8.7	NSC-38954	-8.5	NSC-32461	-8.3
NSC-144039	-9.1	NSC-187738	-8.7	NSC-230290	-8.5	NSC-120673	-8.3
NSC-81403	-9.1	NSC-141679	-8.7	NSC-25937	-8.5	NSC-165699	-8.3
NSC-85443	-9.1	NSC-170061	-8.7	NSC-90583	-8.5	NSC-149050	-8.3
NSC-117633	-9.1	NSC-212218	-8.7	NSC-6755	-8.5	NSC-42065	-8.3
NSC-96730	-9.1	NSC-105398	-8.7	NSC-134396	-8.5	NSC-155209	-8.3
NSC-123992	-9.1	NSC-80698	-8.7	NSC-134969	-8.5	NSC-87838	-8.3
NSC-236657	-9.1	NSC-159698	-8.7	NSC-134982	-8.5	NSC-89861	-8.3
NSC-159694	-9.1	NSC-80699	-8.7	NSC-115174	-8.5	NSC-123478	-8.3
NSC-43945	-9.1	NSC-66754	-8.7	NSC-156818	-8.5	NSC-250415	-8.3
NSC-210944	-9.1	NSC-66755	-8.7	NSC-159370	-8.5	NSC-127038	-8.3
NSC-123986	-9.1	NSC-143507	-8.7	NSC-146028	-8.5	NSC-18054	-8.3
NSC-211626	-9.1	NSC-8111	-8.7	NSC-105793	-8.5	NSC-18313	-8.3
NSC-12343	-9.1	NSC-124224	-8.7	NSC-187744	-8.5	NSC-167330	-8.3
NSC-211292	-9.1	NSC-172938	-8.7	NSC-134626	-8.5	NSC-177862	-8.3
NSC-67586	-9.1	NSC-202482	-8.7	NSC-72579	-8.5	NSC-164151	-8.3
NSC-63675	-9.1	NSC-17243	-8.7	NSC-15414	-8.5	NSC-180550	-8.3
NSC-52311	-9.1	NSC-137767	-8.7	NSC-9160	-8.5	NSC-12336	-8.3
NSC-96731	-9.1	NSC-259660	-8.7	NSC-237027	-8.5	NSC-125034	-8.3
NSC-151181	-9.1	NSC-93068	-8.7	NSC-99628	-8.5	NSC-13097	-8.3
NSC-21392	-9.1	NSC-1378	-8.7	NSC-139661	-8.5	NSC-174789	-8.3
NSC-166818	-9.1	NSC-96729	-8.7	NSC-56346	-8.5	NSC-112500	-8.3
NSC-140781	-9.1	NSC-204511	-8.7	NSC-251673	-8.5	NSC-215571	-8.3
NSC-252174	-9.1	NSC-103113	-8.7	NSC-122272	-8.5	NSC-21969	-8.3
NSC-38989	-9.1	NSC-129827	-8.7	NSC-171250	-8.5	NSC-216273	-8.3
NSC-129845	-9.1	NSC-254164	-8.7	NSC-230295	-8.5	NSC-174934	-8.3
NSC-136589	-9.1	NSC-64112	-8.7	NSC-114732	-8.5	NSC-18958	-8.3
NSC-22909	-9.1	NSC-143355	-8.7	NSC-132253	-8.5	NSC-34614	-8.3
NSC-56820	-9.1	NSC-7804	-8.7	NSC-117278	-8.5	NSC-73099	-8.3
NSC-66766	-9.1	NSC-83073	-8.7	NSC-180510	-8.5	NSC-218392	-8.3
NSC-206176	-9	NSC-149581	-8.7	NSC-144951	-8.5	NSC-115732	-8.3
NSC-94029	-9	NSC-123988	-8.7	NSC-7521	-8.5	NSC-18458	-8.3
NSC-104547	-9	NSC-211662	-8.7	NSC-93331	-8.5	NSC-107129	-8.3
NSC-104544	-9	NSC-123471	-8.7	NSC-43869	-8.5	NSC-169482	-8.3
NSC-157388	-9	NSC-77034	-8.7	NSC-159374	-8.5	NSC-173083	-8.3
NSC-127130	-9	NSC-140119	-8.7	NSC-30664	-8.5	NSC-109943	-8.3
NSC-167883	-9	NSC-88182	-8.7	NSC-227219	-8.5	NSC-99527	-8.3
NSC-154018	-9	NSC-226673	-8.7	NSC-212076	-8.5	NSC-211014	-8.3
NSC-3410	-9	NSC-126221	-8.7	NSC-109161	-8.5	NSC-39954	-8.3
NSC-132228	-9	NSC-79640	-8.7	NSC-109163	-8.5	NSC-117199	-8.3
NSC-99589	-9	NSC-124228	-8.7	NSC-14500	-8.5	NSC-173735	-8.3
NSC-149074	-9	NSC-31048	-8.7	NSC-176967	-8.5	NSC-250414	-8.3
NSC-179416	-9	NSC-186024	-8.7	NSC-50349	-8.5	NSC-143619	-8.3
NSC-123831	-9	NSC-176325	-8.7	NSC-170198	-8.5	NSC-77870	-8.3
NSC-106267	-9	NSC-239399	-8.7	NSC-144040	-8.5	NSC-54037	-8.3
NSC-97760	-9	NSC-170062	-8.7	NSC-5499	-8.5	NSC-31227	-8.3
NSC-122391	-9	NSC-212423	-8.7	NSC-122047	-8.5	NSC-122390	-8.3
NSC-15973	-9	NSC-47746	-8.7	NSC-92195	-8.5	NSC-48307	-8.3
NSC-89778	-9	NSC-125175	-8.7	NSC-170155	-8.5	NSC-48589	-8.3
NSC-159521	-9	NSC-12986	-8.7	NSC-136143	-8.5	NSC-63988	-8.3
NSC-97764	-9	NSC-156847	-8.7	NSC-142847	-8.5	NSC-123402	-8.3
NSC-16842	-9	NSC-187735	-8.7	NSC-159628	-8.5	NSC-118820	-8.3
NSC-120707	-9	NSC-52131	-8.7	NSC-94998	-8.5	NSC-220534	-8.3
NSC-1878	-9	NSC-12456	-8.7	NSC-93675	-8.5	NSC-129764	-8.3
NSC-150777	-9	NSC-241196	-8.7	NSC-7795	-8.5	NSC-231613	-8.3
NSC-118673	-9	NSC-210890	-8.7	NSC-41838	-8.5	NSC-120678	-8.3
NSC-8603	-9	NSC-52139	-8.7	NSC-129196	-8.5	NSC-136094	-8.3

NSC-146746	-9	NSC-87850	-8.7	NSC-76770	-8.5	NSC-55449	-8.3
NSC-134356	-9	NSC-80315	-8.7	NSC-144491	-8.5	NSC-126404	-8.3
NSC-132229	-9	NSC-170107	-8.7	NSC-45612	-8.5	NSC-164482	-8.3
NSC-164866	-9	NSC-211390	-8.7	NSC-143112	-8.5	NSC-37632	-8.3
NSC-164477	-9	NSC-66767	-8.7	NSC-143114	-8.5	NSC-120696	-8.3
NSC-150355	-9	NSC-260573	-8.6	NSC-125306	-8.5	NSC-63008	-8.3
NSC-79552	-9	NSC-164163	-8.6	NSC-62793	-8.5	NSC-120665	-8.3
NSC-210381	-9	NSC-4234	-8.6	NSC-29137	-8.5	NSC-79267	-8.3
NSC-31231	-9	NSC-106891	-8.6	NSC-120945	-8.5	NSC-103371	-8.3
NSC-145963	-9	NSC-152543	-8.6	NSC-92173	-8.5	NSC-74605	-8.3
NSC-210379	-9	NSC-99518	-8.6	NSC-212247	-8.5	NSC-205875	-8.3
NSC-122919	-9	NSC-155399	-8.6	NSC-135070	-8.5	NSC-144068	-8.3
NSC-35966	-9	NSC-205654	-8.6	NSC-51531	-8.5	NSC-81123	-8.3
NSC-115931	-9	NSC-89127	-8.6	NSC-123123	-8.5	NSC-245862	-8.3
NSC-125846	-9	NSC-16079	-8.6	NSC-251534	-8.5	NSC-159512	-8.3
NSC-211224	-9	NSC-76164	-8.6	NSC-156797	-8.5	NSC-152416	-8.3
NSC-13728	-9	NSC-150358	-8.6	NSC-27621	-8.5	NSC-211783	-8.3
NSC-159540	-9	NSC-99592	-8.6	NSC-140932	-8.5	NSC-45211	-8.3
NSC-260570	-9	NSC-127936	-8.6	NSC-119837	-8.5	NSC-165567	-8.3
NSC-179883	-9	NSC-241608	-8.6	NSC-135679	-8.5	NSC-148939	-8.3
NSC-35350	-9	NSC-147680	-8.6	NSC-104945	-8.5	NSC-153591	-8.3
NSC-166390	-9	NSC-217371	-8.6	NSC-105131	-8.5	NSC-216758	-8.3
NSC-57144	-9	NSC-81525	-8.6	NSC-163493	-8.5	NSC-100274	-8.3
NSC-15244	-9	NSC-24952	-8.6	NSC-246104	-8.5	NSC-138485	-8.3
NSC-107658	-9	NSC-81527	-8.6	NSC-18506	-8.5	NSC-214026	-8.3
NSC-39911	-9	NSC-83292	-8.6	NSC-114920	-8.5	NSC-377	-8.3
NSC-103663	-9	NSC-36705	-8.6	NSC-226905	-8.5	NSC-234669	-8.3
NSC-39912	-9	NSC-159268	-8.6	NSC-202371	-8.5	NSC-62430	-8.3
NSC-86656	-9	NSC-61900	-8.6	NSC-105130	-8.5	NSC-241470	-8.3
NSC-14167	-9	NSC-193428	-8.6	NSC-168739	-8.5	NSC-210435	-8.3
NSC-122811	-9	NSC-41100	-8.6	NSC-154382	-8.5	NSC-94688	-8.3
NSC-210354	-9	NSC-254946	-8.6	NSC-118695	-8.5	NSC-203974	-8.3
NSC-127636	-9	NSC-106262	-8.6	NSC-144087	-8.5	NSC-85314	-8.3
NSC-251698	-9	NSC-13721	-8.6	NSC-156848	-8.5	NSC-167687	-8.3
NSC-90385	-9	NSC-121481	-8.6	NSC-218486	-8.5	NSC-145967	-8.3
NSC-17249	-9	NSC-32940	-8.6	NSC-264073	-8.5	NSC-205632	-8.3
NSC-38945	-9	NSC-33358	-8.6	NSC-154387	-8.5	NSC-104147	-8.3
NSC-244996	-9	NSC-246095	-8.6	NSC-38285	-8.5	NSC-264719	-8.3
NSC-16080	-9	NSC-99547	-8.6	NSC-38284	-8.5	NSC-127935	-8.3
NSC-73102	-9	NSC-216805	-8.6	NSC-30258	-8.5	NSC-167804	-8.3
NSC-138176	-9	NSC-201873	-8.6	NSC-231916	-8.5	NSC-216759	-8.3
NSC-226897	-9	NSC-172523	-8.6	NSC-241198	-8.5	NSC-31226	-8.3
NSC-250351	-9	NSC-144308	-8.6	NSC-43983	-8.5	NSC-123394	-8.3
NSC-90318	-9	NSC-115992	-8.6	NSC-135499	-8.5	NSC-123398	-8.3
NSC-28093	-9	NSC-254927	-8.6	NSC-70417	-8.5	NSC-159446	-8.3
NSC-103661	-9	NSC-243865	-8.6	NSC-105298	-8.5	NSC-224298	-8.3
NSC-66752	-9	NSC-125354	-8.6	NSC-47747	-8.5	NSC-249271	-8.3
NSC-128172	-9	NSC-22669	-8.6	NSC-129871	-8.5	NSC-101869	-8.3
NSC-96728	-9	NSC-99864	-8.6	NSC-246132	-8.5	NSC-15909	-8.3
NSC-123977	-9	NSC-226137	-8.6	NSC-47768	-8.5	NSC-123793	-8.3
NSC-120936	-9	NSC-67691	-8.6	NSC-219851	-8.5	NSC-133489	-8.3
NSC-264720	-9	NSC-56962	-8.6	NSC-139221	-8.5	NSC-73527	-8.3
NSC-219852	-9	NSC-120296	-8.6	NSC-52135	-8.5	NSC-226884	-8.3
NSC-126239	-9	NSC-145965	-8.6	NSC-219977	-8.5	NSC-231269	-8.3
NSC-126243	-9	NSC-106429	-8.6	NSC-256926	-8.5	NSC-123311	-8.3
NSC-129907	-9	NSC-150773	-8.6	NSC-129865	-8.5	NSC-103647	-8.3
NSC-93674	-9	NSC-229620	-8.6	NSC-90594	-8.5	NSC-264870	-8.3
NSC-91555	-9	NSC-115112	-8.6	NSC-121329	-8.4	NSC-150350	-8.3

NSC-108530	-9	NSC-31131	-8.6	NSC-146592	-8.4	NSC-104119	-8.3
NSC-212277	-9	NSC-129955	-8.6	NSC-241039	-8.4	NSC-90384	-8.3
NSC-247049	-9	NSC-75521	-8.6	NSC-75003	-8.4	NSC-226159	-8.3
NSC-126233	-9	NSC-153680	-8.6	NSC-176040	-8.4	NSC-211209	-8.3
NSC-150489	-9	NSC-204232	-8.6	NSC-201562	-8.4	NSC-86710	-8.3
NSC-229643	-9	NSC-99606	-8.6	NSC-133357	-8.4	NSC-21565	-8.3
NSC-126237	-9	NSC-216763	-8.6	NSC-120666	-8.4	NSC-250621	-8.3
NSC-66765	-9	NSC-106430	-8.6	NSC-82533	-8.4	NSC-167698	-8.3
NSC-32996	-9	NSC-33588	-8.6	NSC-64921	-8.4	NSC-4302	-8.3
NSC-13991	-9	NSC-122923	-8.6	NSC-123424	-8.4	NSC-216788	-8.3
NSC-12335	-8.9	NSC-106443	-8.6	NSC-78480	-8.4	NSC-150440	-8.3
NSC-153625	-8.9	NSC-164476	-8.6	NSC-256398	-8.4	NSC-124351	-8.3
NSC-155418	-8.9	NSC-49636	-8.6	NSC-23125	-8.4	NSC-138446	-8.3
NSC-28035	-8.9	NSC-80118	-8.6	NSC-75907	-8.4	NSC-18877	-8.3
NSC-230386	-8.9	NSC-20636	-8.6	NSC-171462	-8.4	NSC-48750	-8.3
NSC-55972	-8.9	NSC-155242	-8.6	NSC-30878	-8.4	NSC-254066	-8.3
NSC-64923	-8.9	NSC-69408	-8.6	NSC-90781	-8.4	NSC-255296	-8.3
NSC-193382	-8.9	NSC-164879	-8.6	NSC-48263	-8.4	NSC-44114	-8.3
NSC-109822	-8.9	NSC-79051	-8.6	NSC-135171	-8.4	NSC-113096	-8.3
NSC-26684	-8.9	NSC-88841	-8.6	NSC-139466	-8.4	NSC-114794	-8.3
NSC-52526	-8.9	NSC-121868	-8.6	NSC-89539	-8.4	NSC-144674	-8.3
NSC-173770	-8.9	NSC-134694	-8.6	NSC-205857	-8.4	NSC-164475	-8.3
NSC-119440	-8.9	NSC-45212	-8.6	NSC-4725	-8.4	NSC-45234	-8.3
NSC-153611	-8.9	NSC-55141	-8.6	NSC-12321	-8.4	NSC-109778	-8.3
NSC-156177	-8.9	NSC-219855	-8.6	NSC-41106	-8.4	NSC-226136	-8.3
NSC-135530	-8.9	NSC-245423	-8.6	NSC-77522	-8.4	NSC-124748	-8.3
NSC-260567	-8.9	NSC-114455	-8.6	NSC-75015	-8.4	NSC-27592	-8.3
NSC-171279	-8.9	NSC-210337	-8.6	NSC-99524	-8.4	NSC-82181	-8.3
NSC-164520	-8.9	NSC-107177	-8.6	NSC-209861	-8.4	NSC-224250	-8.3
NSC-95171	-8.9	NSC-55151	-8.6	NSC-216259	-8.4	NSC-224427	-8.3
NSC-213775	-8.9	NSC-74511	-8.6	NSC-102068	-8.4	NSC-28557	-8.3

Table S2-B. Results of Tier-II docking: Top 2000 compounds against the SHP099-bound SHP2 E76K mutant MD structure. These compounds were selected based on binding affinity scores from Tier-I docking. (unit: kcal/mol)

Molecule-ID	Affinity	Molecule-ID	Affinity	Molecule-ID	Affinity	Molecule-ID	Affinity
NSC-75563	-8.8	NSC-92440	-6.7	NSC-219977	-6.4	NSC-52128	-6
NSC-117812	-8.8	NSC-95611	-6.7	NSC-220534	-6.4	NSC-56709	-6
NSC-244996	-8.7	NSC-96731	-6.7	NSC-224428	-6.4	NSC-59407	-6
NSC-84171	-8.5	NSC-97318	-6.7	NSC-226673	-6.4	NSC-62431	-6
NSC-139812	-8.5	NSC-97763	-6.7	NSC-229620	-6.4	NSC-62793	-6
NSC-60678	-8.3	NSC-97920	-6.7	NSC-230360	-6.4	NSC-64837	-6
NSC-219973	-8.3	NSC-99017	-6.7	NSC-230369	-6.4	NSC-68153	-6
NSC-14757	-8.1	NSC-99018	-6.7	NSC-235777	-6.4	NSC-68979	-6
NSC-20635	-8.1	NSC-99199	-6.7	NSC-235813	-6.4	NSC-70417	-6
NSC-51543	-8.1	NSC-101233	-6.7	NSC-240722	-6.4	NSC-70820	-6
NSC-236657	-8.1	NSC-112261	-6.7	NSC-240985	-6.4	NSC-72917	-6
NSC-35855	-8	NSC-115931	-6.7	NSC-247037	-6.4	NSC-75044	-6
NSC-39919	-7.9	NSC-116291	-6.7	NSC-254240	-6.4	NSC-75425	-6
NSC-74702	-7.9	NSC-117269	-6.7	NSC-254927	-6.4	NSC-81124	-6
NSC-91579	-7.9	NSC-120936	-6.7	NSC-260570	-6.4	NSC-83292	-6
NSC-106445	-7.9	NSC-121524	-6.7	NSC-260829	-6.4	NSC-84030	-6
NSC-125304	-7.9	NSC-122047	-6.7	NSC-264255	-6.4	NSC-86657	-6
NSC-144457	-7.9	NSC-122272	-6.7	NSC-264256	-6.4	NSC-88839	-6
NSC-163300	-7.9	NSC-122294	-6.7	NSC-264870	-6.4	NSC-90781	-6
NSC-173206	-7.9	NSC-122919	-6.7	NSC-377	-6.3	NSC-90885	-6
NSC-192987	-7.9	NSC-123471	-6.7	NSC-9160	-6.3	NSC-92195	-6
NSC-32997	-7.8	NSC-123475	-6.7	NSC-9751	-6.3	NSC-93330	-6
NSC-38989	-7.8	NSC-125349	-6.7	NSC-11239	-6.3	NSC-94688	-6

NSC-43945	-7.8	NSC-125908	-6.7	NSC-11440	-6.3	NSC-95000	-6
NSC-60676	-7.8	NSC-126233	-6.7	NSC-12872	-6.3	NSC-99281	-6
NSC-63684	-7.8	NSC-126234	-6.7	NSC-15490	-6.3	NSC-99583	-6
NSC-107328	-7.8	NSC-126235	-6.7	NSC-17360	-6.3	NSC-99594	-6
NSC-245023	-7.8	NSC-126236	-6.7	NSC-22909	-6.3	NSC-101316	-6
NSC-252174	-7.8	NSC-127224	-6.7	NSC-23448	-6.3	NSC-102622	-6
NSC-20640	-7.7	NSC-128438	-6.7	NSC-27621	-6.3	NSC-104547	-6
NSC-30502	-7.7	NSC-134397	-6.7	NSC-28088	-6.3	NSC-104978	-6
NSC-32996	-7.7	NSC-135135	-6.7	NSC-28093	-6.3	NSC-107129	-6
NSC-39917	-7.7	NSC-135688	-6.7	NSC-31000	-6.3	NSC-112497	-6
NSC-47739	-7.7	NSC-135817	-6.7	NSC-31131	-6.3	NSC-112534	-6
NSC-53298	-7.7	NSC-136026	-6.7	NSC-34243	-6.3	NSC-114114	-6
NSC-67313	-7.7	NSC-138446	-6.7	NSC-35609	-6.3	NSC-118236	-6
NSC-93674	-7.7	NSC-138458	-6.7	NSC-36599	-6.3	NSC-119440	-6
NSC-103663	-7.7	NSC-139661	-6.7	NSC-39863	-6.3	NSC-119893	-6
NSC-107088	-7.7	NSC-141680	-6.7	NSC-43891	-6.3	NSC-121481	-6
NSC-114453	-7.7	NSC-144040	-6.7	NSC-44670	-6.3	NSC-123398	-6
NSC-143618	-7.7	NSC-146746	-6.7	NSC-44673	-6.3	NSC-123478	-6
NSC-150357	-7.7	NSC-148171	-6.7	NSC-47720	-6.3	NSC-124224	-6
NSC-251250	-7.7	NSC-152169	-6.7	NSC-52370	-6.3	NSC-124225	-6
NSC-15908	-7.6	NSC-156796	-6.7	NSC-53396	-6.3	NSC-125034	-6
NSC-20636	-7.6	NSC-159267	-6.7	NSC-54037	-6.3	NSC-126239	-6
NSC-121974	-7.6	NSC-159521	-6.7	NSC-55972	-6.3	NSC-126397	-6
NSC-125176	-7.6	NSC-164152	-6.7	NSC-56346	-6.3	NSC-126444	-6
NSC-142449	-7.6	NSC-164478	-6.7	NSC-62430	-6.3	NSC-128606	-6
NSC-153190	-7.6	NSC-167695	-6.7	NSC-63008	-6.3	NSC-128895	-6
NSC-153191	-7.6	NSC-167883	-6.7	NSC-64112	-6.3	NSC-129764	-6
NSC-163425	-7.6	NSC-168756	-6.7	NSC-64452	-6.3	NSC-129904	-6
NSC-250351	-7.6	NSC-168757	-6.7	NSC-71669	-6.3	NSC-131504	-6
NSC-251249	-7.6	NSC-170055	-6.7	NSC-72571	-6.3	NSC-131548	-6
NSC-252124	-7.6	NSC-175648	-6.7	NSC-73102	-6.3	NSC-133357	-6
NSC-23127	-7.5	NSC-179203	-6.7	NSC-75278	-6.3	NSC-136143	-6
NSC-39918	-7.5	NSC-179406	-6.7	NSC-75910	-6.3	NSC-142265	-6
NSC-45108	-7.5	NSC-200681	-6.7	NSC-76216	-6.3	NSC-142847	-6
NSC-72581	-7.5	NSC-201969	-6.7	NSC-76769	-6.3	NSC-143128	-6
NSC-73854	-7.5	NSC-201984	-6.7	NSC-78868	-6.3	NSC-143619	-6
NSC-97764	-7.5	NSC-202441	-6.7	NSC-80698	-6.3	NSC-144247	-6
NSC-103661	-7.5	NSC-204666	-6.7	NSC-80947	-6.3	NSC-145960	-6
NSC-103858	-7.5	NSC-211390	-6.7	NSC-81403	-6.3	NSC-150350	-6
NSC-106331	-7.5	NSC-216258	-6.7	NSC-82181	-6.3	NSC-152144	-6
NSC-118695	-7.5	NSC-216273	-6.7	NSC-82800	-6.3	NSC-154018	-6
NSC-127036	-7.5	NSC-225288	-6.7	NSC-86747	-6.3	NSC-155209	-6
NSC-159692	-7.5	NSC-227221	-6.7	NSC-87049	-6.3	NSC-155422	-6
NSC-170051	-7.5	NSC-230387	-6.7	NSC-89539	-6.3	NSC-156528	-6
NSC-170988	-7.5	NSC-234669	-6.7	NSC-90480	-6.3	NSC-156792	-6
NSC-171610	-7.5	NSC-239511	-6.7	NSC-90583	-6.3	NSC-156797	-6
NSC-180974	-7.5	NSC-243865	-6.7	NSC-90588	-6.3	NSC-157499	-6
NSC-2212	-7.4	NSC-245428	-6.7	NSC-93675	-6.3	NSC-157841	-6
NSC-13987	-7.4	NSC-250416	-6.7	NSC-96021	-6.3	NSC-159964	-6
NSC-35932	-7.4	NSC-251696	-6.7	NSC-97321	-6.3	NSC-160053	-6
NSC-39355	-7.4	NSC-256926	-6.7	NSC-99268	-6.3	NSC-163459	-6
NSC-39909	-7.4	NSC-260567	-6.7	NSC-99589	-6.3	NSC-164482	-6
NSC-39913	-7.4	NSC-260568	-6.7	NSC-99602	-6.3	NSC-169531	-6
NSC-67586	-7.4	NSC-1878	-6.6	NSC-102943	-6.3	NSC-173735	-6
NSC-72236	-7.4	NSC-6755	-6.6	NSC-103647	-6.3	NSC-174610	-6
NSC-75911	-7.4	NSC-7233	-6.6	NSC-104544	-6.3	NSC-186024	-6
NSC-82340	-7.4	NSC-7795	-6.6	NSC-105398	-6.3	NSC-186884	-6
NSC-82515	-7.4	NSC-11241	-6.6	NSC-105793	-6.3	NSC-201698	-6

NSC-90483	-7.4	NSC-12986	-6.6	NSC-106562	-6.3	NSC-203974	-6
NSC-90959	-7.4	NSC-26645	-6.6	NSC-107133	-6.3	NSC-204009	-6
NSC-91555	-7.4	NSC-30836	-6.6	NSC-109591	-6.3	NSC-206176	-6
NSC-98129	-7.4	NSC-30883	-6.6	NSC-109840	-6.3	NSC-211783	-6
NSC-103851	-7.4	NSC-31226	-6.6	NSC-110182	-6.3	NSC-212038	-6
NSC-105130	-7.4	NSC-35543	-6.6	NSC-112130	-6.3	NSC-212281	-6
NSC-150489	-7.4	NSC-35607	-6.6	NSC-113096	-6.3	NSC-216780	-6
NSC-166818	-7.4	NSC-35953	-6.6	NSC-114792	-6.3	NSC-216805	-6
NSC-170062	-7.4	NSC-38285	-6.6	NSC-114794	-6.3	NSC-217362	-6
NSC-179183	-7.4	NSC-39914	-6.6	NSC-115992	-6.3	NSC-217371	-6
NSC-179836	-7.4	NSC-42076	-6.6	NSC-116702	-6.3	NSC-218486	-6
NSC-202516	-7.4	NSC-44674	-6.6	NSC-117199	-6.3	NSC-224298	-6
NSC-244993	-7.4	NSC-45382	-6.6	NSC-117273	-6.3	NSC-226095	-6
NSC-250352	-7.4	NSC-46787	-6.6	NSC-118401	-6.3	NSC-226137	-6
NSC-251213	-7.4	NSC-59270	-6.6	NSC-118673	-6.3	NSC-226900	-6
NSC-7804	-7.3	NSC-60790	-6.6	NSC-121518	-6.3	NSC-226976	-6
NSC-18958	-7.3	NSC-64973	-6.6	NSC-122210	-6.3	NSC-230386	-6
NSC-21032	-7.3	NSC-66764	-6.6	NSC-123989	-6.3	NSC-230389	-6
NSC-34614	-7.3	NSC-67000	-6.6	NSC-124227	-6.3	NSC-231269	-6
NSC-35933	-7.3	NSC-67719	-6.6	NSC-124748	-6.3	NSC-231613	-6
NSC-39962	-7.3	NSC-70911	-6.6	NSC-125850	-6.3	NSC-241196	-6
NSC-39965	-7.3	NSC-72254	-6.6	NSC-126404	-6.3	NSC-241608	-6
NSC-45739	-7.3	NSC-75988	-6.6	NSC-127038	-6.3	NSC-245862	-6
NSC-59148	-7.3	NSC-76316	-6.6	NSC-127044	-6.3	NSC-256405	-6
NSC-67786	-7.3	NSC-77867	-6.6	NSC-127133	-6.3	NSC-257446	-6
NSC-74671	-7.3	NSC-78480	-6.6	NSC-127134	-6.3	NSC-260573	-6
NSC-91879	-7.3	NSC-80997	-6.6	NSC-128410	-6.3	NSC-262646	-6
NSC-93673	-7.3	NSC-81123	-6.6	NSC-128590	-6.3	NSC-263465	-6
NSC-106430	-7.3	NSC-82034	-6.6	NSC-128604	-6.3	NSC-11250	-5.9
NSC-111637	-7.3	NSC-82151	-6.6	NSC-131354	-6.3	NSC-12425	-5.9
NSC-124752	-7.3	NSC-85314	-6.6	NSC-131645	-6.3	NSC-13097	-5.9
NSC-128607	-7.3	NSC-88915	-6.6	NSC-134356	-6.3	NSC-13979	-5.9
NSC-136310	-7.3	NSC-90485	-6.6	NSC-134621	-6.3	NSC-16079	-5.9
NSC-152696	-7.3	NSC-90590	-6.6	NSC-134623	-6.3	NSC-18506	-5.9
NSC-164157	-7.3	NSC-92423	-6.6	NSC-134625	-6.3	NSC-21565	-5.9
NSC-181486	-7.3	NSC-93767	-6.6	NSC-134626	-6.3	NSC-21568	-5.9
NSC-255118	-7.3	NSC-98429	-6.6	NSC-135841	-6.3	NSC-26684	-5.9
NSC-261054	-7.3	NSC-99547	-6.6	NSC-135842	-6.3	NSC-29079	-5.9
NSC-16080	-7.2	NSC-105364	-6.6	NSC-135843	-6.3	NSC-30839	-5.9
NSC-20637	-7.2	NSC-106443	-6.6	NSC-135847	-6.3	NSC-30879	-5.9
NSC-23126	-7.2	NSC-107135	-6.6	NSC-136130	-6.3	NSC-32462	-5.9
NSC-30258	-7.2	NSC-107144	-6.6	NSC-143615	-6.3	NSC-38954	-5.9
NSC-34695	-7.2	NSC-107177	-6.6	NSC-145963	-6.3	NSC-39953	-5.9
NSC-39912	-7.2	NSC-107658	-6.6	NSC-145967	-6.3	NSC-44114	-5.9
NSC-57144	-7.2	NSC-109482	-6.6	NSC-146435	-6.3	NSC-47735	-5.9
NSC-63675	-7.2	NSC-110183	-6.6	NSC-147752	-6.3	NSC-48307	-5.9
NSC-66755	-7.2	NSC-115102	-6.6	NSC-147909	-6.3	NSC-49636	-5.9
NSC-75537	-7.2	NSC-115763	-6.6	NSC-150103	-6.3	NSC-51693	-5.9
NSC-85195	-7.2	NSC-115768	-6.6	NSC-150777	-6.3	NSC-55971	-5.9
NSC-91881	-7.2	NSC-117271	-6.6	NSC-152148	-6.3	NSC-57651	-5.9
NSC-95090	-7.2	NSC-117275	-6.6	NSC-152543	-6.3	NSC-57726	-5.9
NSC-118065	-7.2	NSC-117276	-6.6	NSC-153575	-6.3	NSC-61900	-5.9
NSC-121342	-7.2	NSC-117666	-6.6	NSC-156519	-6.3	NSC-64921	-5.9
NSC-127487	-7.2	NSC-117667	-6.6	NSC-156530	-6.3	NSC-66999	-5.9
NSC-127636	-7.2	NSC-118105	-6.6	NSC-156818	-6.3	NSC-68081	-5.9
NSC-128595	-7.2	NSC-118146	-6.6	NSC-156821	-6.3	NSC-69550	-5.9
NSC-128609	-7.2	NSC-120683	-6.6	NSC-159512	-6.3	NSC-70602	-5.9
NSC-132040	-7.2	NSC-120935	-6.6	NSC-161369	-6.3	NSC-75006	-5.9

NSC-132251	-7.2	NSC-120940	-6.6	NSC-164476	-6.3	NSC-76164	-5.9
NSC-137769	-7.2	NSC-120945	-6.6	NSC-164480	-6.3	NSC-76770	-5.9
NSC-149802	-7.2	NSC-121526	-6.6	NSC-164866	-6.3	NSC-79427	-5.9
NSC-152167	-7.2	NSC-121533	-6.6	NSC-164949	-6.3	NSC-88517	-5.9
NSC-179186	-7.2	NSC-122915	-6.6	NSC-168740	-6.3	NSC-90318	-5.9
NSC-179841	-7.2	NSC-122920	-6.6	NSC-168749	-6.3	NSC-90384	-5.9
NSC-210321	-7.2	NSC-123984	-6.6	NSC-169529	-6.3	NSC-92130	-5.9
NSC-210368	-7.2	NSC-123986	-6.6	NSC-170567	-6.3	NSC-93068	-5.9
NSC-210399	-7.2	NSC-123987	-6.6	NSC-177953	-6.3	NSC-93331	-5.9
NSC-212277	-7.2	NSC-123994	-6.6	NSC-179367	-6.3	NSC-94525	-5.9
NSC-230291	-7.2	NSC-125292	-6.6	NSC-179884	-6.3	NSC-101869	-5.9
NSC-241656	-7.2	NSC-125298	-6.6	NSC-187738	-6.3	NSC-103868	-5.9
NSC-247464	-7.2	NSC-128603	-6.6	NSC-193447	-6.3	NSC-104119	-5.9
NSC-247465	-7.2	NSC-132228	-6.6	NSC-201493	-6.3	NSC-107183	-5.9
NSC-249234	-7.2	NSC-134467	-6.6	NSC-201873	-6.3	NSC-108530	-5.9
NSC-251534	-7.2	NSC-135499	-6.6	NSC-206168	-6.3	NSC-111128	-5.9
NSC-251695	-7.2	NSC-135500	-6.6	NSC-211373	-6.3	NSC-111325	-5.9
NSC-261053	-7.2	NSC-135679	-6.6	NSC-211488	-6.3	NSC-113309	-5.9
NSC-5159	-7.1	NSC-135684	-6.6	NSC-211546	-6.3	NSC-115174	-5.9
NSC-5268	-7.1	NSC-135832	-6.6	NSC-211653	-6.3	NSC-115927	-5.9
NSC-7227	-7.1	NSC-136326	-6.6	NSC-212423	-6.3	NSC-116530	-5.9
NSC-7524	-7.1	NSC-143623	-6.6	NSC-213775	-6.3	NSC-119910	-5.9
NSC-11421	-7.1	NSC-143673	-6.6	NSC-215571	-6.3	NSC-120613	-5.9
NSC-12912	-7.1	NSC-144469	-6.6	NSC-216267	-6.3	NSC-120673	-5.9
NSC-14500	-7.1	NSC-146440	-6.6	NSC-216788	-6.3	NSC-122391	-5.9
NSC-17267	-7.1	NSC-146519	-6.6	NSC-219851	-6.3	NSC-125354	-5.9
NSC-23285	-7.1	NSC-149568	-6.6	NSC-227196	-6.3	NSC-125362	-5.9
NSC-25554	-7.1	NSC-149581	-6.6	NSC-229805	-6.3	NSC-125846	-5.9
NSC-52139	-7.1	NSC-149583	-6.6	NSC-230292	-6.3	NSC-125879	-5.9
NSC-52526	-7.1	NSC-152100	-6.6	NSC-230353	-6.3	NSC-127130	-5.9
NSC-67693	-7.1	NSC-152164	-6.6	NSC-231310	-6.3	NSC-129760	-5.9
NSC-69915	-7.1	NSC-152416	-6.6	NSC-231408	-6.3	NSC-129827	-5.9
NSC-70909	-7.1	NSC-154825	-6.6	NSC-235175	-6.3	NSC-129932	-5.9
NSC-75148	-7.1	NSC-158589	-6.6	NSC-243842	-6.3	NSC-129991	-5.9
NSC-77034	-7.1	NSC-159268	-6.6	NSC-246104	-6.3	NSC-129993	-5.9
NSC-81460	-7.1	NSC-159532	-6.6	NSC-247468	-6.3	NSC-130108	-5.9
NSC-84100	-7.1	NSC-159560	-6.6	NSC-249990	-6.3	NSC-131651	-5.9
NSC-86170	-7.1	NSC-159697	-6.6	NSC-255114	-6.3	NSC-143575	-5.9
NSC-101240	-7.1	NSC-159933	-6.6	NSC-255296	-6.3	NSC-144436	-5.9
NSC-103369	-7.1	NSC-164521	-6.6	NSC-255967	-6.3	NSC-149582	-5.9
NSC-115018	-7.1	NSC-164525	-6.6	NSC-256448	-6.3	NSC-150440	-5.9
NSC-122264	-7.1	NSC-164879	-6.6	NSC-259662	-6.3	NSC-153625	-5.9
NSC-123036	-7.1	NSC-165699	-6.6	NSC-260397	-6.3	NSC-154387	-5.9
NSC-123123	-7.1	NSC-167691	-6.6	NSC-261545	-6.3	NSC-154651	-5.9
NSC-123472	-7.1	NSC-167717	-6.6	NSC-740	-6.2	NSC-156322	-5.9
NSC-123473	-7.1	NSC-167729	-6.6	NSC-1753	-6.2	NSC-156794	-5.9
NSC-125305	-7.1	NSC-167804	-6.6	NSC-4725	-6.2	NSC-160529	-5.9
NSC-128591	-7.1	NSC-173753	-6.6	NSC-6448	-6.2	NSC-162511	-5.9
NSC-128597	-7.1	NSC-177740	-6.6	NSC-8603	-6.2	NSC-164151	-5.9
NSC-128599	-7.1	NSC-179840	-6.6	NSC-11984	-6.2	NSC-164163	-5.9
NSC-128608	-7.1	NSC-180510	-6.6	NSC-12321	-6.2	NSC-167427	-5.9
NSC-129494	-7.1	NSC-193360	-6.6	NSC-12322	-6.2	NSC-169482	-5.9
NSC-135070	-7.1	NSC-193428	-6.6	NSC-13165	-6.2	NSC-171474	-5.9
NSC-135136	-7.1	NSC-196484	-6.6	NSC-15414	-6.2	NSC-173083	-5.9
NSC-141679	-7.1	NSC-200736	-6.6	NSC-21928	-6.2	NSC-174546	-5.9
NSC-144208	-7.1	NSC-203952	-6.6	NSC-22535	-6.2	NSC-175912	-5.9
NSC-144496	-7.1	NSC-210344	-6.6	NSC-23125	-6.2	NSC-179885	-5.9
NSC-150355	-7.1	NSC-210353	-6.6	NSC-28557	-6.2	NSC-179938	-5.9

NSC-157716	-7.1	NSC-210378	-6.6	NSC-30688	-6.2	NSC-180550	-5.9
NSC-171279	-7.1	NSC-210379	-6.6	NSC-30846	-6.2	NSC-180847	-5.9
NSC-174119	-7.1	NSC-210381	-6.6	NSC-30880	-6.2	NSC-184732	-5.9
NSC-176967	-7.1	NSC-211207	-6.6	NSC-35730	-6.2	NSC-185031	-5.9
NSC-194827	-7.1	NSC-211626	-6.6	NSC-36413	-6.2	NSC-203922	-5.9
NSC-196534	-7.1	NSC-212071	-6.6	NSC-36844	-6.2	NSC-204229	-5.9
NSC-201976	-7.1	NSC-216259	-6.6	NSC-37358	-6.2	NSC-210890	-5.9
NSC-211209	-7.1	NSC-218442	-6.6	NSC-40898	-6.2	NSC-211014	-5.9
NSC-211229	-7.1	NSC-221264	-6.6	NSC-43755	-6.2	NSC-211017	-5.9
NSC-211584	-7.1	NSC-221265	-6.6	NSC-45612	-6.2	NSC-212035	-5.9
NSC-220059	-7.1	NSC-225290	-6.6	NSC-46539	-6.2	NSC-215728	-5.9
NSC-226899	-7.1	NSC-230287	-6.6	NSC-47768	-6.2	NSC-219498	-5.9
NSC-244976	-7.1	NSC-231763	-6.6	NSC-49896	-6.2	NSC-224258	-5.9
NSC-254661	-7.1	NSC-239072	-6.6	NSC-51192	-6.2	NSC-227219	-5.9
NSC-260569	-7.1	NSC-239074	-6.6	NSC-52131	-6.2	NSC-246095	-5.9
NSC-262670	-7.1	NSC-239399	-6.6	NSC-55141	-6.2	NSC-249960	-5.9
NSC-1758	-7	NSC-240898	-6.6	NSC-58446	-6.2	NSC-250414	-5.9
NSC-13991	-7	NSC-241470	-6.6	NSC-67692	-6.2	NSC-263464	-5.9
NSC-15245	-7	NSC-247049	-6.6	NSC-72237	-6.2	NSC-3388	-5.8
NSC-37225	-7	NSC-247050	-6.6	NSC-72667	-6.2	NSC-7224	-5.8
NSC-38276	-7	NSC-258308	-6.6	NSC-73099	-6.2	NSC-7830	-5.8
NSC-38289	-7	NSC-3410	-6.5	NSC-73446	-6.2	NSC-12335	-5.8
NSC-43869	-7	NSC-12340	-6.5	NSC-75907	-6.2	NSC-12343	-5.8
NSC-43996	-7	NSC-12930	-6.5	NSC-76353	-6.2	NSC-31297	-5.8
NSC-47469	-7	NSC-14167	-6.5	NSC-80623	-6.2	NSC-33588	-5.8
NSC-48589	-7	NSC-22469	-6.5	NSC-80915	-6.2	NSC-35949	-5.8
NSC-53997	-7	NSC-22669	-6.5	NSC-80918	-6.2	NSC-41106	-5.8
NSC-67691	-7	NSC-23217	-6.5	NSC-84096	-6.2	NSC-41626	-5.8
NSC-74700	-7	NSC-23919	-6.5	NSC-86710	-6.2	NSC-48750	-5.8
NSC-75113	-7	NSC-27592	-6.5	NSC-88182	-6.2	NSC-55151	-5.8
NSC-75909	-7	NSC-28035	-6.5	NSC-90982	-6.2	NSC-56108	-5.8
NSC-76918	-7	NSC-29137	-6.5	NSC-92173	-6.2	NSC-56820	-5.8
NSC-80562	-7	NSC-31181	-6.5	NSC-97866	-6.2	NSC-62352	-5.8
NSC-95171	-7	NSC-35350	-6.5	NSC-97868	-6.2	NSC-69293	-5.8
NSC-96554	-7	NSC-35966	-6.5	NSC-97911	-6.2	NSC-74588	-5.8
NSC-97760	-7	NSC-36508	-6.5	NSC-98512	-6.2	NSC-74605	-5.8
NSC-103860	-7	NSC-37359	-6.5	NSC-99527	-6.2	NSC-75003	-5.8
NSC-106409	-7	NSC-37641	-6.5	NSC-101236	-6.2	NSC-75015	-5.8
NSC-106429	-7	NSC-38282	-6.5	NSC-101237	-6.2	NSC-76320	-5.8
NSC-111326	-7	NSC-39907	-6.5	NSC-101551	-6.2	NSC-77522	-5.8
NSC-114417	-7	NSC-45211	-6.5	NSC-103673	-6.2	NSC-78793	-5.8
NSC-120946	-7	NSC-49891	-6.5	NSC-103720	-6.2	NSC-79586	-5.8
NSC-120947	-7	NSC-51924	-6.5	NSC-106267	-6.2	NSC-79604	-5.8
NSC-121532	-7	NSC-55162	-6.5	NSC-107134	-6.2	NSC-80913	-5.8
NSC-123034	-7	NSC-57699	-6.5	NSC-108018	-6.2	NSC-81527	-5.8
NSC-123035	-7	NSC-66752	-6.5	NSC-108391	-6.2	NSC-81528	-5.8
NSC-123474	-7	NSC-70643	-6.5	NSC-112262	-6.2	NSC-81745	-5.8
NSC-125277	-7	NSC-70931	-6.5	NSC-114358	-6.2	NSC-85021	-5.8
NSC-125286	-7	NSC-72252	-6.5	NSC-115122	-6.2	NSC-89401	-5.8
NSC-125297	-7	NSC-73527	-6.5	NSC-115764	-6.2	NSC-93145	-5.8
NSC-128605	-7	NSC-75521	-6.5	NSC-117079	-6.2	NSC-94672	-5.8
NSC-132250	-7	NSC-79552	-6.5	NSC-117277	-6.2	NSC-97115	-5.8
NSC-139102	-7	NSC-80699	-6.5	NSC-117279	-6.2	NSC-97758	-5.8
NSC-142515	-7	NSC-81429	-6.5	NSC-117356	-6.2	NSC-99592	-5.8
NSC-143355	-7	NSC-82776	-6.5	NSC-119118	-6.2	NSC-99606	-5.8
NSC-156793	-7	NSC-84256	-6.5	NSC-119175	-6.2	NSC-102068	-5.8
NSC-159370	-7	NSC-87524	-6.5	NSC-120698	-6.2	NSC-105466	-5.8
NSC-164156	-7	NSC-87529	-6.5	NSC-120939	-6.2	NSC-109778	-5.8

NSC-164522	-7	NSC-88841	-6.5	NSC-120941	-6.2	NSC-109943	-5.8
NSC-190526	-7	NSC-91772	-6.5	NSC-121367	-6.2	NSC-111337	-5.8
NSC-210354	-7	NSC-92203	-6.5	NSC-124351	-6.2	NSC-112500	-5.8
NSC-210367	-7	NSC-93747	-6.5	NSC-125265	-6.2	NSC-115000	-5.8
NSC-211234	-7	NSC-96020	-6.5	NSC-125876	-6.2	NSC-119011	-5.8
NSC-211246	-7	NSC-96555	-6.5	NSC-126221	-6.2	NSC-120666	-5.8
NSC-211319	-7	NSC-101235	-6.5	NSC-127935	-6.2	NSC-120678	-5.8
NSC-211807	-7	NSC-101765	-6.5	NSC-127936	-6.2	NSC-121930	-5.8
NSC-231797	-7	NSC-102836	-6.5	NSC-128185	-6.2	NSC-123311	-5.8
NSC-244433	-7	NSC-103371	-6.5	NSC-129955	-6.2	NSC-123394	-5.8
NSC-1151	-6.9	NSC-104147	-6.5	NSC-132229	-6.2	NSC-123990	-5.8
NSC-5499	-6.9	NSC-105298	-6.5	NSC-132249	-6.2	NSC-125361	-5.8
NSC-7520	-6.9	NSC-105780	-6.5	NSC-134620	-6.2	NSC-126618	-5.8
NSC-12337	-6.9	NSC-106425	-6.5	NSC-135176	-6.2	NSC-129193	-5.8
NSC-12344	-6.9	NSC-106757	-6.5	NSC-136094	-6.2	NSC-129195	-5.8
NSC-13728	-6.9	NSC-107454	-6.5	NSC-136290	-6.2	NSC-134618	-5.8
NSC-13989	-6.9	NSC-109437	-6.5	NSC-143104	-6.2	NSC-136307	-5.8
NSC-16014	-6.9	NSC-111102	-6.5	NSC-143921	-6.2	NSC-138664	-5.8
NSC-17243	-6.9	NSC-115112	-6.5	NSC-144692	-6.2	NSC-139484	-5.8
NSC-21969	-6.9	NSC-115911	-6.5	NSC-144951	-6.2	NSC-142016	-5.8
NSC-23898	-6.9	NSC-117272	-6.5	NSC-145965	-6.2	NSC-147680	-5.8
NSC-27941	-6.9	NSC-117274	-6.5	NSC-149074	-6.2	NSC-148939	-5.8
NSC-39964	-6.9	NSC-119143	-6.5	NSC-152428	-6.2	NSC-150358	-5.8
NSC-43936	-6.9	NSC-119637	-6.5	NSC-152542	-6.2	NSC-153611	-5.8
NSC-50467	-6.9	NSC-120942	-6.5	NSC-156819	-6.2	NSC-156516	-5.8
NSC-51531	-6.9	NSC-121528	-6.5	NSC-159164	-6.2	NSC-162510	-5.8
NSC-66767	-6.9	NSC-121868	-6.5	NSC-159716	-6.2	NSC-163042	-5.8
NSC-71750	-6.9	NSC-121915	-6.5	NSC-162501	-6.2	NSC-173749	-5.8
NSC-72579	-6.9	NSC-122293	-6.5	NSC-162502	-6.2	NSC-174934	-5.8
NSC-75963	-6.9	NSC-122295	-6.5	NSC-162504	-6.2	NSC-176983	-5.8
NSC-89778	-6.9	NSC-122811	-6.5	NSC-162505	-6.2	NSC-180950	-5.8
NSC-90327	-6.9	NSC-122922	-6.5	NSC-164153	-6.2	NSC-205857	-5.8
NSC-90722	-6.9	NSC-122923	-6.5	NSC-164475	-6.2	NSC-206177	-5.8
NSC-91051	-6.9	NSC-123037	-6.5	NSC-165567	-6.2	NSC-210372	-5.8
NSC-91554	-6.9	NSC-124228	-6.5	NSC-168040	-6.2	NSC-214026	-5.8
NSC-91880	-6.9	NSC-125296	-6.5	NSC-168723	-6.2	NSC-226897	-5.8
NSC-92192	-6.9	NSC-125351	-6.5	NSC-168745	-6.2	NSC-230381	-5.8
NSC-96569	-6.9	NSC-125353	-6.5	NSC-171250	-6.2	NSC-230390	-5.8
NSC-103370	-6.9	NSC-126243	-6.5	NSC-171634	-6.2	NSC-231264	-5.8
NSC-103838	-6.9	NSC-126728	-6.5	NSC-172938	-6.2	NSC-231272	-5.8
NSC-113453	-6.9	NSC-128592	-6.5	NSC-177862	-6.2	NSC-231798	-5.8
NSC-114455	-6.9	NSC-132233	-6.5	NSC-179886	-6.2	NSC-254066	-5.8
NSC-117665	-6.9	NSC-132252	-6.5	NSC-180840	-6.2	NSC-254998	-5.8
NSC-118652	-6.9	NSC-132253	-6.5	NSC-187737	-6.2	NSC-1378	-5.7
NSC-119142	-6.9	NSC-132483	-6.5	NSC-187742	-6.2	NSC-2567	-5.7
NSC-119285	-6.9	NSC-133359	-6.5	NSC-187744	-6.2	NSC-3348	-5.7
NSC-120695	-6.9	NSC-133489	-6.5	NSC-204232	-6.2	NSC-3390	-5.7
NSC-120707	-6.9	NSC-134389	-6.5	NSC-204712	-6.2	NSC-4234	-5.7
NSC-120937	-6.9	NSC-134399	-6.5	NSC-205654	-6.2	NSC-12315	-5.7
NSC-120944	-6.9	NSC-135659	-6.5	NSC-205875	-6.2	NSC-12456	-5.7
NSC-121529	-6.9	NSC-135917	-6.5	NSC-209919	-6.2	NSC-23158	-5.7
NSC-122913	-6.9	NSC-135934	-6.5	NSC-211292	-6.2	NSC-30664	-5.7
NSC-122921	-6.9	NSC-138485	-6.5	NSC-211642	-6.2	NSC-32197	-5.7
NSC-123038	-6.9	NSC-140917	-6.5	NSC-213852	-6.2	NSC-32940	-5.7
NSC-123476	-6.9	NSC-141658	-6.5	NSC-216758	-6.2	NSC-35392	-5.7
NSC-123831	-6.9	NSC-143112	-6.5	NSC-216761	-6.2	NSC-36571	-5.7
NSC-123988	-6.9	NSC-143507	-6.5	NSC-216772	-6.2	NSC-38288	-5.7
NSC-123992	-6.9	NSC-143671	-6.5	NSC-218392	-6.2	NSC-47746	-5.7

NSC-126237	-6.9	NSC-145113	-6.5	NSC-219852	-6.2	NSC-51921	-5.7
NSC-126238	-6.9	NSC-146028	-6.5	NSC-219956	-6.2	NSC-64673	-5.7
NSC-128593	-6.9	NSC-146439	-6.5	NSC-224427	-6.2	NSC-69408	-5.7
NSC-134398	-6.9	NSC-147745	-6.5	NSC-229616	-6.2	NSC-75045	-5.7
NSC-140119	-6.9	NSC-148154	-6.5	NSC-230306	-6.2	NSC-79267	-5.7
NSC-140781	-6.9	NSC-148976	-6.5	NSC-230324	-6.2	NSC-80315	-5.7
NSC-140932	-6.9	NSC-149062	-6.5	NSC-231315	-6.2	NSC-81525	-5.7
NSC-143114	-6.9	NSC-149585	-6.5	NSC-234439	-6.2	NSC-86637	-5.7
NSC-144491	-6.9	NSC-150773	-6.5	NSC-236609	-6.2	NSC-87849	-5.7
NSC-152150	-6.9	NSC-152186	-6.5	NSC-237032	-6.2	NSC-90574	-5.7
NSC-156177	-6.9	NSC-152211	-6.5	NSC-238188	-6.2	NSC-90847	-5.7
NSC-156235	-6.9	NSC-154380	-6.5	NSC-238972	-6.2	NSC-99517	-5.7
NSC-159628	-6.9	NSC-155242	-6.5	NSC-239220	-6.2	NSC-99524	-5.7
NSC-159694	-6.9	NSC-155418	-6.5	NSC-244964	-6.2	NSC-109451	-5.7
NSC-165977	-6.9	NSC-156191	-6.5	NSC-244997	-6.2	NSC-114999	-5.7
NSC-166642	-6.9	NSC-158591	-6.5	NSC-245009	-6.2	NSC-116642	-5.7
NSC-170061	-6.9	NSC-162239	-6.5	NSC-245423	-6.2	NSC-116699	-5.7
NSC-170078	-6.9	NSC-162893	-6.5	NSC-247047	-6.2	NSC-119837	-5.7
NSC-174122	-6.9	NSC-164474	-6.5	NSC-248861	-6.2	NSC-121944	-5.7
NSC-174647	-6.9	NSC-168748	-6.5	NSC-250421	-6.2	NSC-123424	-5.7
NSC-176325	-6.9	NSC-168901	-6.5	NSC-250621	-6.2	NSC-124454	-5.7
NSC-177718	-6.9	NSC-170107	-6.5	NSC-251673	-6.2	NSC-125359	-5.7
NSC-179416	-6.9	NSC-174123	-6.5	NSC-251764	-6.2	NSC-125852	-5.7
NSC-196320	-6.9	NSC-201419	-6.5	NSC-254164	-6.2	NSC-126701	-5.7
NSC-196383	-6.9	NSC-202101	-6.5	NSC-254165	-6.2	NSC-128747	-5.7
NSC-196533	-6.9	NSC-208736	-6.5	NSC-254670	-6.2	NSC-129761	-5.7
NSC-210313	-6.9	NSC-208760	-6.5	NSC-255002	-6.2	NSC-129766	-5.7
NSC-210319	-6.9	NSC-210305	-6.5	NSC-256398	-6.2	NSC-129845	-5.7
NSC-210373	-6.9	NSC-211053	-6.5	NSC-260642	-6.2	NSC-129881	-5.7
NSC-210397	-6.9	NSC-211148	-6.5	NSC-263162	-6.2	NSC-135066	-5.7
NSC-211221	-6.9	NSC-211227	-6.5	NSC-264720	-6.2	NSC-135171	-5.7
NSC-211243	-6.9	NSC-211321	-6.5	NSC-8111	-6.1	NSC-137051	-5.7
NSC-212066	-6.9	NSC-211489	-6.5	NSC-8805	-6.1	NSC-138459	-5.7
NSC-212218	-6.9	NSC-211519	-6.5	NSC-10371	-6.1	NSC-139466	-5.7
NSC-213870	-6.9	NSC-212210	-6.5	NSC-10716	-6.1	NSC-139680	-5.7
NSC-222838	-6.9	NSC-212458	-6.5	NSC-12363	-6.1	NSC-144695	-5.7
NSC-226159	-6.9	NSC-213867	-6.5	NSC-13721	-6.1	NSC-151083	-5.7
NSC-227205	-6.9	NSC-213868	-6.5	NSC-18458	-6.1	NSC-156848	-5.7
NSC-229617	-6.9	NSC-213914	-6.5	NSC-21531	-6.1	NSC-159374	-5.7
NSC-230290	-6.9	NSC-216071	-6.5	NSC-21572	-6.1	NSC-163493	-5.7
NSC-244434	-6.9	NSC-217376	-6.5	NSC-23881	-6.1	NSC-171462	-5.7
NSC-247048	-6.9	NSC-226136	-6.5	NSC-24952	-6.1	NSC-176040	-5.7
NSC-254946	-6.9	NSC-230295	-6.5	NSC-28325	-6.1	NSC-204230	-5.7
NSC-259660	-6.9	NSC-231769	-6.5	NSC-30876	-6.1	NSC-209861	-5.7
NSC-1698	-6.8	NSC-233923	-6.5	NSC-30878	-6.1	NSC-211652	-5.7
NSC-5014	-6.8	NSC-235759	-6.5	NSC-30987	-6.1	NSC-211741	-5.7
NSC-5267	-6.8	NSC-238941	-6.5	NSC-31227	-6.1	NSC-217920	-5.7
NSC-7226	-6.8	NSC-239400	-6.5	NSC-33358	-6.1	NSC-219855	-5.7
NSC-12338	-6.8	NSC-243779	-6.5	NSC-37632	-6.1	NSC-241039	-5.7
NSC-15973	-6.8	NSC-244432	-6.5	NSC-41100	-6.1	NSC-245005	-5.7
NSC-16091	-6.8	NSC-251698	-6.5	NSC-43983	-6.1	NSC-250415	-5.7
NSC-17532	-6.8	NSC-254065	-6.5	NSC-44756	-6.1	NSC-257909	-5.7
NSC-27959	-6.8	NSC-260605	-6.5	NSC-47747	-6.1	NSC-262626	-5.7
NSC-31231	-6.8	NSC-264253	-6.5	NSC-49085	-6.1	NSC-14081	-5.6
NSC-34242	-6.8	NSC-4299	-6.4	NSC-52123	-6.1	NSC-16478	-5.6
NSC-37221	-6.8	NSC-5493	-6.4	NSC-55449	-6.1	NSC-32461	-5.6
NSC-38284	-6.8	NSC-5548	-6.4	NSC-63875	-6.1	NSC-45233	-5.6
NSC-38800	-6.8	NSC-7228	-6.4	NSC-68425	-6.1	NSC-47919	-5.6

NSC-39910	-6.8	NSC-7230	-6.4	NSC-69409	-6.1	NSC-63989	-5.6
NSC-45583	-6.8	NSC-12181	-6.4	NSC-72569	-6.1	NSC-67716	-5.6
NSC-50883	-6.8	NSC-12336	-6.4	NSC-75964	-6.1	NSC-76321	-5.6
NSC-51535	-6.8	NSC-12410	-6.4	NSC-80313	-6.1	NSC-77870	-5.6
NSC-52135	-6.8	NSC-15244	-6.4	NSC-85443	-6.1	NSC-81459	-5.6
NSC-52520	-6.8	NSC-15906	-6.4	NSC-87522	-6.1	NSC-81533	-5.6
NSC-54700	-6.8	NSC-17249	-6.4	NSC-89127	-6.1	NSC-82158	-5.6
NSC-66765	-6.8	NSC-17467	-6.4	NSC-90594	-6.1	NSC-82533	-5.6
NSC-66766	-6.8	NSC-19685	-6.4	NSC-92840	-6.1	NSC-87848	-5.6
NSC-68245	-6.8	NSC-21393	-6.4	NSC-96730	-6.1	NSC-92316	-5.6
NSC-72123	-6.8	NSC-24654	-6.4	NSC-97038	-6.1	NSC-97867	-5.6
NSC-79640	-6.8	NSC-28087	-6.4	NSC-97461	-6.1	NSC-99518	-5.6
NSC-87053	-6.8	NSC-31048	-6.4	NSC-99397	-6.1	NSC-99628	-5.6
NSC-90386	-6.8	NSC-37340	-6.4	NSC-99519	-6.1	NSC-106683	-5.6
NSC-91578	-6.8	NSC-38290	-6.4	NSC-99545	-6.1	NSC-109161	-5.6
NSC-98513	-6.8	NSC-38945	-6.4	NSC-99548	-6.1	NSC-109163	-5.6
NSC-103665	-6.8	NSC-39963	-6.4	NSC-99794	-6.1	NSC-114990	-5.6
NSC-104944	-6.8	NSC-42161	-6.4	NSC-99864	-6.1	NSC-116529	-5.6
NSC-104945	-6.8	NSC-42484	-6.4	NSC-104545	-6.1	NSC-117598	-5.6
NSC-105131	-6.8	NSC-43999	-6.4	NSC-106262	-6.1	NSC-120296	-5.6
NSC-105360	-6.8	NSC-45595	-6.4	NSC-106427	-6.1	NSC-122390	-5.6
NSC-108076	-6.8	NSC-49086	-6.4	NSC-109439	-6.1	NSC-125848	-5.6
NSC-113056	-6.8	NSC-50882	-6.4	NSC-109441	-6.1	NSC-128172	-5.6
NSC-115534	-6.8	NSC-50902	-6.4	NSC-109586	-6.1	NSC-132230	-5.6
NSC-116236	-6.8	NSC-51926	-6.4	NSC-111135	-6.1	NSC-136589	-5.6
NSC-116580	-6.8	NSC-55148	-6.4	NSC-113010	-6.1	NSC-151981	-5.6
NSC-117811	-6.8	NSC-56962	-6.4	NSC-113292	-6.1	NSC-154318	-5.6
NSC-118679	-6.8	NSC-57582	-6.4	NSC-115732	-6.1	NSC-154382	-5.6
NSC-120938	-6.8	NSC-57735	-6.4	NSC-115921	-6.1	NSC-155489	-5.6
NSC-120943	-6.8	NSC-58483	-6.4	NSC-116488	-6.1	NSC-157853	-5.6
NSC-121523	-6.8	NSC-63988	-6.4	NSC-117278	-6.1	NSC-162512	-5.6
NSC-122212	-6.8	NSC-64923	-6.4	NSC-117364	-6.1	NSC-179882	-5.6
NSC-122914	-6.8	NSC-69861	-6.4	NSC-117633	-6.1	NSC-180948	-5.6
NSC-123033	-6.8	NSC-74511	-6.4	NSC-118235	-6.1	NSC-180949	-5.6
NSC-126231	-6.8	NSC-76477	-6.4	NSC-118286	-6.1	NSC-204511	-5.6
NSC-126241	-6.8	NSC-77038	-6.4	NSC-118820	-6.1	NSC-210306	-5.6
NSC-130831	-6.8	NSC-77507	-6.4	NSC-119446	-6.1	NSC-227281	-5.6
NSC-135833	-6.8	NSC-79051	-6.4	NSC-120699	-6.1	NSC-230388	-5.6
NSC-137767	-6.8	NSC-82801	-6.4	NSC-121480	-6.1	NSC-234697	-5.6
NSC-138176	-6.8	NSC-83142	-6.4	NSC-123402	-6.1	NSC-247462	-5.6
NSC-140918	-6.8	NSC-84122	-6.4	NSC-123416	-6.1	NSC-251993	-5.6
NSC-142427	-6.8	NSC-86656	-6.4	NSC-123793	-6.1	NSC-264066	-5.6
NSC-142552	-6.8	NSC-87042	-6.4	NSC-125175	-6.1	NSC-5908	-5.5
NSC-142843	-6.8	NSC-87838	-6.4	NSC-126240	-6.1	NSC-21030	-5.5
NSC-144068	-6.8	NSC-87850	-6.4	NSC-127622	-6.1	NSC-45234	-5.5
NSC-144472	-6.8	NSC-89861	-6.4	NSC-128598	-6.1	NSC-47647	-5.5
NSC-144489	-6.8	NSC-90378	-6.4	NSC-129962	-6.1	NSC-70227	-5.5
NSC-146736	-6.8	NSC-90385	-6.4	NSC-134846	-6.1	NSC-99549	-5.5
NSC-151181	-6.8	NSC-90387	-6.4	NSC-135926	-6.1	NSC-103659	-5.5
NSC-156188	-6.8	NSC-90484	-6.4	NSC-140063	-6.1	NSC-106164	-5.5
NSC-156816	-6.8	NSC-91692	-6.4	NSC-140364	-6.1	NSC-112530	-5.5
NSC-159540	-6.8	NSC-94029	-6.4	NSC-141573	-6.1	NSC-117931	-5.5
NSC-160843	-6.8	NSC-103653	-6.4	NSC-144087	-6.1	NSC-121329	-5.5
NSC-163299	-6.8	NSC-106446	-6.4	NSC-144205	-6.1	NSC-129520	-5.5
NSC-164523	-6.8	NSC-106891	-6.4	NSC-144425	-6.1	NSC-129891	-5.5
NSC-167715	-6.8	NSC-109822	-6.4	NSC-145883	-6.1	NSC-132507	-5.5
NSC-168739	-6.8	NSC-115986	-6.4	NSC-146211	-6.1	NSC-134969	-5.5
NSC-168764	-6.8	NSC-118675	-6.4	NSC-146498	-6.1	NSC-144275	-5.5

NSC-174770	-6.8	NSC-120207	-6.4	NSC-146787	-6.1	NSC-144674	-5.5
NSC-174778	-6.8	NSC-120621	-6.4	NSC-150252	-6.1	NSC-155399	-5.5
NSC-179417	-6.8	NSC-120665	-6.4	NSC-156817	-6.1	NSC-156247	-5.5
NSC-179835	-6.8	NSC-120689	-6.4	NSC-158116	-6.1	NSC-159446	-5.5
NSC-187734	-6.8	NSC-120696	-6.4	NSC-159695	-6.1	NSC-172534	-5.5
NSC-187735	-6.8	NSC-121522	-6.4	NSC-159698	-6.1	NSC-186014	-5.5
NSC-193382	-6.8	NSC-121531	-6.4	NSC-160043	-6.1	NSC-202482	-5.5
NSC-195204	-6.8	NSC-123977	-6.4	NSC-160129	-6.1	NSC-210435	-5.5
NSC-201562	-6.8	NSC-125306	-6.4	NSC-162503	-6.1	NSC-219247	-5.5
NSC-205589	-6.8	NSC-125350	-6.4	NSC-163041	-6.1	NSC-229643	-5.5
NSC-210337	-6.8	NSC-125563	-6.4	NSC-164011	-6.1	NSC-231916	-5.5
NSC-210339	-6.8	NSC-125851	-6.4	NSC-164520	-6.1	NSC-239227	-5.5
NSC-210374	-6.8	NSC-126242	-6.4	NSC-165676	-6.1	NSC-241198	-5.5
NSC-210422	-6.8	NSC-126399	-6.4	NSC-167330	-6.1	NSC-247449	-5.5
NSC-210853	-6.8	NSC-127132	-6.4	NSC-170089	-6.1	NSC-4302	-5.4
NSC-210944	-6.8	NSC-127225	-6.4	NSC-170090	-6.1	NSC-13648	-5.4
NSC-211222	-6.8	NSC-129196	-6.4	NSC-170984	-6.1	NSC-23972	-5.4
NSC-211224	-6.8	NSC-129514	-6.4	NSC-173770	-6.1	NSC-31082	-5.4
NSC-211226	-6.8	NSC-129907	-6.4	NSC-174552	-6.1	NSC-91872	-5.4
NSC-211491	-6.8	NSC-131547	-6.4	NSC-174789	-6.1	NSC-96729	-5.4
NSC-211656	-6.8	NSC-132241	-6.4	NSC-174924	-6.1	NSC-100274	-5.4
NSC-211662	-6.8	NSC-132859	-6.4	NSC-179418	-6.1	NSC-105552	-5.4
NSC-212044	-6.8	NSC-134033	-6.4	NSC-180951	-6.1	NSC-114732	-5.4
NSC-212082	-6.8	NSC-134425	-6.4	NSC-184691	-6.1	NSC-120668	-5.4
NSC-212247	-6.8	NSC-134624	-6.4	NSC-187740	-6.1	NSC-132579	-5.4
NSC-235176	-6.8	NSC-134694	-6.4	NSC-197176	-6.1	NSC-135168	-5.4
NSC-240923	-6.8	NSC-135385	-6.4	NSC-201872	-6.1	NSC-144274	-5.4
NSC-246132	-6.8	NSC-135530	-6.4	NSC-205632	-6.1	NSC-167827	-5.4
NSC-246134	-6.8	NSC-142335	-6.4	NSC-205655	-6.1	NSC-204331	-5.4
NSC-249991	-6.8	NSC-143762	-6.4	NSC-205727	-6.1	NSC-55728	-5.3
NSC-250620	-6.8	NSC-144039	-6.4	NSC-209925	-6.1	NSC-68272	-5.3
NSC-262635	-6.8	NSC-144308	-6.4	NSC-211230	-6.1	NSC-86875	-5.3
NSC-264073	-6.8	NSC-148359	-6.4	NSC-211658	-6.1	NSC-96728	-5.3
NSC-1615	-6.7	NSC-149073	-6.4	NSC-212061	-6.1	NSC-113312	-5.3
NSC-9608	-6.7	NSC-150421	-6.4	NSC-216755	-6.1	NSC-115729	-5.3
NSC-12339	-6.7	NSC-153591	-6.4	NSC-216954	-6.1	NSC-115731	-5.3
NSC-12412	-6.7	NSC-153680	-6.4	NSC-217023	-6.1	NSC-120667	-5.3
NSC-13683	-6.7	NSC-156525	-6.4	NSC-224250	-6.1	NSC-123353	-5.3
NSC-13726	-6.7	NSC-156795	-6.4	NSC-226905	-6.1	NSC-134982	-5.3
NSC-14967	-6.7	NSC-156847	-6.4	NSC-229513	-6.1	NSC-149050	-5.3
NSC-16842	-6.7	NSC-157388	-6.4	NSC-235177	-6.1	NSC-154659	-5.3
NSC-22096	-6.7	NSC-163848	-6.4	NSC-238193	-6.1	NSC-186015	-5.3
NSC-25937	-6.7	NSC-164155	-6.4	NSC-238194	-6.1	NSC-226884	-5.3
NSC-30877	-6.7	NSC-164477	-6.4	NSC-241844	-6.1	NSC-237027	-5.3
NSC-31229	-6.7	NSC-166390	-6.4	NSC-249271	-6.1	NSC-66145	-5.2
NSC-37223	-6.7	NSC-167687	-6.4	NSC-260822	-6.1	NSC-83073	-5.2
NSC-37245	-6.7	NSC-167698	-6.4	NSC-264719	-6.1	NSC-94507	-5.2
NSC-39911	-6.7	NSC-168458	-6.4	NSC-2053	-6	NSC-105464	-5.2
NSC-39915	-6.7	NSC-168761	-6.4	NSC-2962	-6	NSC-125614	-5.2
NSC-41838	-6.7	NSC-170198	-6.4	NSC-3473	-6	NSC-129920	-5.2
NSC-43099	-6.7	NSC-172523	-6.4	NSC-7521	-6	NSC-134396	-5.2
NSC-43890	-6.7	NSC-173522	-6.4	NSC-7534	-6	NSC-138675	-5.2
NSC-45619	-6.7	NSC-173765	-6.4	NSC-12334	-6	NSC-139221	-5.2
NSC-50349	-6.7	NSC-175399	-6.4	NSC-12342	-6	NSC-146592	-5.2
NSC-51922	-6.7	NSC-179883	-6.4	NSC-12362	-6	NSC-150296	-5.2
NSC-52311	-6.7	NSC-180962	-6.4	NSC-15381	-6	NSC-245000	-5.2
NSC-55149	-6.7	NSC-186212	-6.4	NSC-15909	-6	NSC-261415	-5.2
NSC-61638	-6.7	NSC-203772	-6.4	NSC-18054	-6	NSC-94998	-5.1

NSC-65982	-6.7	NSC-205811	-6.4	NSC-18313	-6	NSC-102859	-5.1
NSC-66753	-6.7	NSC-209840	-6.4	NSC-18877	-6	NSC-103113	-5.1
NSC-66754	-6.7	NSC-210294	-6.4	NSC-19148	-6	NSC-129871	-5.1
NSC-70908	-6.7	NSC-210311	-6.4	NSC-21392	-6	NSC-129865	-5
NSC-76213	-6.7	NSC-210884	-6.4	NSC-36705	-6	NSC-170155	-5
NSC-76755	-6.7	NSC-211836	-6.4	NSC-37627	-6	NSC-260614	-5
NSC-78878	-6.7	NSC-212021	-6.4	NSC-39954	-6	NSC-23432	-4.9
NSC-79895	-6.7	NSC-212076	-6.4	NSC-40856	-6	NSC-114920	-4.9
NSC-80118	-6.7	NSC-213876	-6.4	NSC-42065	-6	NSC-202371	-4.9
NSC-81122	-6.7	NSC-216759	-6.4	NSC-45212	-6	NSC-260611	-4.8
NSC-84031	-6.7	NSC-216763	-6.4	NSC-45879	-6	NSC-262627	-4.8
NSC-87527	-6.7	NSC-216795	-6.4	NSC-48263	-6	NSC-192961	-4.7
NSC-90596	-6.7	NSC-219974	-6.4	NSC-48994	-6	NSC-126706	-4.5

Table S3. Consensus hits among top 100 compounds selected based on Tier-II docking scores. Top 100 compounds were chosen from the top 2000 compounds from **Table S2** against the closed states of SHP2 (wtSHP2-b and mtSHP2-b). The 73 common compounds were grouped into five categories based on their consensus ranking: top 20 common hits, top 40 common hits, top 60 common hits, top 80 common hits, and top 100 common hits.

**Top 20**		**Top 40**		**Top 60**		**Top 80**		**Top 100**	
**wtSHP2**	**mtSHP2**	**wtSHP2**	**mtSHP2**	**wtSHP2**	**mtSHP2**	**wtSHP2**	**mtSHP2**	**wtSHP2**	**mtSHP2**
NCI-51543	NCI-117812	NCI-252174	NCI-252174	NCI-39918	NCI-74702	NCI-250352	NCI-219973	NCI-118065	NCI-244996
NCI-163300	NCI-60678	NCI-143618	NCI-143618	NCI-23127	NCI-106445	NCI-39965	NCI-39917	NCI-179186	NCI-63684
NCI-60678	NCI-51543	NCI-121974	NCI-121974	NCI-153191	NCI-125304	NCI-35933	NCI-125176	NCI-128609	NCI-245023
NCI-117812	NCI-39919	NCI-15908	NCI-15908	NCI-2212	NCI-47739	NCI-82340	NCI-252124	NCI-39912	NCI-114453
NCI-39919	NCI-91579	NCI-30502	NCI-30502	NCI-153190	NCI-103663	NCI-82515	NCI-103661	NCI-75537	NCI-150357
NCI-91579	NCI-163300	NCI-84171	NCI-84171	NCI-13987	NCI-107088	NCI-244993	NCI-103858	NCI-210321	NCI-163425
		NCI-139812	NCI-139812	NCI-74702	NCI-153190	NCI-90959	NCI-180974	NCI-23126	NCI-39355
		NCI-75563	NCI-75563	NCI-106445	NCI-153191	NCI-98129	NCI-39909	NCI-210399	NCI-67586
		NCI-14757	NCI-14757	NCI-47739	NCI-23127	NCI-39909	NCI-39913	NCI-34695	NCI-179836
		NCI-173206	NCI-173206	NCI-107088	NCI-39918	NCI-39913	NCI-75911	NCI-261054	NCI-261054
		NCI-60676	NCI-60676	NCI-125304	NCI-118695	NCI-39917	NCI-82340	NCI-63684	NCI-16080
				NCI-103663	NCI-2212	NCI-39962	NCI-82515	NCI-66755	NCI-23126
				NCI-118695	NCI-13987	NCI-75911	NCI-90959	NCI-67586	NCI-34695
						NCI-103661	NCI-98129	NCI-95090	NCI-39912
						NCI-103851	NCI-103851	NCI-114453	NCI-66755
						NCI-103858	NCI-244993	NCI-121342	NCI-75537
						NCI-125176	NCI-250352	NCI-150357	NCI-95090
						NCI-180974	NCI-35933	NCI-16080	NCI-118065
						NCI-219973	NCI-39962	NCI-163425	NCI-121342
						NCI-252124	NCI-39965	NCI-179836	NCI-128609
								NCI-244996	NCI-179186
								NCI-245023	NCI-210321
								NCI-39355	NCI-210399

Table S4. Detailed parameters for the Tier I & II docking and comparative docking experiments in *allo*-site-1. The receptor coordinates used here were centroid structures from the most dominant cluster in MD simulations.

Docking Screening	Receptor (PDB code)	Grid Box Parameters						Other parameters
		Center in Å			Size in Å			
		X	Y	Z	X	Y	Z	
Tier-I	$wtSHP2^{ub}$ (2shp)	20	54	55	20	22	25	exhaustiveness=16
Tier-II	$wtSHP2^{b}$ (5ehr)	66.4169	68.4601	26.3886	27.8353	14.7666	29.2573	exhaustiveness=32
	$mtSHP2^{b}$ (6crf)	28.9514	62.5363	72.1303	21.0608	20.7446	17.5912	exhaustiveness=32
Comparative	$wtSHP2^{ub}$ (2shp)	37.905	40.728	54.182	22.5	18.75	18.75	exhaustiveness=32

Table S5. Docking results of 39 selected compounds from both *t*-BA and *h*-VI criteria to *allo*-site-1. The docking box is derived from the binding pose of SHP099 (affinity unit: kcal/mol).

***t*-BA**	**wtSHP2**	
**Label**	Molecule-ID	Affinity
C1	NSC-103858	-7.1
C2	NSC-106445	-8.6
C3	NSC-118695	-8.5
C4	NSC-121342	-7.4
C5	NSC-121974	-7.7
C6	NSC-210399	-8.1
C7	NSC-252124	-7.7
C8	NSC-261054	-8.5
C9	NSC-30502	-9.5
C10	NSC-39355	-9.1
C11	NSC-39913	-8.7
C12	NSC-39917	-8.0
C13	NSC-60678	-8.5
C14	NSC-67586	-8.2
C15	NSC-163300	-6.1
C16	NSC-23127	-4.8
C17	NSC-250352	-7.3
C18	NSC-74702	-8.7

***h*-VI**	**wtSHP**	
**Label**	Molecule-ID	Affinity
C1	NSC-14757	-7.7
C2	NSC-153191	-8.0
C3	NSC-211584	-8.9
C4	NSC-299137	-8.9
C5	NSC-371876	-8.0
C6	NSC-380323	-9.1
C7	NSC-39909	-9.0
C8	NSC-39918	-5.0
C9	NSC-618161	-8.3
C10	NSC-637201	-7.4
C11	NSC-649243	-8.2
C12	NSC-649245	-8.6
C13	NSC-665127	-8.2
C14	NSC-747599	-8.5
C15	NSC-85195	-9.4
C16	NSC-103851	-9.5
C17	NSC-252174	-7.4
C18	NSC-400376	-7.9
C19	NSC-53298	-8.5
C20	NSC-63675	-7.4
C21	NSC-74671	-7.1
